# Hydroxyethylamine Based Phthalimides as New Class of Plasmepsin Hits: Design, Synthesis and Antimalarial Evaluation

**DOI:** 10.1371/journal.pone.0139347

**Published:** 2015-10-26

**Authors:** Anil K. Singh, Sumit Rathore, Yan Tang, Nathan E. Goldfarb, Ben M. Dunn, Vinoth Rajendran, Prahlad C. Ghosh, Neelu Singh, N. Latha, Brajendra K. Singh, Manmeet Rawat, Brijesh Rathi

**Affiliations:** 1 Bioorganic Research Laboratory, Department of Chemistry, University of Delhi, Delhi, India; 2 Department of Biotechnology, All India Institute of Medical Sciences, New Delhi, India; 3 Department of Biochemistry & Molecular Biology, University of Florida College of Medicine, P.O. Box 100245, Gainesville, FL, United States of America; 4 Department of Biochemistry, University of Delhi South Campus, Dhaula Kuan, New Delhi, India; 5 Bioinformatics Center, Sri Venkateswara College, University of Delhi South Campus, Dhaula Kuan, New Delhi, India; 6 Department of Internal Medicine, 1 University of New Mexico, Albuquerque, New Mexico, United States of America; Department of Medical Lab Technology, Faculty of Applied Medical Sciences, Taibah University, SAUDI ARABIA

## Abstract

A novel class of phthalimides functionalized with privileged scaffolds was designed, synthesized and evaluated as potential inhibitors of plasmepsin 2 (K_i_: 0.99 ± 0.1 μM for **6u**) and plasmepsin 4 (K_i_: 3.3 ± 0.3 μM for **6t**), enzymes found in the digestive vacuole of the plasmodium parasite and considered as crucial drug targets. Three compounds were identified as potential candidates for further development. The listed compounds were also assayed for their antimalarial efficacy against chloroquine (CQ) sensitive strain (3D7) of *Plasmodium falciparum*. Assay of twenty seven hydroxyethylamine derivatives revealed four (**5e**, **6j**, **6o** and **6s**) as strongly active, which were further evaluated against CQ resistant strain (7GB) of *P*. *falciparum*. Compound **5e** possessing the piperidinopiperidine moiety exhibited promising antimalarial activity with an IC_50_ of 1.16 ± 0.04 μM. Further, compounds **5e**, **6j**, **6o** and **6s** exhibited low cytotoxic effect on MCF-7 cell line. Compound **6s** possessing *C*_*2*_ symmetry was identified as the least cytotoxic with significant antimalarial activity (IC_50_: 1.30 ± 0.03 μM). The combined presence of hydroxyethylamine and cyclic amines (piperazines and piperidines) was observed as crucial for the activity. The current studies suggest that hydroxyethylamine based molecules act as potent antimalarial agent and may be helpful in drug development.

## Introduction

Malaria is known as one of the most dangerous infectious disease influencing almost half of the global population and imposing a large toll on human health [1–6]. In 2010, ~219 million cases of malaria were identified that resulted in 660 thousand deaths, particularly among children [7]. The malarial parasite, *Plasmodium falciparum* alone owns the majority of lethal cases worldwide. The malarial problems in the endemic regions have reached a critical stage owing to the arrival of extensive resistance to the current antimalarial drugs and insecticide-resistant mosquitoes. The increasing resistance of *P*. *falciparum* to the available drugs, e.g., chloroquine (CQ) [8, 9], and even artemisinin derivatives [10, 11] has abridged the drug efficiency that ultimately has affected endemic regions. Only artemisinin-based combination therapies were recently considered as effective cures of malaria [12] but unfortunately the entrance of resistant malaria parasites is a serious matter. Efforts are underway to produce effective alternative drug molecules; however, until now none of the new molecules has emerged as an antimalarial drug after 1996 [13]. These factors mandate the urgent design and progress of new therapeutics with novel modes of action against multiple targets.

In the malaria parasite hemoglobin degradation in the erythrocytic stage is an inevitable process that occurs in an acidic digestive vacuole (DV) [14,15]. The important class of aspartic proteases, plasmepsins (plasmepsins 1, 2 and 4) and histo-aspartic protease (HAP) found in *P*. *falciparum*, have been identified as potential key drug targets for novel antimalarial chemotherapy [16–18]. Plasmepsin 2 and 4 have been demonstrated as crucial enzymes involved in hemoglobin degradation [19] with almost similar mode of functions. The inhibitors of plasmepsins possess significant *in vitro* and *in vivo* antimalarial effects, which suggest their suitability as potential drug candidates for antimalarial chemotherapy [20,21]. *Plasmodium* species other than *P*. *falciparum* have only plasmepsin 4 as a digestive vacuole aspartic proteinase that further advocates this enzyme as a strong target for the development of new antimalarials [22]. Recent studies revealed that not only DV plasmepsins but non-digestive vacuole (non-DV) plasmepsins also play critical roles in the survival of the parasite. The overlapped functioning of DV plasmepsins is one of the challenges in the drug development. So we can’t rule out the possibility of non-digestive vacuole plasmepsins as drug targets [23].

Hydroxyethylamine-based molecules have been explored as strong antimalarial agents and also identified as inhibitors of malarial aspartic proteases [24–28]. In hydroxyethylamines, the secondary alcohol is the essential structural element, which plays a crucial role in inhibiting the proteolytic activity of aspartic proteases by mimicking the tetrahedral intermediate during peptide bond cleavage [27,29]. Encouraged by our previous results [30], a new series of functionalized phthalimides possessing chemical variability has been designed anticipating their strong antimalarial actions. In this paper, we report the rational design and synthesis of novel hydroxyethylamine derivatives and their *in vitro* antimalarial evaluation. The cytotoxic and hemolytic effects were also studied in order to correlate and satisfy the antimalarial activity and the inhibitory activity of the new compounds against plasmepsin 2 and 4.

## Material and Methods

### Chemistry

#### General procedure for regioselective ring opening of (2*R*, 3*S*)-3-(*N*-BOC-amino)-1-oxirane-4-phenylbutane by cyclic amines to afford compounds 5b-5e

**Fig 1:**
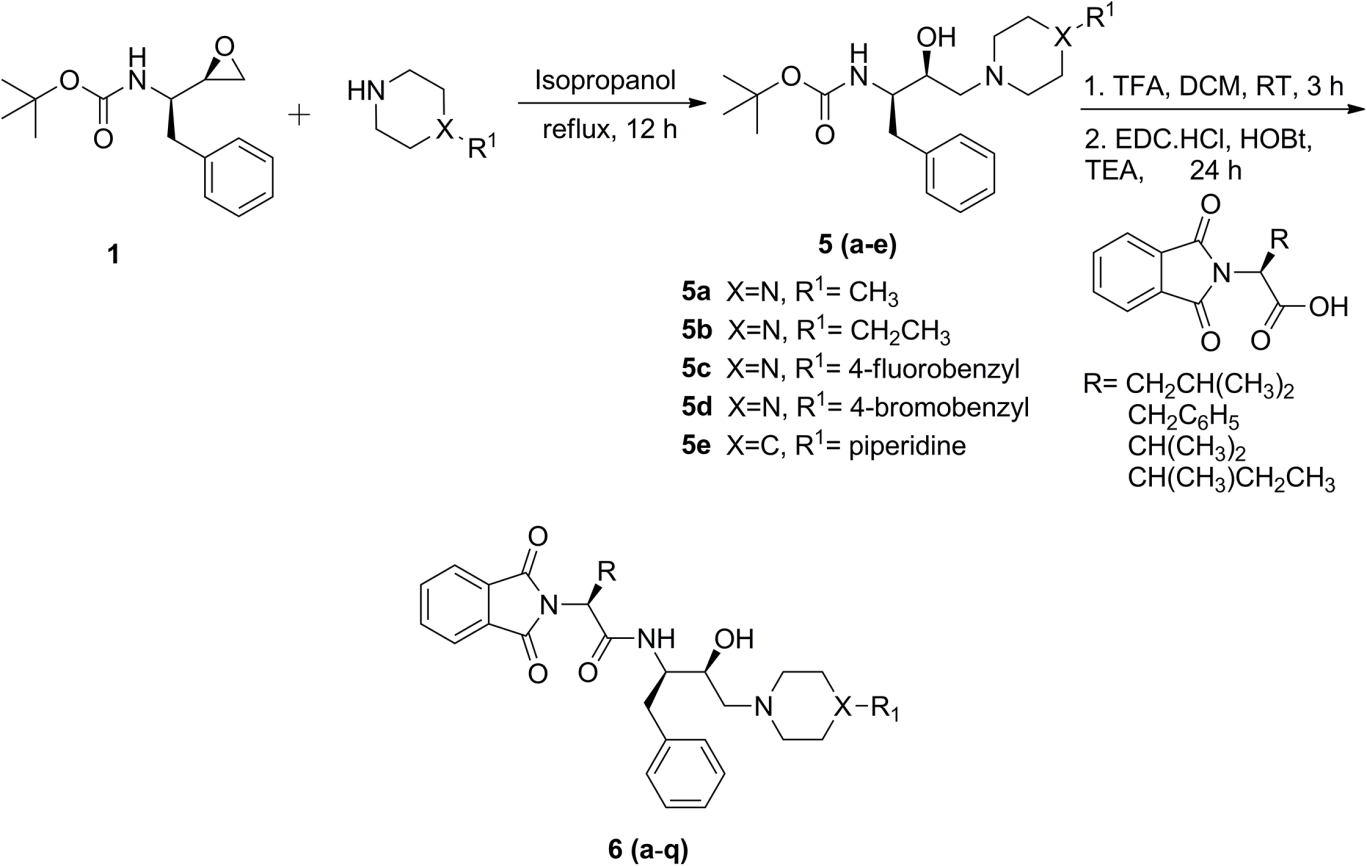
Synthesis of functionalized hydroxyethylamine derivatives.

**Fig 2:**
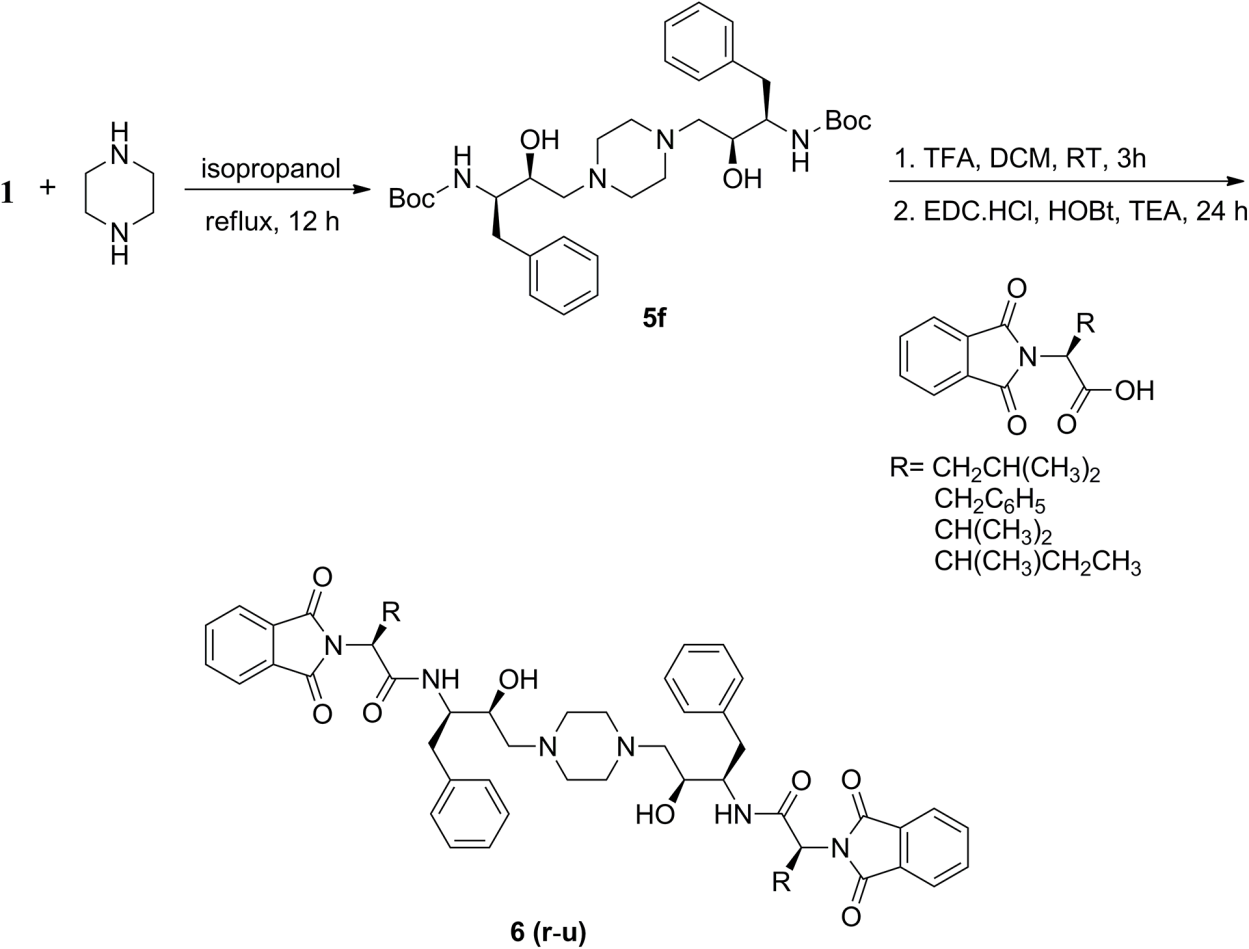
Synthesis of bis-hydroxyethylamine based phthalimides possessing *C*_*2*_ symmetry.

Synthesis of compound **5a** and **5f** was performed following the literature procedure [30]. To a solution of Boc-protected epoxide, **1** (500 mg, 1.9 mmol) in 50 mL of isopropanol, 1-methylpiperazine (0.315 mL, 2.85 mmol) was added and the contents were refluxed for 12 h (Fig 1). To obtain bis-hydroxyethylamine based molecules (**5f**, Fig 2), piperazine (82 mg, 0.95 mmol) was added to a solution of Boc-protected epoxide, **1** (500 mg, 1.9 mmol) in 50 mL of isopropanol and the contents were refluxed for 12 h. The resulting reaction mixture was concentrated under reduced pressure that afforded above mentioned compounds as white solid in good yield (Table 1) and used for further reactions without purification.

**Table 1:** Regioselective ring opening of epoxide 1 with piperazine and piperidine derivatives afforded 5a-5e.

Entry	R^1^	X	Yield (%)
**5a**	CH_3_	N	92
**5b**	CH_2_CH_3_	N	87
**5c**	4-fluorobenzyl	N	89
**5d**	4-bromobenzyl	N	89
**5e**	piperidine	C	85

#### General procedure for deprotection and coupling reaction with *N*-phthaloyl-L-amino acids to afford 6a-6u

A solution of Boc-protected hydroxyethylamines, **5a** (101 mg, 0.28 mmol) in 5 mL of dichloromethane and 1 mL of trifluoroacetic acid (TFA) was stirred at room temperature for 3 h. The solvent was evaporated under reduced pressure and the oily substance obtained was used for further reactions without purification. In another round bottomed flask, *N*-phthaloyl-L-leucine (88 mg, 0.34 mmol) in dichloromethane (20 mL) along with triethylamine (TEA) (1 mL), *N*-(3-dimethylaminopropyl)-*N*'-ethylcarbodiimide hydrochloride (EDC·HCl) (97 mg, 0.51 mmol) and anhydrous 1-hydroxybenzotriazole (HOBt) (69 mg, 0.51 mmol) were stirred at 0°C for 1 h followed by addition of Boc-deprotected hydroxyethylamine diluted with dichloromethane (3 mL). This procedure was followed to prepare compounds **6a**-**q**. The reaction mixture was stirred at 0°C for another 30 minutes and then at room temperature for 24 h. The reaction mixture was concentrated under reduced pressure. The semi-solid material obtained was diluted with 25 mL of ethylacetate and washed three times with distilled water. The organic layer obtained was dried over anhydrous Na_2_SO_4_ and solvent was evaporated under reduced pressure. The obtained compounds were purified by column chromatography using silica gel as stationary phase and chloroform-methanol (96:4) as eluent that afforded a white solid (**6a**-**q**) in fair to good yield (Table 2). The spectroscopic details are furnished in supporting information.

**Table 2:** Coupling of *N*-phthaloyl-L-amino acids with functionalized hydroxyethylamines (5a-e) afforded 6a-6q.

Entry	R	R^1^	X	Yield (%)
**6a**	CH_2_CH(CH_3_)_2_	CH_3_	N	46
**6b**	CH_2_C_6_H_5_	CH_3_	N	52
**6c**	CH(CH_3_)_2_	CH_3_	N	53
**6d**	CH(CH_3_)CH_2_CH_3_	CH_3_	N	61
**6e**	CH_2_CH(CH_3_)_2_	CH_2_CH_3_	N	59
**6f**	CH_2_C_6_H_5_	CH_2_CH_3_	N	55
**6g**	CH(CH_3_)_2_	CH_2_CH_3_	N	62
**6h**	CH(CH_3_)CH_2_CH_3_	CH_2_CH_3_	N	71
**6i**	CH_2_CH(CH_3_)_2_	4-fluorobenzyl	N	62
**6j**	CH_2_C_6_H_5_	4-fluorobenzyl	N	55
**6k**	CH(CH_3_)_2_	4-fluorobenzyl	N	71
**6l**	CH(CH_3_)CH_2_CH_3_	4-fluorobenzyl	N	43
**6m**	CH_2_CH(CH_3_)_2_	4-bromobenzyl	N	75
**6n**	CH_2_C_6_H_5_	4-bromobenzyl	N	69
**6o**	CH(CH_3_)_2_	4-bromobenzyl	N	73
**6p**	CH(CH_3_)CH_2_CH_3_	4-bromobenzyl	N	70
**6q**	CH_2_C_6_H_5_	Piperidine	C	61

To obtain **6r, 6t** and **6u**, a solution of Boc-protected bis-hydroxyethylpiperazine, **5f** (171 mg, 0.28 mmol) in 5 mL of dichloromethane and 1 mL of trifluoroacetic acid (TFA) was stirred at room temperature for 3 h. The solvent was evaporated under reduced pressure and the oily substance obtained was used for further reactions without purification. In another round bottomed flask, *N*-phthaloyl-L-amino acid (161 mg, 0.62 mmol) in dichloromethane (20 mL) along with triethylamine (TEA) (1 mL), *N*-(3-dimethylaminopropyl)-*N*'-ethylcarbodiimide hydrochloride (EDC·HCl) (177 mg, 0.93 mmol) and anhydrous1-hydroxybenzotriazole (HOBt) (125 mg, 0.93 mmol) were stirred at 0°C for 1 h followed by addition of Boc-deprotected hydroxyethylpiperazine diluted with dichloromethane (3 mL). The reaction mixture was stirred at 0°C for another 30 minutes and then at room temperature for 24 h. The reaction mixture was concentrated under reduced pressure. The semi-solid material obtained was diluted with 25 mL of ethylacetate and washed three times with distilled water. The organic layer obtained was dried over anhydrous Na_2_SO_4_ and solvent was evaporated under reduced pressure. The obtained compounds were purified by column chromatography using silica gel as stationary phase and chloroform-methanol (96:4) as eluent that afforded a white solid (**6r**-**u**) in fair to good yield (Table 3). The spectroscopic details are furnished in supporting information. Compound **6s** was prepared following literature procedure [30].

**Table 3:** Coupling of *N*-phthaloyl-L-amino acids with 5f afforded C_2_-symmetric molecules (6r-6u).

Entry	R	Yield (%)
**6r**	CH_2_CH(CH_3_)_2_	63
**6s**	CH_2_C_6_H_5_	51
**6t**	CH(CH_3_)_2_	44
**6u**	CH(CH_3_)CH_2_CH_3_	41

### Biology

#### Inhibition of plasmepsin 2 and 4

Synthetic compounds were dissolved in DMSO to make stock solutions at concentrations around 100 μM. Dilutions from these stocks were made to obtain solutions to use for studying inhibition of the plasmepsins 2 and 4 from *P*. *falciparum*. Assays of enzymatic activity were typically done using 50 micromolar substrate and 50 nanomolar enzyme at pH 4.5. The protein samples were preincubated at the assay pH for 3 minutes to allow conversion from the proenzyme to the mature enzyme form, then immediately mixed with inhibitor solutions and incubated for a further 5 minutes to permit the enzyme-inhibitor complex to form. Following this, the samples were mixed with samples of the substrate to yield a 50 μM substrate concentration in the final assay solution and immediately placed in a recording spectrophotometer to follow the reaction. The cleavage of the chromogenic substrate yields a shift in absorbance that can be quantified by the decrease in absorbance at 300 nm. Compounds were mixed with the enzymes at concentrations ranging from 10 to 50 μM range and the effect on enzyme activity studied in duplicate assays.

Compounds that demonstrated inhibition of enzymatic activity greater than 50% at the higher concentrations were then studied at concentrations as low as 1 μM to determine the extent of binding. Those compounds that showed inhibition in a concentration dependent manner were further studied in a series of assays at varying substrate concentrations (10, 20, 30, 40, 50, 60 μM substrate) and at three concentrations of inhibitory compounds including zero inhibitor concentration. A second inhibitor concentration was chosen that would yield approximately 30–35% inhibition and a third inhibitor concentration was chosen to yield approximately 65–70% inhibition. The three resulting curves of initial velocity versus substrate concentration were fit simultaneously to the competitive inhibition scheme; this yields values of V_max_, K_m_, and K_i_.

#### *In vitro* screening of synthesized compounds against *P*. *falciparum* 3D7

For compound screening, SYBR green I-based fluorescence assay was setup as described in literature [31]. Sorbitol synchronized parasites were incubated under normal culture conditions at 2% hematocrit and 1% parasitemia in the absence or presence of increasing concentrations of the compounds. Chloroquine (CQ) was used as positive control, while 0.4% DMSO was used as the negative control. After 48 h of incubation, 100 μL of SYBR Green I solution (0.2 μL of 10,000 X SYBR Green I (Invitrogen)/mL) in lysis buffer (Tris (20 mM; pH 7.5), EDTA (5 mM), saponin (0.008%; w/v), and Triton X-100 (0.08%; v/v) was added to each well and mixed twice gently with multi-channel pipette and incubated in dark at 37°C for 1 h. Fluorescence was measured with a Victor fluorescence multi-well plate reader (Perkin Elmer) with excitation and emission wavelength bands centred at 485 and 530 nm, respectively. The fluorescence counts were plotted against the drug concentration and the 50% inhibitory concentration (IC_50_) was determined by analysis of dose-response curves.

#### *In vitro* measurement of cytotoxic activity against mammalian cell line (MCF7)

Phthalimides were serially diluted in DMSO in a final concentration of 100, 25 and 6.25 μg mL^-1^ and evaluated for their % cytotoxicity by using MTT-colorimetric assay on MCF-7 cell line [32]. Cell lines were maintained in RPMI-1640 (Hi-Media, Mumbai) medium supplemented with heat inactivated FCS (10% v/v) and 100 U/mL of streptomycin and were cultured in a humidified 5% CO_2_ atmosphere at 37°C. 100 μL (~2×10^5^) of cells were seeded into wells (per well) on a 96-well plate (Nunc, Maxi Sorp) and incubated for 16 h for adherence. After 16 h, media was aspirated from the wells and the cells were washed with RPMI-1640 without FCS. 75 μL of each compound and control was added to separate wells containing cell lines and incubated at 37°C in humidified 5% CO_2_ atmosphere for 4 h. After 4 h, media containing the compounds was replaced with 200 μL of normal RPMI-1640 and cells were further incubated for 48 h under same conditions. After 48 h media was replaced by 200 μL of MTT (3-(4, 5-dimethylthiazol-2-yl)-2, 5-diphenyltetrazolium bromide, 0.5 mg/mL of RPMI-1640) and further incubated for 2 h. The formazan crystals were formed and suspended in 100 μL iso-propanol containing 0.06 M HCl and 0.5% SDS. Aliquots were drawn from each well and colour intensity was measured spectrophotometrically in an ELISA plate reader (Biotek, ELx800) at 540 nm. Untreated cells were taken as control with 100% viability and cells without addition of MTT were used as blank. The relative cell viability (%) compared to control cells was calculated by [abs] sample/ [abs] control × 100.

#### Hemolytic activity

*In vitro* hemolytic activity of all synthesized compounds was performed as per reported protocol [33] with slight modifications. In brief, human erythrocytes were collected from Rotary Blood Bank (New Delhi, India) and suspended in phosphate buffer saline (PBS). The suspension (2% hemotocrit) was incubated with different concentrations (3.125 to 100 μg/mL) of the entire compound series at 37°C for 1 h. After 1 h, erythrocytes were pelleted at 3000 g for 10 min (Remi, R-8C/BL) at 4°C. The supernatant was collected and OD_450_ was determined spectrophotometrically (Biotek, ELx800 UV spectrophotometer) using PBS as a negative control and Triton X-100 for completely lysing the cells. The percent hemolysis was calculated using the following formula: Percent hemolysis = 100-(OD of test/OD of control) × 100. The percent hemolysis was plotted against concentration of compounds for determining the dose cytotoxic to erythrocytes.

### Molecular docking

#### Preparation of Drug Targets and Binding site Analysis

The crystal structure of plasmepsin 2 and plasmepsin 4 from *Plasmodium falciparum* was obtained from the RCSB Protein Data Bank (PDB ID: plasmepsin 2 (1LF3), plasmepsin 4 (1LS5) [34]. The binding sites of the both proteins were characterized by CASTp Server [35] and Q-Site Finder [36]. The interactions of the compounds (**6p**, **6r**, **6s**, **6t** and **6u**) with both selected protein residues in the active site were taken using Maestro (Schrödinger) [37]. The targets were prepared for docking by assigning of bond orders, adding hydrogen atoms, removal of all ions, water molecules and all heteroatom from the structures which were stored. The proteins were further optimized at neutral pH and minimized using OPLS_2005 (Optimized Potentials for Liquid Simulations) force field, by converging heavy atoms to RMSD of 0.3 Å.

#### Preparation of Ligands

The three dimentional structures of compounds (**6p**, **6r**, **6s**, **6t** and **6u**) were drawn and minimized using Maestro (Schrödinger). Possible tautomers were generated using Ligprep [38] and their ADME properties were predicted using Qikprop [39].

#### Docking

The docking study was completed for selected drug targets. All possible (322) tautomers were docked using GLIDE in extra precision mode (XP) with OPLS2005 force field [40,41]. The docked compounds were analysed for binding free energy using MM-GBSA (Molecular Mechanics Generalized Born Surface Area) [42]. All the docked compounds were ranked on the basis of energy estimation.

## Results and Discussion

### Molecular Design

**Fig 3:**
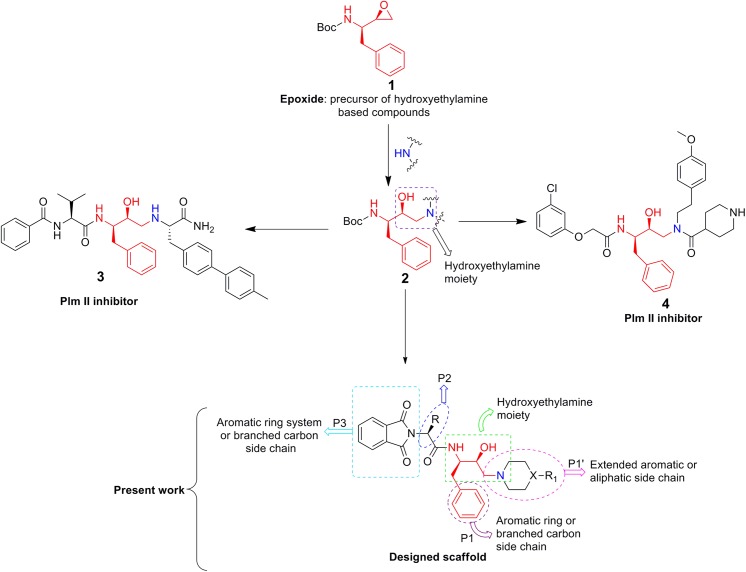
Schematic rational design of novel hydroxyethylamine based phthalimides.

The structure-based design of new bioactive molecules involves, as a crucial step, the development of a reaction strategy affording enough chemical diversity and structural variety of potentially bioactive products. In this way, not just a single molecule, but a significant part of the chemical space becomes available for the biological investigation making it possible to analyse the structure-activity relationship. Analysing the structure based design of some known potent inhibitors of aspartic proteases (plasmepsin 2) indicated that phenylalanine epoxide (**1**) moiety plays an important role. Two potential inhibitors, **3** [26] and **4** [26] are shown in Fig 3. Further, the structural analysis of these inhibitors and their interaction with plasmepsins revealed that most of the compounds possess some key structural features responsible for significant inhibition: i) the hydroxyethylamine moiety as a transition state mimicking scaffold [23]; ii) P1 and P2 positions as an aromatic system or a branched carbon side chain [43] and iii) P1’ position as an extended aromatic and aliphatic side chain. The above mentioned features were taken into consideration and a series of new hydroxyethylamine derivatives has been designed (Fig 3). The P1 position was kept constant and the P2 and P1’ moieties were varied. The P2 position was varied using different phthaloyl fragments with different natural amino-acid residues taking into account the high biological potentials of these scaffolds [44]. The medical efficacy of piperazines and piperidines has been explored by employing them as core isosteres [45–47]. Additionally, reports in the literature advocated the importance of piperazine and piperidine moieties in design of the potential candidates for antimalarial chemotherapy [48–50].

### Chemistry

The synthetic route adopted to obtain various hydroxyethylamine derivatives is described in Figs 1 and 2. The latter one represents synthesis of hydroxyethylamine based phthalimides having *C*_*2*_ symmetry. Hydroxyethylamine moiety can be obtained by regioselective ring opening of phenylalanine epoxide (Table 1) [48]. The ring opening was performed by refluxing Boc-protected (2*R*,3*S*) phenylalanine epoxide (**1**) with moderately strong nucleophiles, piperazine, 1-ethylpiperazine, 1-methylpiperazine, 4-piperidinopiperidine, 1-(4-fluorobenzyl)piperazine or 1-(4-bromobenzyl)piperazine in isopropanol for 12 h resulting in Boc-protected hydroxyethylamines (**5a**-**f**). Compounds (**5a-f**, Table 2) were deprotected using 20% trifluoroacetic acid in dichloromethane stirred at room temperature for 3 h. Various *N*-phthaloyl-L-amino acids were prepared from the fusion of L-amino acids with phthalic anhydride following the literature procedure [51]. *N*-phthaloyl-L-amino acids were recrystallized using 20% ethanol and water (v/v) and their composition was confirmed by reported melting points [52]. *N*-phthaloyl-L-amino acids and deprotected amines were coupled using coupling reagent, EDC·HCl and HOBt in the presence of triethylamine at room temperature to give the final products (**6a**-**u**) in good yields (Tables 2 and 3). The newly prepared compounds were characterised by IR, NMR (^1^H and ^13^C) and HRMS which supported the proposed structures. The spectroscopic details of compounds **5a, 5f** and **6s** were confirmed with the literature [30].

### Biology

#### Assays of inhibitory activity against plasmepsins 2 and 4

Plasmepsins, a family of aspartic proteases of malaria parasite, are known to participate in a wide variety of cellular processes essential for parasite survival. Plasmepsin 2 from *P*. *falciparum* has proven to be an excellent enzyme system for testing the inhibitory potential of new synthetic compounds. It can be prepared by recombinant methods as the inactive zymogen, proplasmepsin 2, and it converts smoothly to the mature and catalytically active enzyme, plasmepsin 2, by simple incubation at pH 4.5. Similarly, plasmepsin 4 from *P*. *falciparum* can be prepared and activated. Catalytic activity of the plasmepsins can be studied by following the change in absorbance at 300 nm as cleavage occur between a Phe and a nitroPhe in the synthetic substrate, Lys-Pro-Ile-Glu-Phe-NO_2_Phe-Arg-Leu [53]. The compounds listed in Tables 1, 2 and 3 were assayed for inhibition by preincubating with differnt concentrations of the compound with equal aliquots of either plasmepsin 2 or 4 followed by mixing with the assay substrate. All 27 compounds described in this report were tested; those that showed inhibition at 50% or greater at 10 μM were studied by additional assays where the substrate concentration was varied at three different inhibitor concentrations. Fitting of the data (see Methods) yielded values of the inhibition constant, K_i_, for the compounds and these values are reported in Table 4. It can be seen that for plasmepsin 2 compounds **6u** and **6r** have K_i_ values in the micromolar to submicromolar range. For compounds **6p** and **6t** tested against plasmepsin 4, K_i_ values were higher, in the micromolar range. These values are adequate to direct further drug optimization studies.

**Table 4:** Determination of K_i_ values for inhibition of *P*. *falciparum* plasmepsins 2 and 4. K_i_ values are reported in micromolar units.

	Plasmepsin 2	Plasmepsin 4
**6u**	0.99 ± 0.1	> 50
**6r**	1.1 ± 0.1	> 50
**6p**	> 20	27.4 ± 3.3
**6t**	> 20	3.3 ± 0.3

Derivative **6r** has a Leu-like side chain at the R position, which is expected to be the P2 position in the Schechter and Berger nomenclature [43] when binding to the active site of the plasmepsins. Compound **6u** has an Ile-like side chain in the same position. Compounds **6n** and **6s**, with a benzyl-like side chain, apparently do not fit well into the pocket where the R group would fit, which would be the S2 pocket in the enzyme active site. The benzyl group next to the–OH would fit into the S1 pocket of the active site of enzyme. Furthermore, the compounds **8a**-8**u** have the phthaloyl group on the N-terminal side of the structure and this should be accommodated in the S3 site of the plasmepsins. The phthaloyl group is much larger than the *t*-butyl group present on **5a**-**5f** and this can account for why the compounds **5a**-**5f** does not show inhibition when they are tested against plasmepsin 2 and plasmepsin 4.

Compound **6p** in Table 2 has a combination of a *sec*-butyl group in the R position (equivalent to an Ile residue in the P2 position of the synthesised compound) and a 4-bromobenzyl group in the R^1^ position. This pairing produces a K_i_ value of 27.4 μM for plasmepsin 4. Apparently, the Ile residue in the P2 position is of optimal size, as smaller or larger substituents do not work as well and hydrophobicity is required to interact with the S2 pocket of the enzyme. Consideration of the substitution in the R^1^ position shows that the bromobenzyl group is optimal; the fluorobenzyl group does not provide as strong interaction as bromobenzyl.

Compound **6t** may be compared to the other compounds in Table 3. It is seen that the isopropyl substitution (equivalent to a Valine side chain) at, possibly, the P2 position gives optimal binding with a measured K_i_ value of 3.3 μM. These results provide clues to the structure activity relationship for new inhibitors of the plasmepsins.

#### *In vitro* antimalarial activity

We have studied the antimalarial effect of all the listed molecules in asynchronized culture conditions in order to identify a putative lead structure for pharmacological applications. Compounds, **5a**-**6u** were screened for their ability to inhibit the growth of parasite activity, which was determined by the standardized SYBR green based assay [54]. The IC_50_ values for the inhibition of parasite growth by compounds (**5a**-**6u**) are listed in Table 5. The minimal inhibitory effect on the parasite growth activity was exhibited by **6q** (IC_50_: 8.54 ± 0.04 μM). It was observed that four of the compounds, **5e** (IC_50_: 1.16 ± 0.04 μM), **6j** (IC_50_: 1.33 ± 0.04 μM), **6o** (IC_50_: 1.25 ± 0.08 μM), and **6s** (IC_50_: 1.30 ± 0.03 μM) inhibited the parasite growth in a concentration-dependent manner and showed the maximum inhibition as compared with other compounds. Further, these most active members were also evaluated for their antiplasmodial activity against CQ resistant strain (7GB) of *P*. *falciparum* and a significant inhibition of the parasite growth was observed.

**Table 5:** *In vitro* antimalarial activity of hydroxyethylamine based phthalimides on the growth of *P*. *falciparum* (3D7). IC_50_ values for most potent compounds (**5e**, **6j**, **6o**, **6s)** against CQ resistant strain (7GB) over 48 h incubation are shown in the brackets.

Entry	IC_50_ values ± SE (μM)	Entry	IC_50_ values ± SE (μM)
**5a**	5.88 ± 0.23	**6j**	1.33 ± 0.04 (3.60 ± 0.06)
**5b**	5.40 ± 0.06	**6k**	4.59 ± 0.07
**5c**	2.56 ± 0.06	**6l**	3.36 ± 0.05
**5d**	4.90 ± 0.01	**6m**	6.96 ± 0.10
**5e**	1.16 ± 0.04 (2.99 ± 0.19)	**6n**	3.14 ± 0.02
**5f**	3.11 ± 0.11	**6o**	1.25 ± 0.08 (3.01 ± 0.42)
**6a**	4.61 ± 0.06	**6p**	3.28 ± 0.04
**6b**	3.41 ± 0.03	**6q**	8.54 ± 0.04
**6c**	4.58 ± 0.04	**6r**	5.12 ± 0.43
**6d**	6.33 ± 0.13	**6s**	1.30 ± 0.03 (3.15 ± 0.04)
**6e**	2.13 ± 0.02	**6t**	7.28 ± 0.43
**6f**	2.96 ± 0.43	**6u**	3.24 ± 0.06
**6g**	3.80 ± 0.03	**CQ**	0.059 ± 0.20 (1.32 ± 0.56)
**6h**	4.37 ± 0.07	**ART**	0.021 ± 0.18
**6i**	5.27 ± 0.06		

#### Effect of most potent compound (5e) on *P*. *falciparum* developmental stages

**Fig 4:**
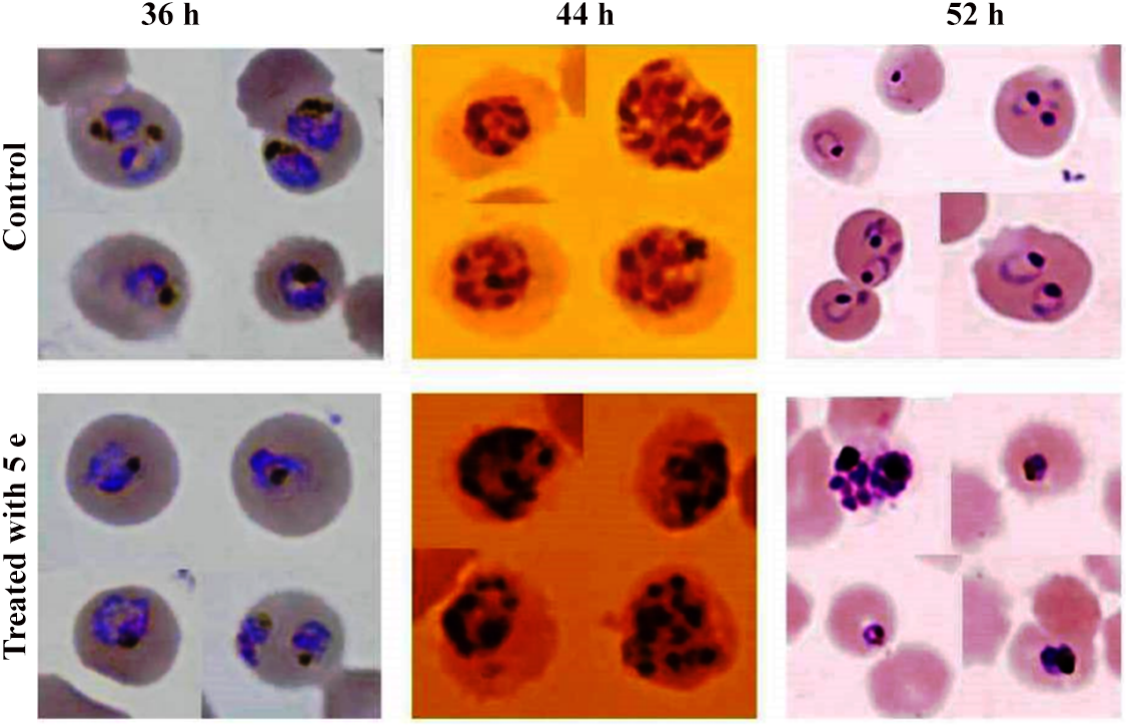
Picture showing (food vacuole abnormality) and significant inhibition of ring stage parasites when treated with 5e.

Further, in order to assess the effect of potent molecule **5e** on the parasite developmental stages (asexual), we treated tightly synchronized ring-stage parasite cultures with **5e** at 5 μM concentration and counted the parasites at different developmental stages (rings, trophozoites and schizonts) in Giemsa-stained smears at three time points (36, 44, and 52 h after treatment). The control (untreated) parasite progressed through the first cell cycle (0–48 h), increased in size and density, developed into trophozoites and schizonts and subsequently formed viable merozoites. These merozoites were able to invade fresh RBCs and form new ring-stage parasites. (Fig 4). While the drug-treated parasite set developed into the trophozoite and then continued to develop to initiate schizogony, however most of these parasites exhibited developmental arrest in the transition from early to late schizont and also showed swollen food vacuole with altered morphology at trophozoite stages (Fig 4).

**Fig 5:**
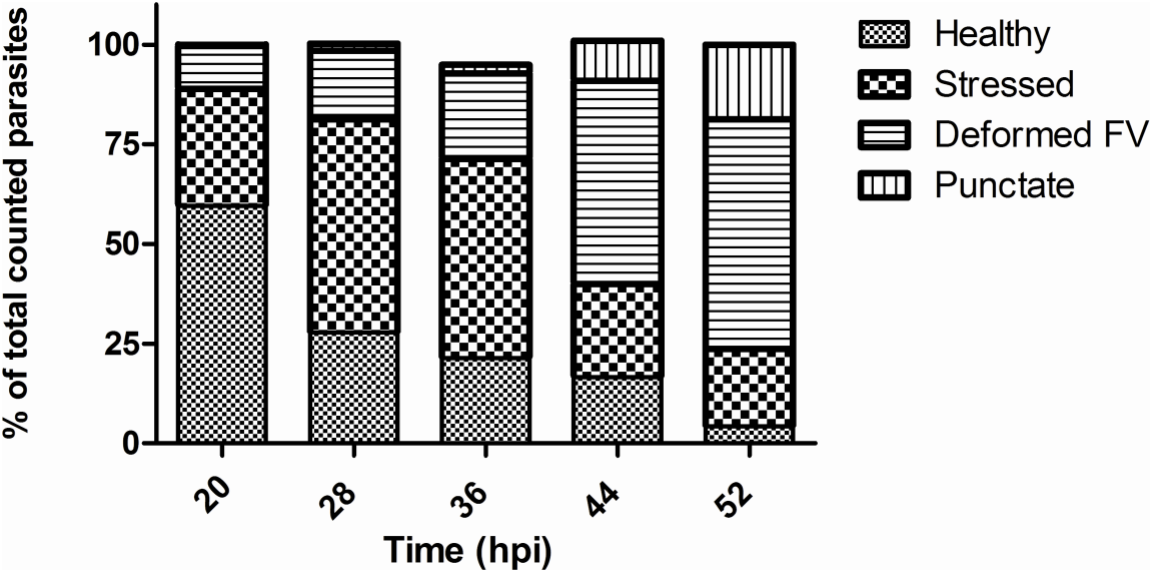
Graph showing percentage of parasite cells showing different morphological effects after treatment with 5e.

Notably, drug-treated parasites were observed with abnormal morphologies mostly in trophozoite and schizont stages. It was observed that parasites developed into multiple daughter nuclei but failed to undergo cytokinesis to develop mature merozoites (Fig 4). Therefore development of new ring-stage parasites were significantly inhibited in the drug-treated sets and showed lower total parasitemia at 52 h as compared with the control set (Fig 5).

#### Cytotoxic and hemolytic assay

Assaying all new compounds for cytotoxic effect, 13 compounds were found to display low cytotoxic effects on MCF7 cell line. Interestingly, four potent molecules with significant antimalarial activity (**5e**, **6j**, **6o** and **6s**) showed low cytotoxic effect. Compound **5f** showed maximal toxicity among all tested compounds followed by compound **5a**, **5c, 6k**, **6m** and **6q** with IC_50_ values above 8 μM. The compounds (**5e** and **6s**) were found to be less toxic. Compound, **6s** possessing *C*_*2*_ symmetry was identified as least cytotoxic as compared to other molecules.

In the course of the examination on the hemolysis of human erythrocytes, we found that 11 compounds showed lower hemotoxicity. Compound **5a**, **6p** and **6r** possessed least hemotoxic character however, **5b** possessing 4-ethylpiperazine showed highest hemotoxicity followed by **6k** and **6m**. Compounds **5e**, **6d** and **6j** possessing significant antimalarial activity, were identified with lower hemolysis. The results indicate that inhibitory effect of potent molecules at their effective concentrations is independent of cytotoxicity and hemolysis.

### Molecular Docking

Plasmepsin 2 and plasmepsin 4 [55] were selected as the drug target of *P*. *falciparum* whose structures were retrieved from PDB (3LF3 and 1LS5) and their binding sites were analyzed using CASTp, Q-Site Finder and also verified with literature survey. The top predicted sites were taken for grid formation in docking studies. The structures of compounds (**6p**, **6r**, **6s**, **6t**, **6u**) were prepared and all the possible tautomers were generated. These compounds were found to follow Lipinski's rule of 5 and show no reactive functional groups according to the ADME calculations (S1 Table).

**Fig 6:**
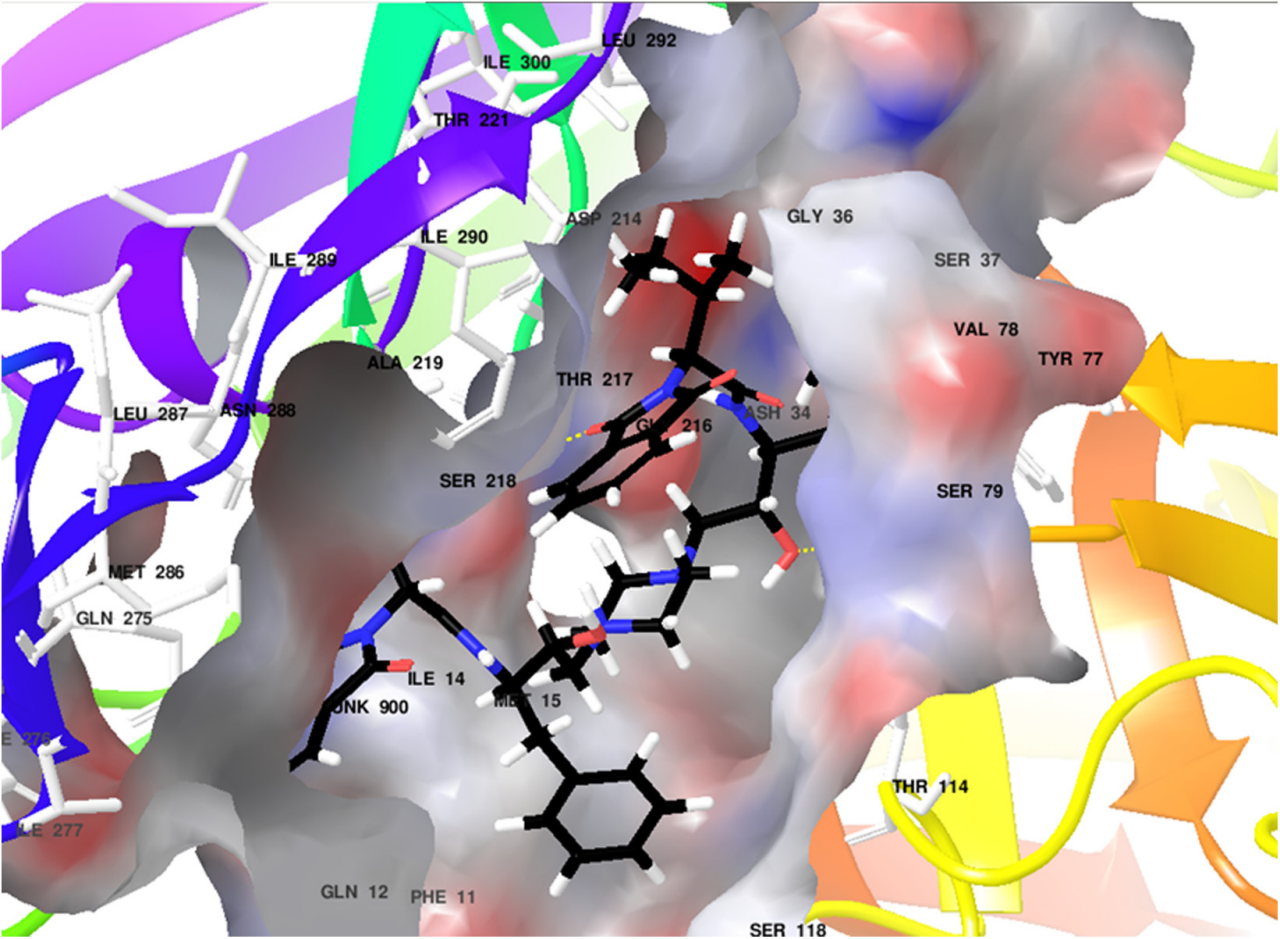
Docked image for 6u with plasmepsin 2.

**Fig 7:**
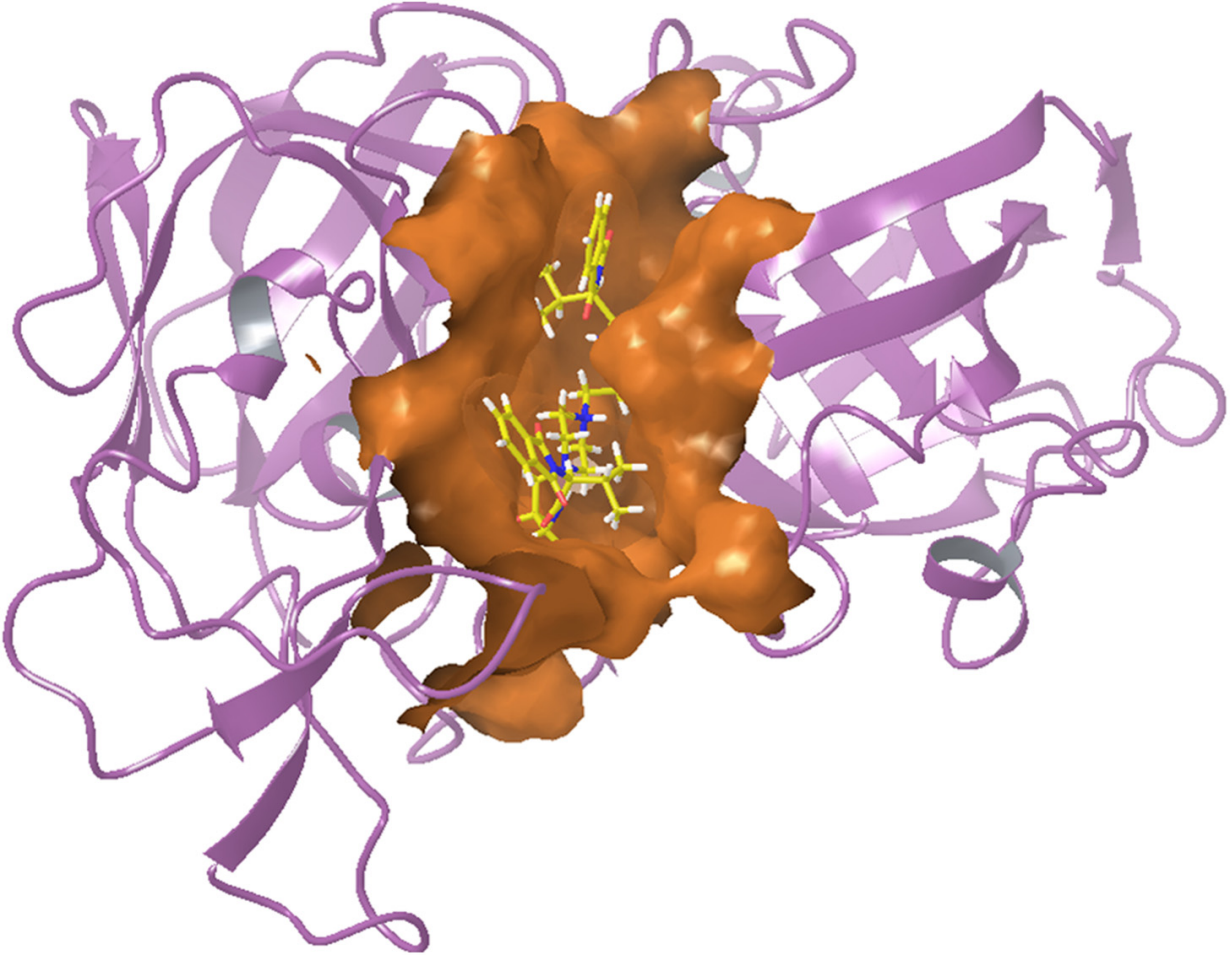
Docked image for 6t with plasmepsin 4.

Docking studies of all five compounds with plasmepsin 2 and plasmepsin 4 of *Plasmodium falciparum* were carried out using XP Glide (Schrödinger) followed by the estimation of binding free energy estimates (S2 Table and S3 Table). Based on Glide Scores and their binding free energy values, compounds (**6r**, **6u**, **6s)** were identified as top hits for plasmepsin 2 and **6r**, **6u**, **6t** for plasmepsin 4 (Figs 6 and 7).

Compound **6u** showed hydrogen bonding with plasmepsin 2 through residues Ser79 and Ser218 and hydrophobic interactions (In green, S49 Fig). Compound **6t** also showed hydrogen bonding with plasmepsin 4 through residues Ser79, Gly78 and Tyr192 including π-π stacking with Phe120 and hydrophobic interactions (In green, S50 Fig). These results advocate **6u** and **6t** as potential starting molecules for the development of new inhibitors of plasmepsin 2 and 4.

### Structure-Activity relationship

**Fig 8:**
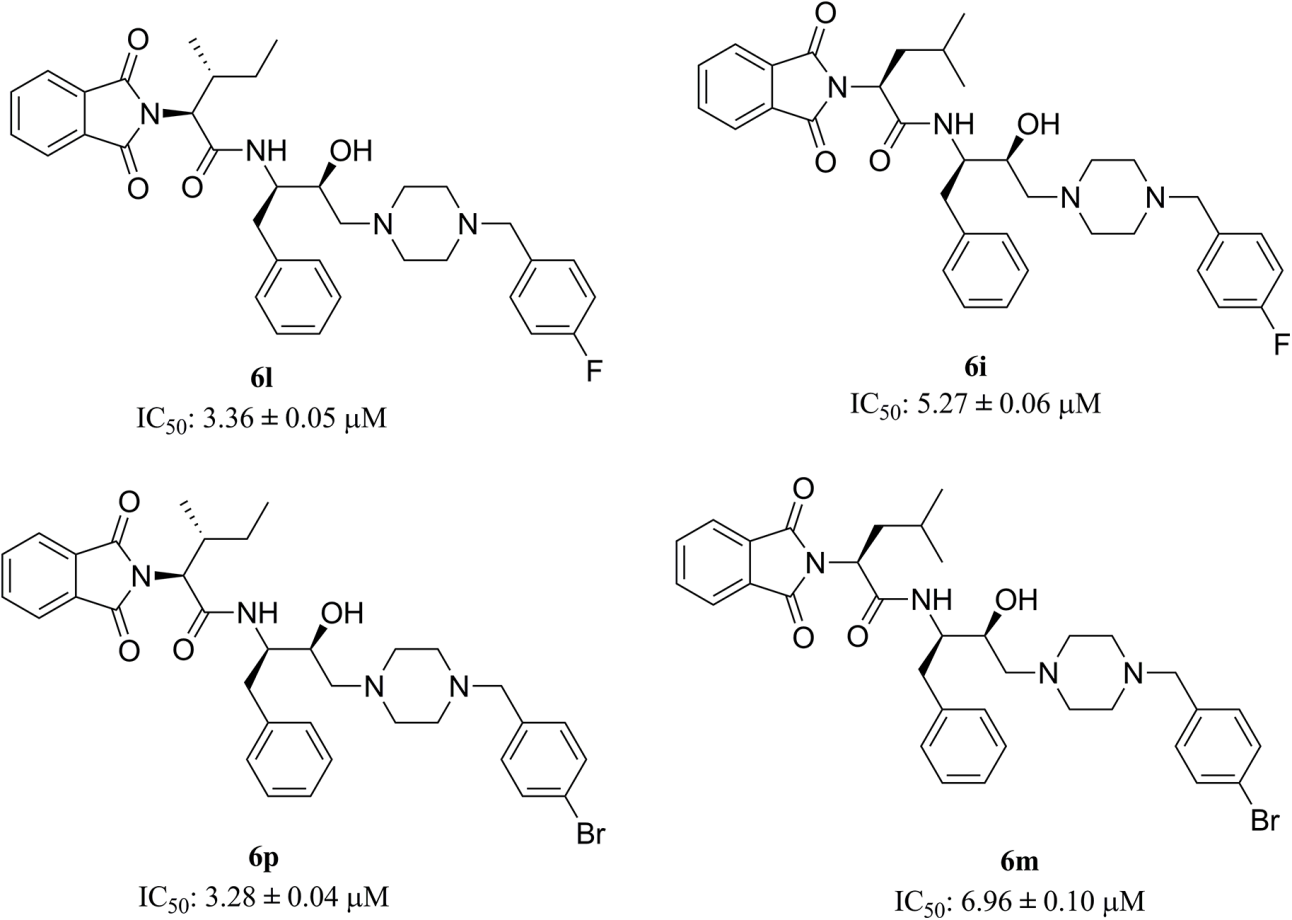
Variation of potency of positional tautomers against *P*. *falciparum*.

Functionalised hydroxyethylamine based molecules were synthesised and screened for *in vitro* antimalarial potency. During the design of molecules, scaffold **2** was kept constant and its flanking sides were diversified (Fig 3). It is evident that the substituent at P1’ position plays an important role regarding the potency of the compounds against *P*. *falciparum*. Bulkier the group, either aliphatic (**5e**) or aromatic (**6o** and **6j**), the potency is increased. Compounds with smaller aliphatic groups *viz*., methyl and ethyl directly attached to fourth position of piperazine are less effective against parasitemia suppression. At the P2 position compounds that exhibit an extended aromatic system (R = benzyl group) enhanced the lethal effect on the parasite followed by *sec*-butyl and isobutyl groups. The above observation was further reinforced when **6l** and **6p**, possessing 4-(*p-*fluorobenzyl)piperazine and 4-(*p*-bromobenzyl)piperazine, respectively, displayed higher potency (**6l**, IC_50_: 3.36 ± 0.05 μM; **6p**, IC_50_: 3.28 ± 0.04 μM) than their positional isomers **6i** (IC_50_: 5.27 ± 0.06 μM) and **6m** (IC_50_: 6.96 ± 0.10 μM), respectively as shown in Fig 8.

## Conclusion

In conclusion, we have reported the antimalarial activity of hydroxyethylamine based phthalimides. Significantly, the obtained functionalized small molecules were found to provide a new avenue for the discovery of antimalarial agents against CQ resistant strain of *P*. *falciparum*. Almost all the molecules were active as anticipated owing to the presence of hydroxyethylamine and piperazine or piperidine moieties. The lower K_i_ values of all the screened compounds showed their efficacy as inhibitors of plasmepsin 2 (**6u**) and plasmepsin 4 (**6t**). The experiments demonstrated that the treatment of **5e** inhibits the parasite to transit from early to late schizont stage, which was evidenced by absence of ring stage. Among all the compounds tested, **5e** (an intermediate) was proved to be the most potent compound. However, compound **5e** did not inhibit the activity of plasmepsins (2 and 4), which advocates the possibility of other drug targets like non-digestive vacuole plasmepsins which, in fact, might be the crucial drug targets according to the current understanding of plasmepsin biology. As there is lack of potent inhibitors of non-DV plasmepsins, we can’t rule out **5e** and similar compounds as inhibitor of non-DV plasmepsin, which has been proved as good recipe for research in the area of plasmepsin medicinal chemistry.

## Supporting Information

S1 Fig ^1^H NMR spectra of 5b. (TIF)

S2 Fig ^13^C NMR spectra of 5b. (TIF)

S3 Fig ^1^H NMR spectra of 5c. (TIF)

S4 Fig ^13^C NMR spectra of 5c. (TIF)

S5 Fig ^1^H NMR spectra of 5d. (TIF)

S6 Fig ^13^C NMR spectra of 5d. (TIF)

S7 Fig ^1^H NMR spectra of 5e. (TIF)

S8 Fig ^13^C NMR spectra of 5e. (TIF)

S9 Fig ^1^H NMR spectra of 6a. (TIF)

S10 Fig ^13^C NMR spectra of 6a. (TIF)

S11 Fig ^1^H NMR spectra of 6b. (TIF)

S12 Fig ^13^C NMR spectra of 6b. (TIF)

S13 Fig ^1^H NMR spectra of 6c. (TIF)

S14 Fig ^13^C NMR spectra of 6c. (TIF)

S15 Fig ^1^H NMR spectra of 6d. (TIF)

S16 Fig ^13^C NMR spectra of 6d. (TIF)

S17 Fig ^1^H NMR spectra of 6e. (TIF)

S18 Fig ^13^C NMR spectra of 6e. (TIF)

S19 Fig ^1^H NMR spectra of 6f. (TIF)

S20 Fig ^13^C NMR spectra of 6f. (TIF)

S21 Fig ^1^H NMR spectra of 6g. (TIF)

S22 Fig ^13^C NMR spectra of 6g. (TIF)

S23 Fig ^1^H NMR spectra of 6h. (TIF)

S24 Fig ^13^C NMR spectra of 6h. (TIF)

S25 Fig ^1^H NMR spectra of 6i. (TIF)

S26 Fig ^13^C NMR spectra of 6i. (TIF)

S27 Fig ^1^H NMR spectra of 6j. (TIF)

S28 Fig ^13^C NMR spectra of 6j. (TIF)

S29 Fig ^1^H NMR spectra of 6k. (TIF)

S30 Fig ^13^C NMR spectra of 6k. (TIF)

S31 Fig ^1^H NMR spectra of 6l. (TIF)

S32 Fig ^13^C NMR spectra of 6l. (TIF)

S33 Fig ^1^H NMR spectra of 6m. (TIF)

S34 Fig ^13^C NMR spectra of 6m. (TIF)

S35 Fig ^1^H NMR spectra of 6n. (TIF)

S36 Fig ^13^C NMR spectra of 6n. (TIF)

S37 Fig ^1^H NMR spectra of 6o. (TIF)

S38 Fig ^13^C NMR spectra of 6o. (TIF)

S39 Fig ^1^H NMR spectra of 6p. (TIF)

S40 Fig ^13^C NMR spectra of 6p. (TIF)

S41 Fig ^1^H NMR spectra of 6q. (TIF)

S42 Fig ^13^C NMR spectra of 6q. (TIF)

S43 Fig ^1^H NMR spectra of 6r. (TIF)

S44 Fig ^13^C NMR spectra of 6r. (TIF)

S45 Fig ^1^H NMR spectra of 6t. (TIF)

S46 Fig ^13^C NMR spectra of 6t. (TIF)

S47 Fig ^1^H NMR spectra of 6u. (TIF)

S48 Fig ^13^C NMR spectra of 6u. (TIF)

S49 Fig Protein-ligand contacts of plasmepsin 2 (PDB ID: 1LF3) with top hit 6u. (TIF)

S50 Fig Protein-ligand contacts of plasmepsin 4 (PDB ID: 1LS5) with top hit 6t. (TIF)

S1 Table Physicochemical properties of compounds (5e, 6p, 6r, 6s, 6t, and 6u). (DOCX)

S2 Table The XP GScore and binding free energy values of potent compounds docked to plasmepsin-2 (PDB ID: 2LF3). (DOCX)

S3 Table The XP GScore and binding free energy values of potent compounds docked to plasmepsin-4 (PDB ID: 1LS5). (DOCX)

S1 Text General considerations. (DOCX)

S2 Text Spectroscopic data of synthesised compounds (5b-6r, 6t and 6u). (DOC)

S3 Text Reference. (DOCX)
