# Supplementary figures and images for: Hydroxyethylamine Based Phthalimides as New Class of Plasmepsin Hits: Design, Synthesis and Antimalarial Evaluation
Source: PLoS One. 2015 Oct 26;10(10):e0139347. doi: 10.1371/journal.pone.0139347 (PMC4621027; doi:10.1371/journal.pone.0139347)

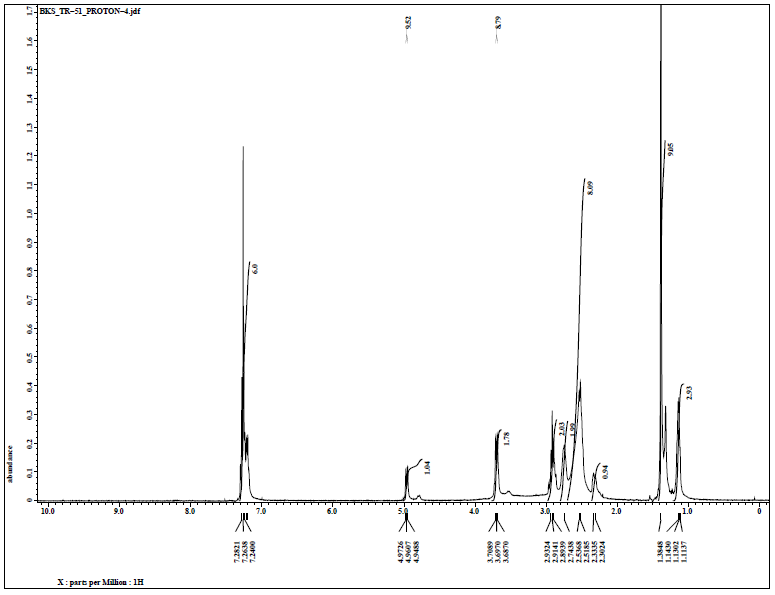

Supplement: S1 Fig — (TIF) [file pone.0139347.s001.tif]

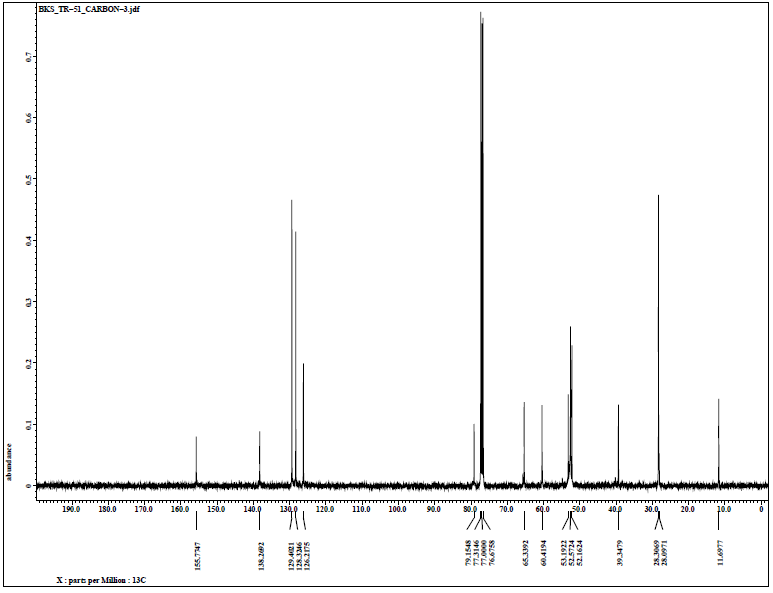

Supplement: S2 Fig — (TIF) [file pone.0139347.s002.tif]

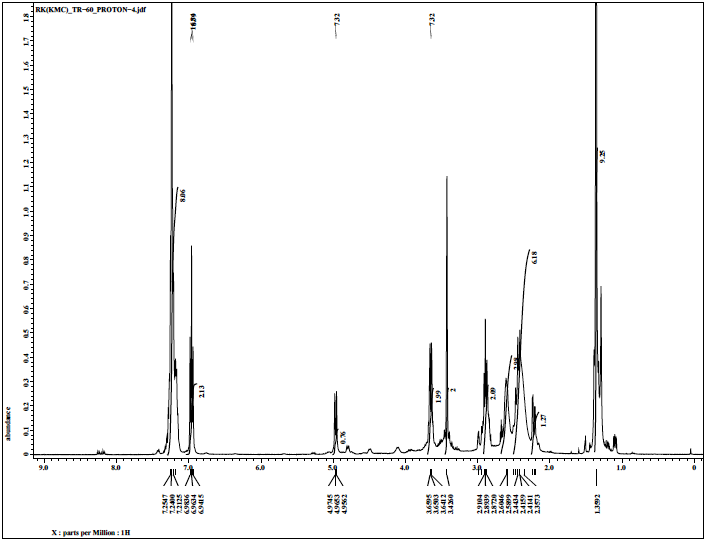

Supplement: S3 Fig — (TIF) [file pone.0139347.s003.tif]

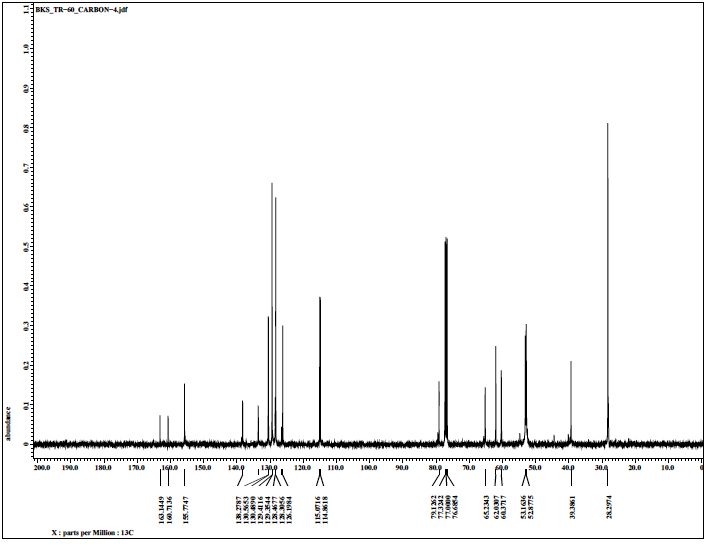

Supplement: S4 Fig — (TIF) [file pone.0139347.s004.tif]

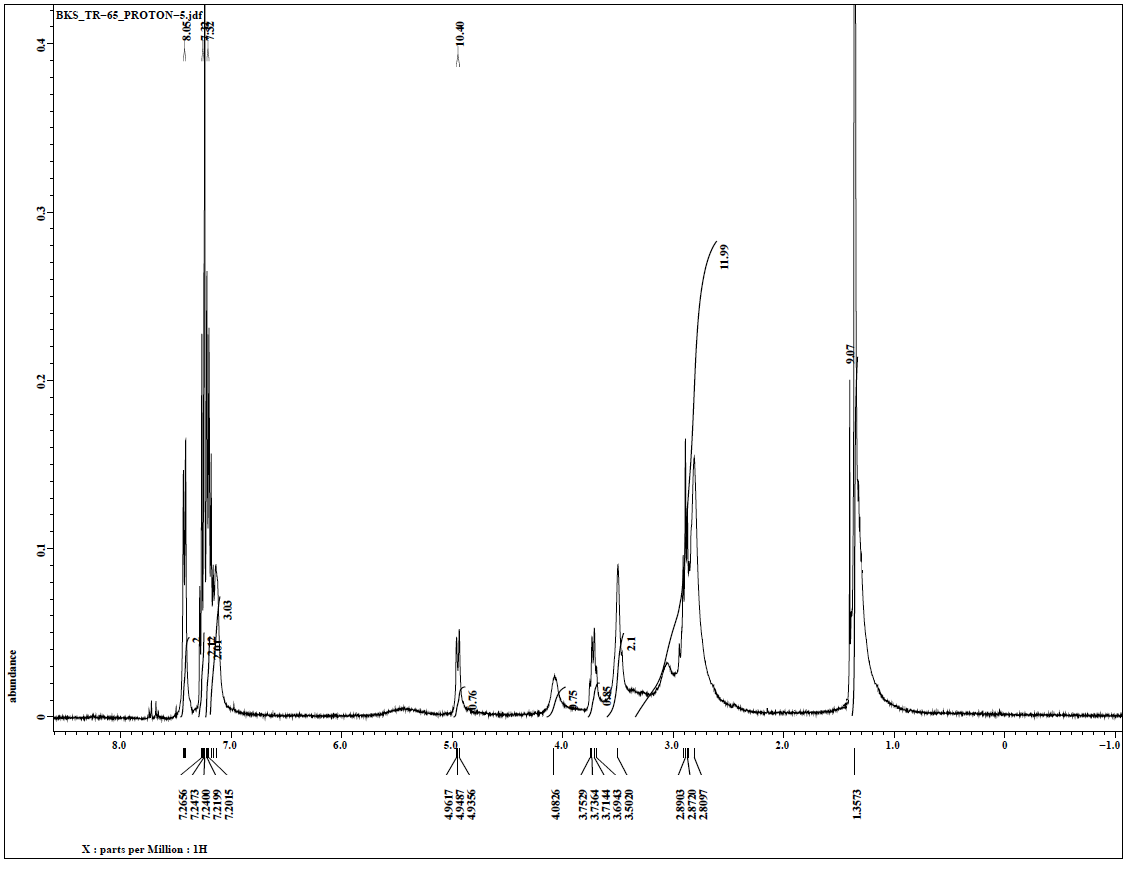

Supplement: S5 Fig — (TIF) [file pone.0139347.s005.tif]

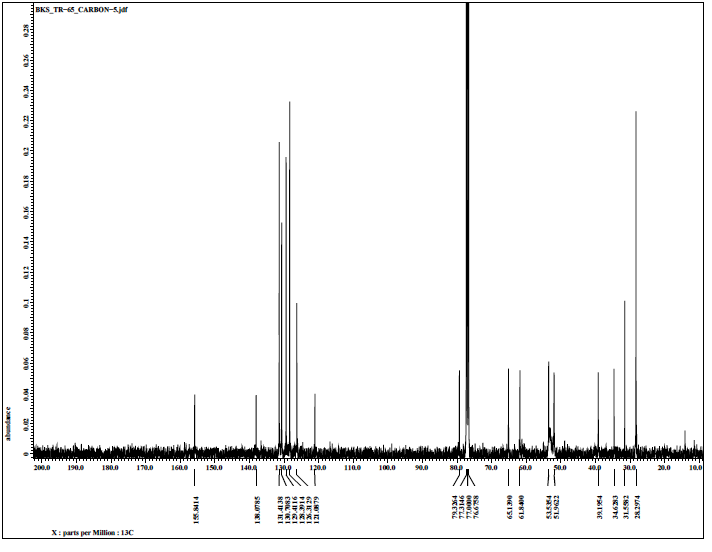

Supplement: S6 Fig — (TIF) [file pone.0139347.s006.tif]

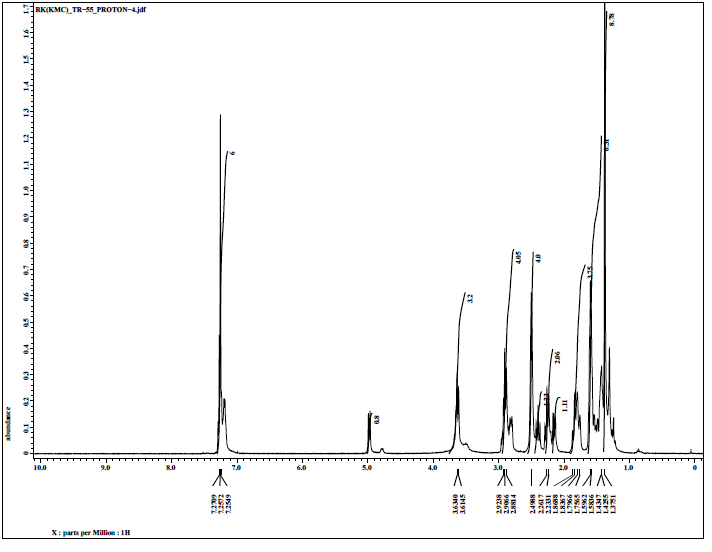

Supplement: S7 Fig — (TIF) [file pone.0139347.s007.tif]

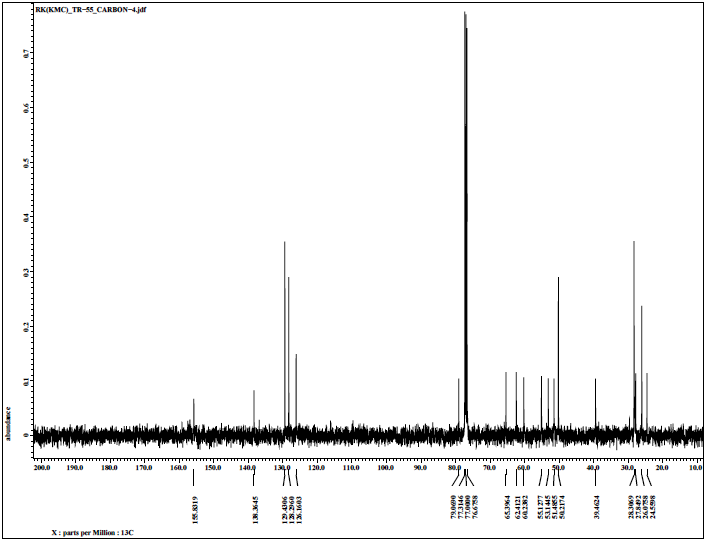

Supplement: S8 Fig — (TIF) [file pone.0139347.s008.tif]

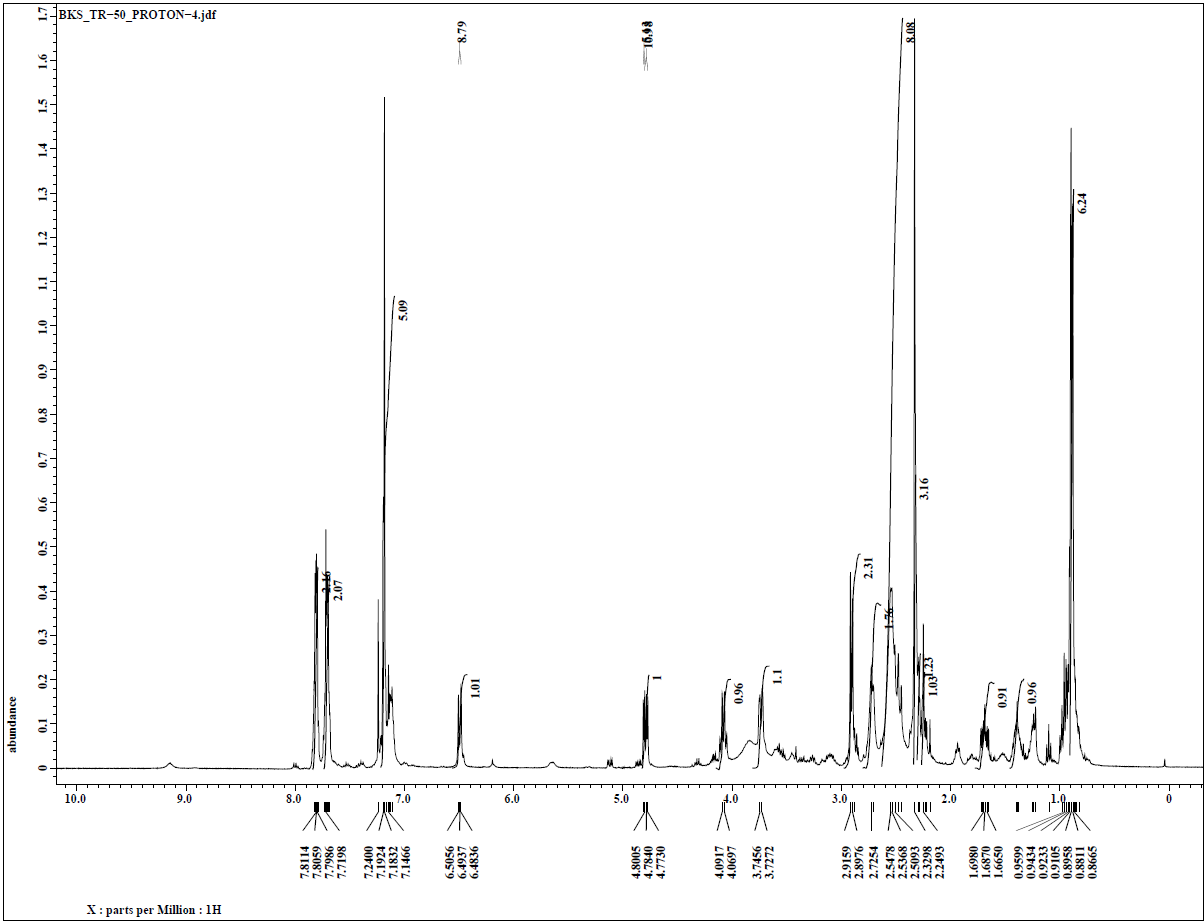

Supplement: S9 Fig — (TIF) [file pone.0139347.s009.tif]

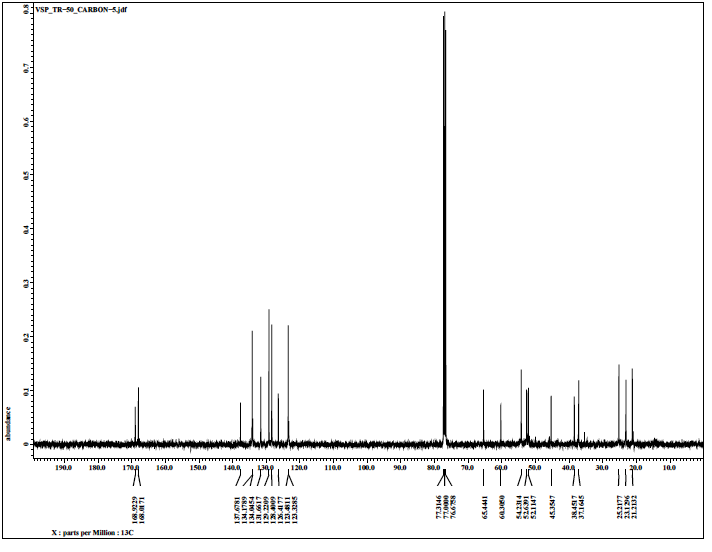

Supplement: S10 Fig — (TIF) [file pone.0139347.s010.tif]

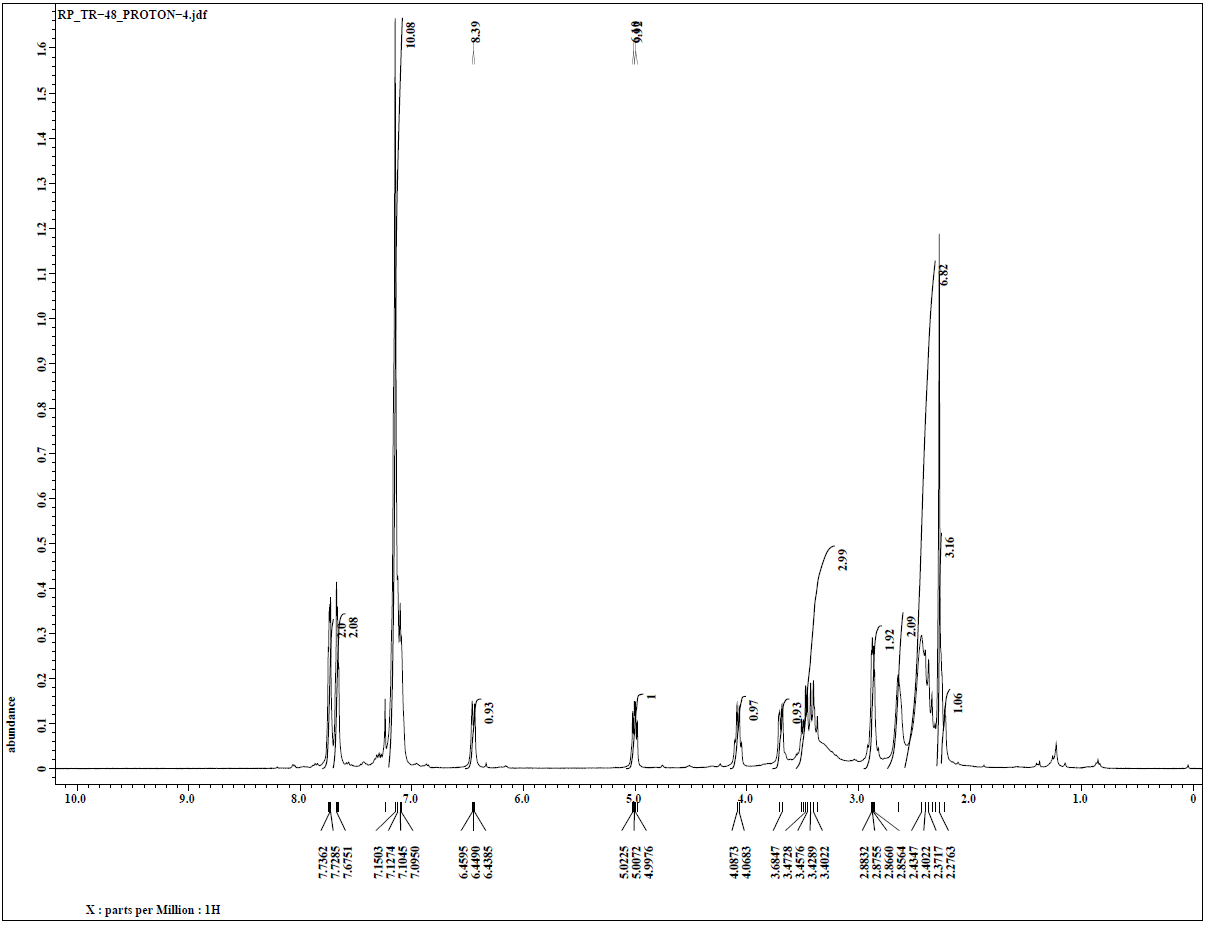

Supplement: S11 Fig — (TIF) [file pone.0139347.s011.tif]

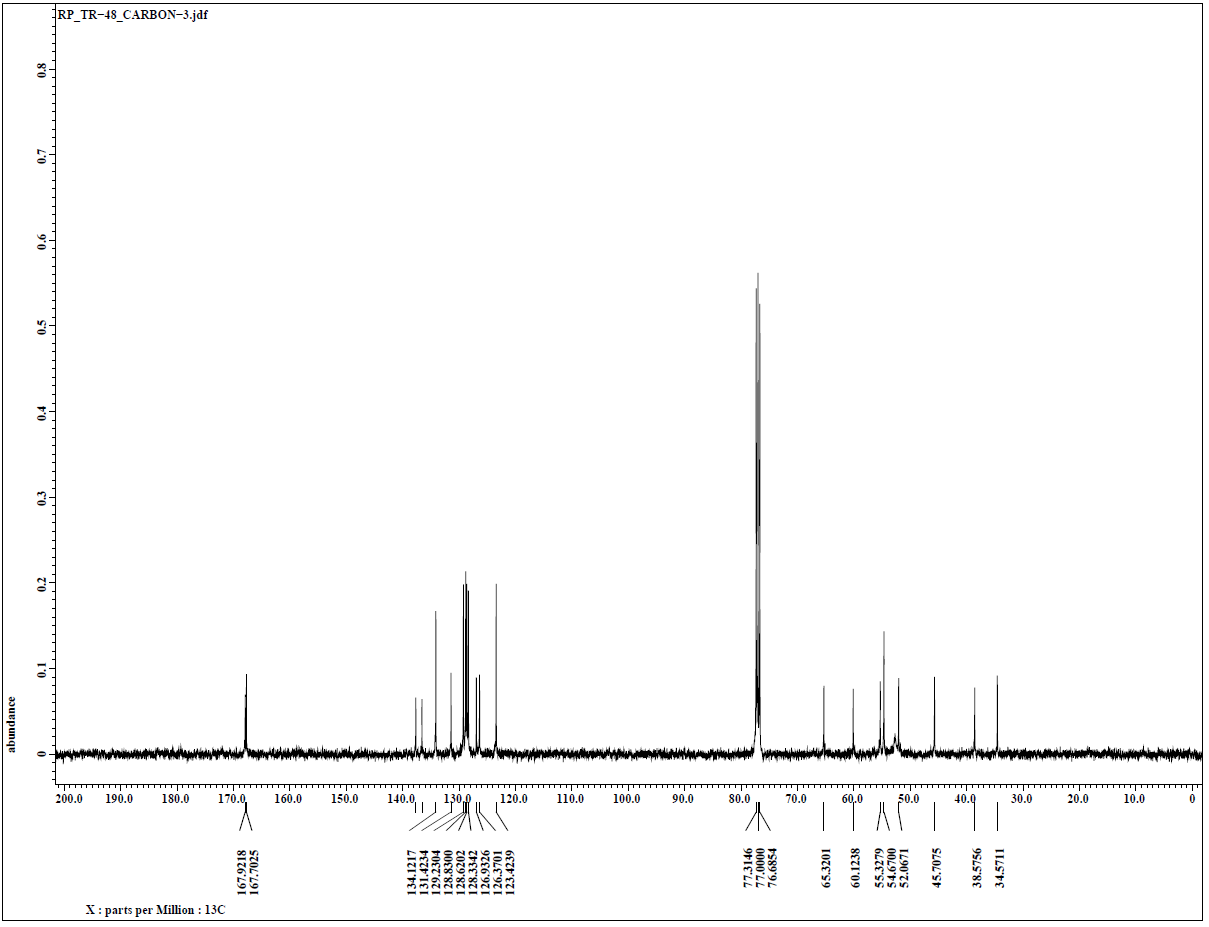

Supplement: S12 Fig — (TIF) [file pone.0139347.s012.tif]

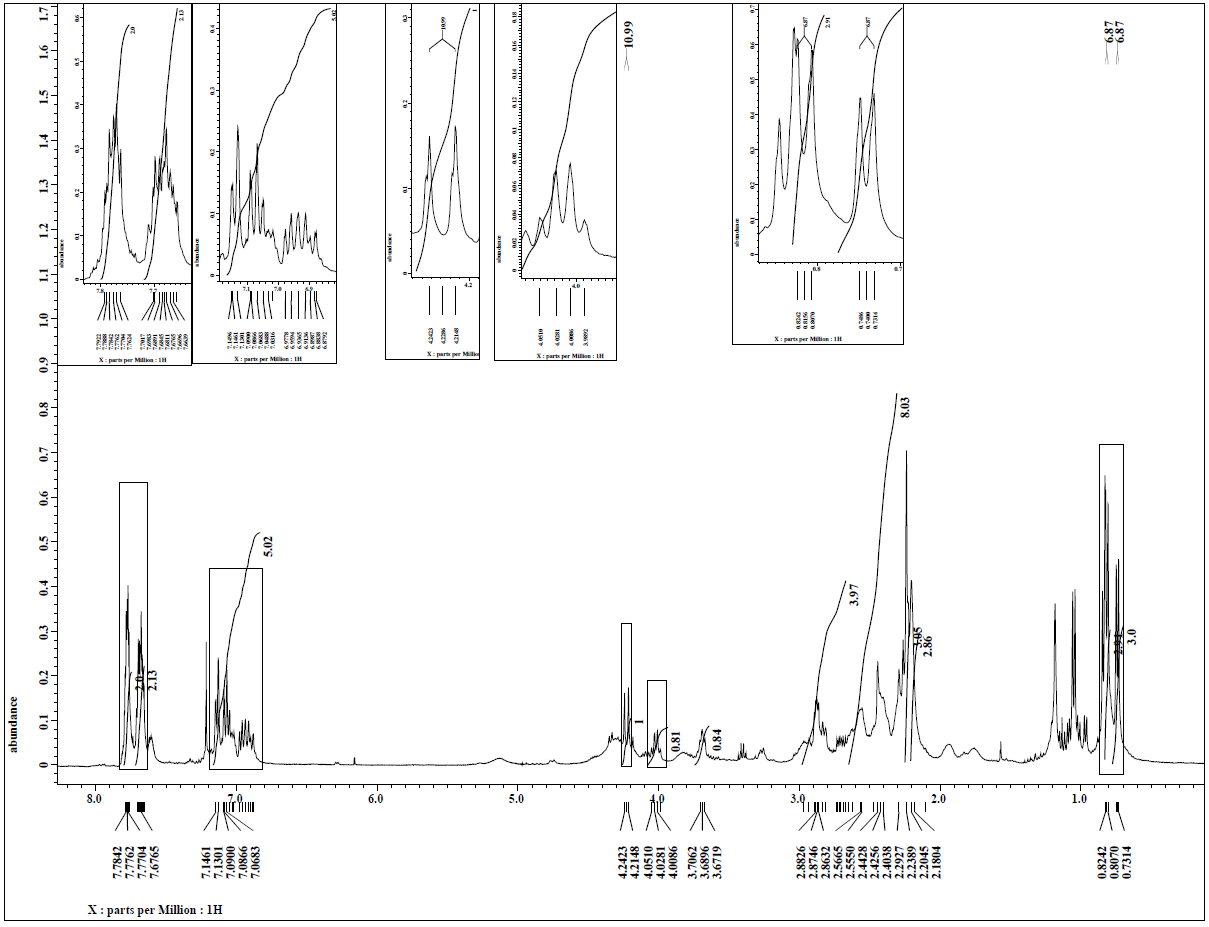

Supplement: S13 Fig — (TIF) [file pone.0139347.s013.tif]

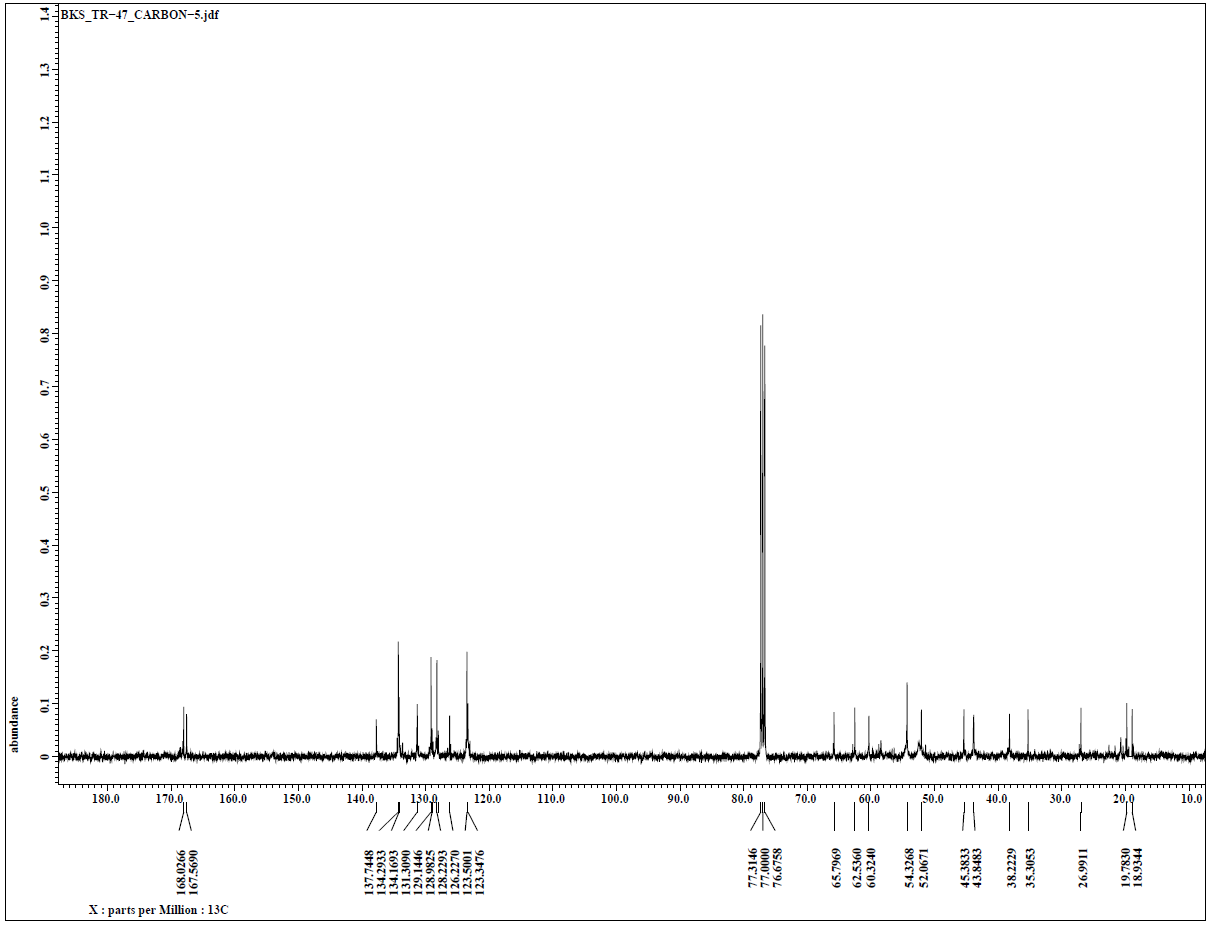

Supplement: S14 Fig — (TIF) [file pone.0139347.s014.tif]

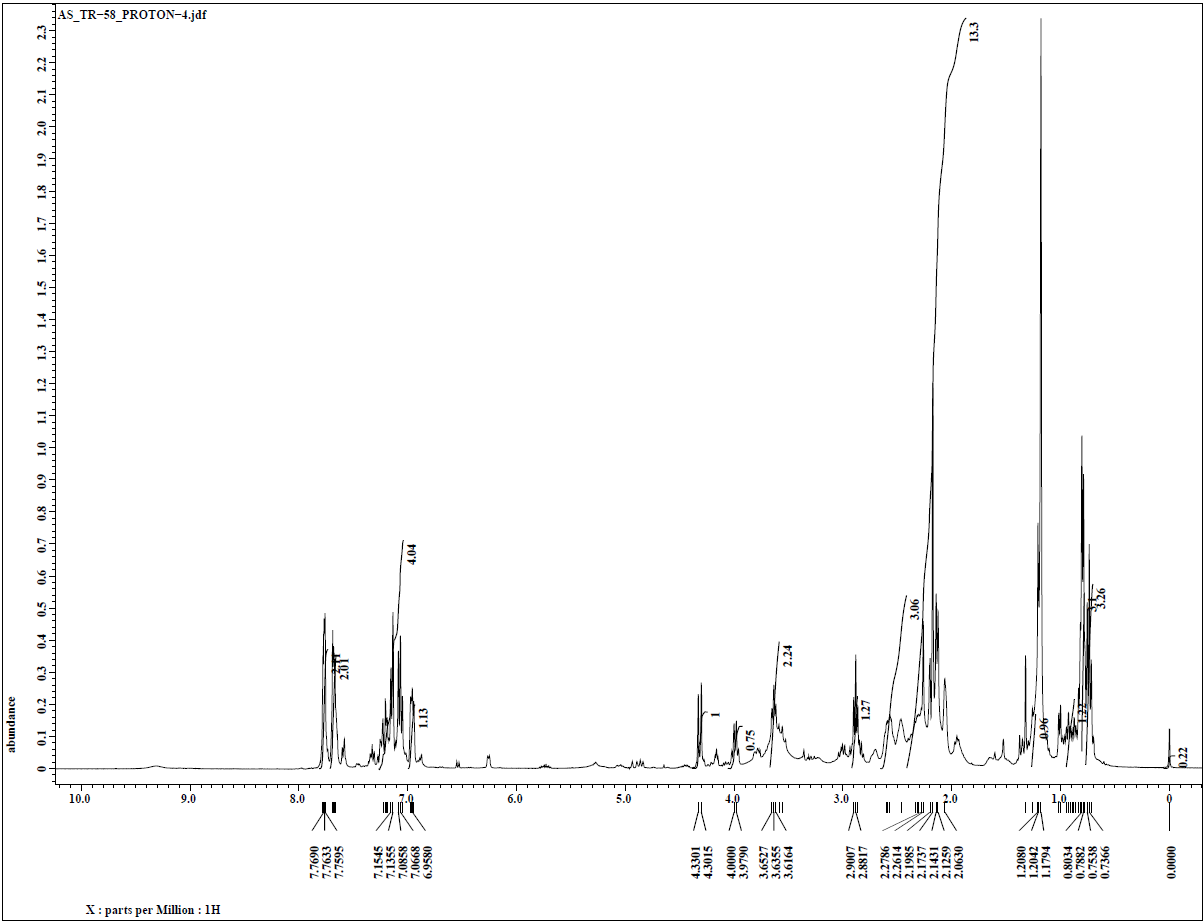

Supplement: S15 Fig — (TIF) [file pone.0139347.s015.tif]

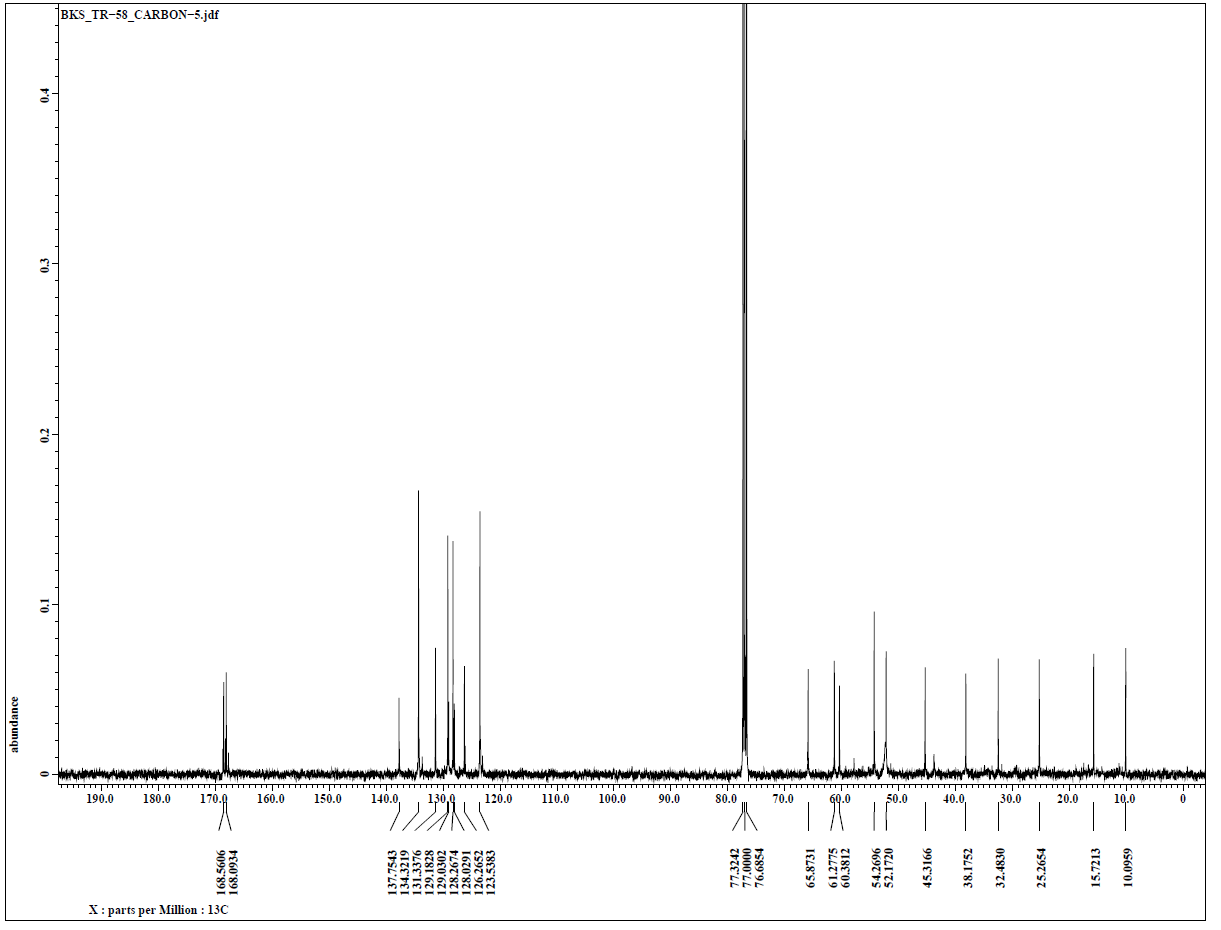

Supplement: S16 Fig — (TIF) [file pone.0139347.s016.tif]

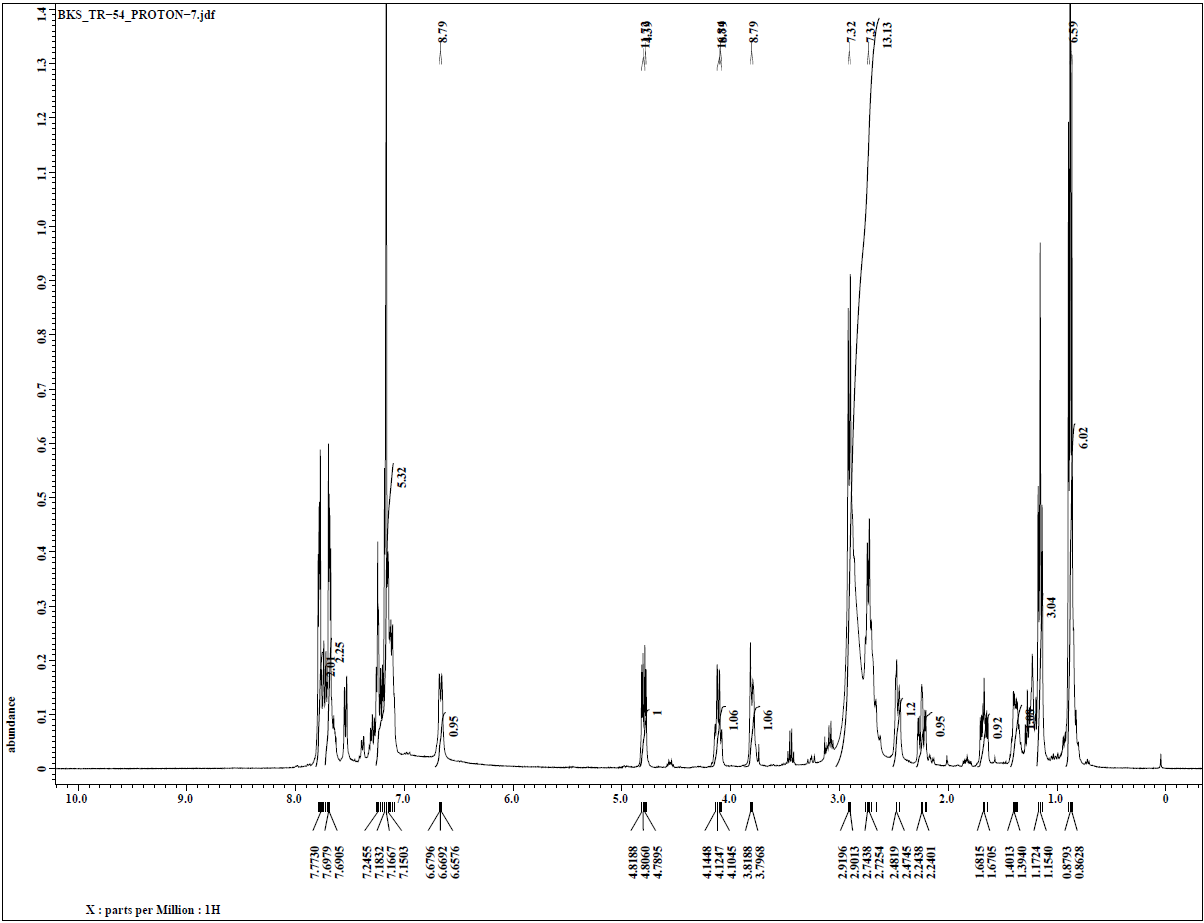

Supplement: S17 Fig — (TIF) [file pone.0139347.s017.tif]

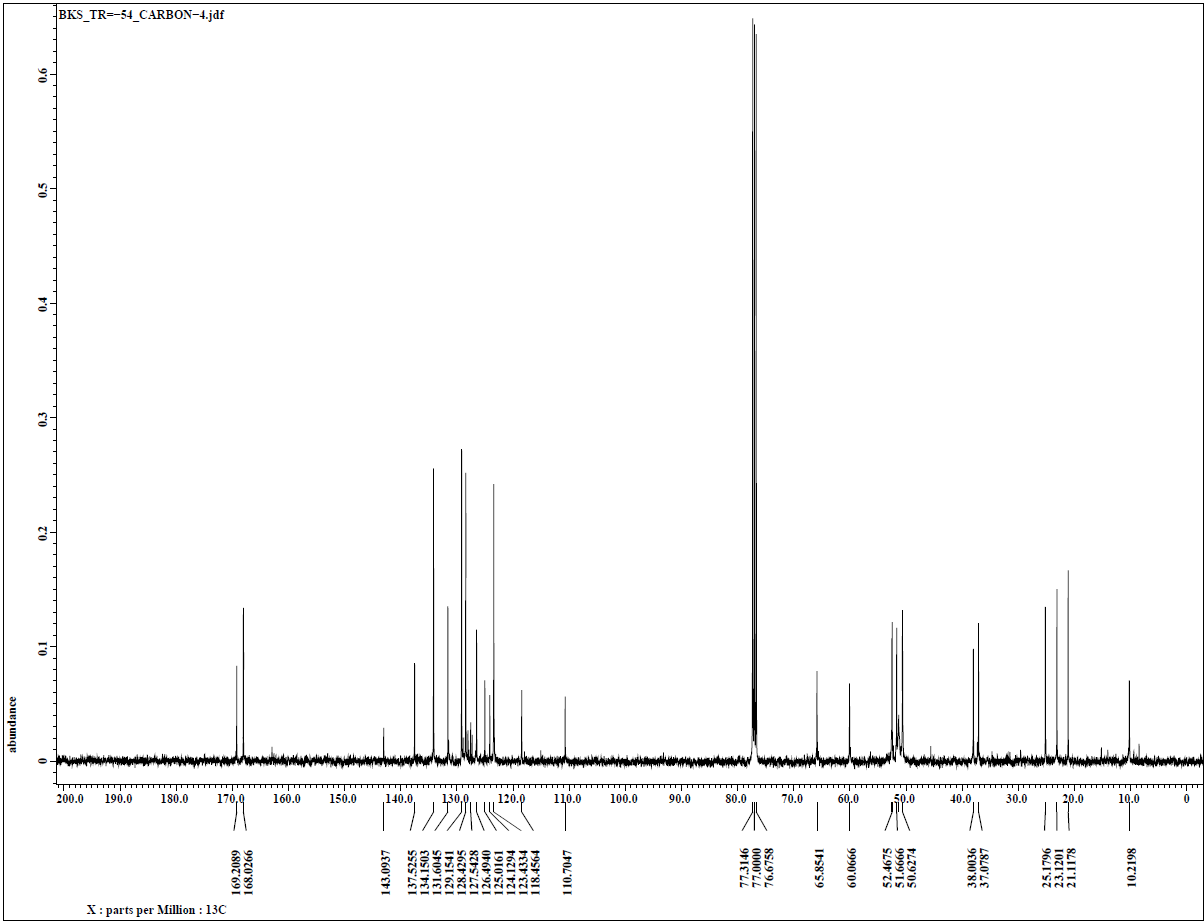

Supplement: S18 Fig — (TIF) [file pone.0139347.s018.tif]

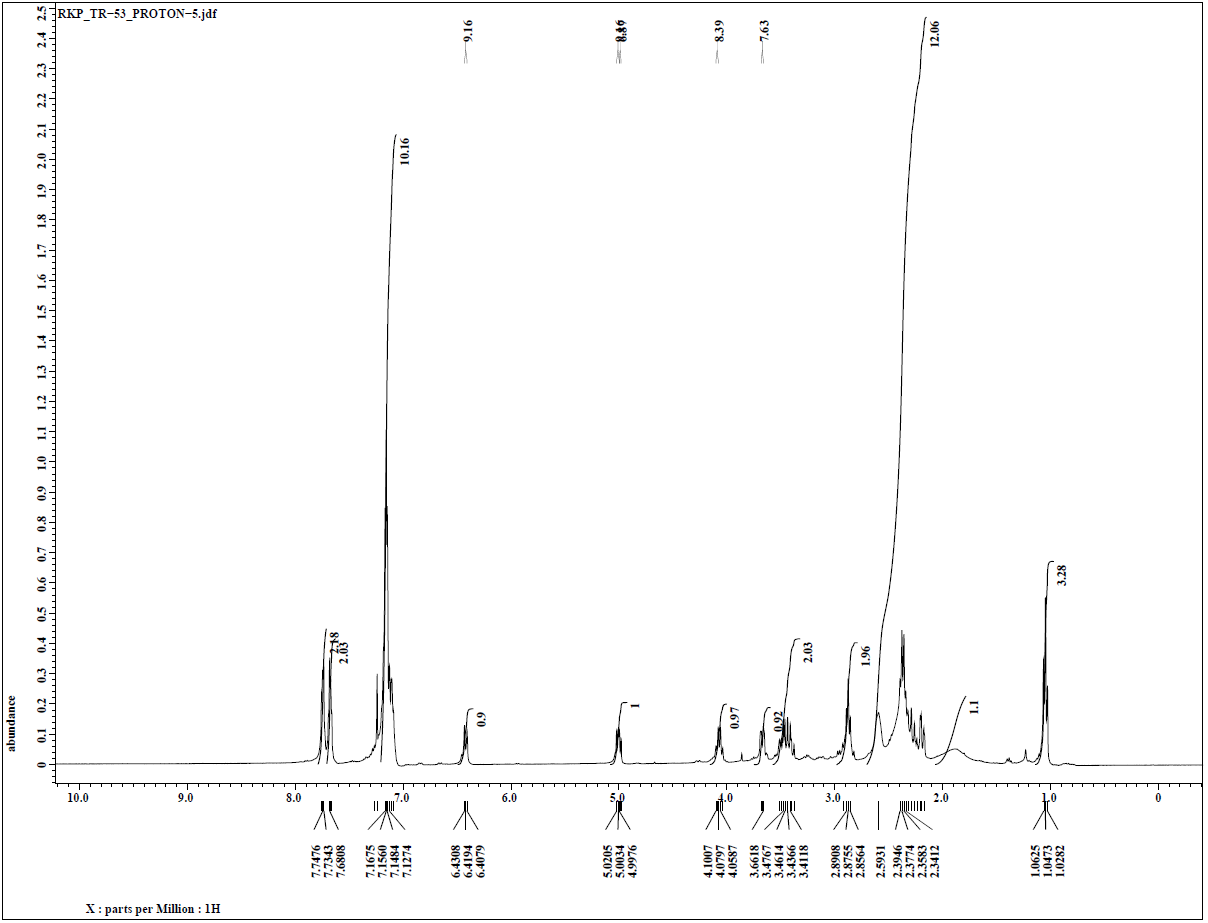

Supplement: S19 Fig — (TIF) [file pone.0139347.s019.tif]

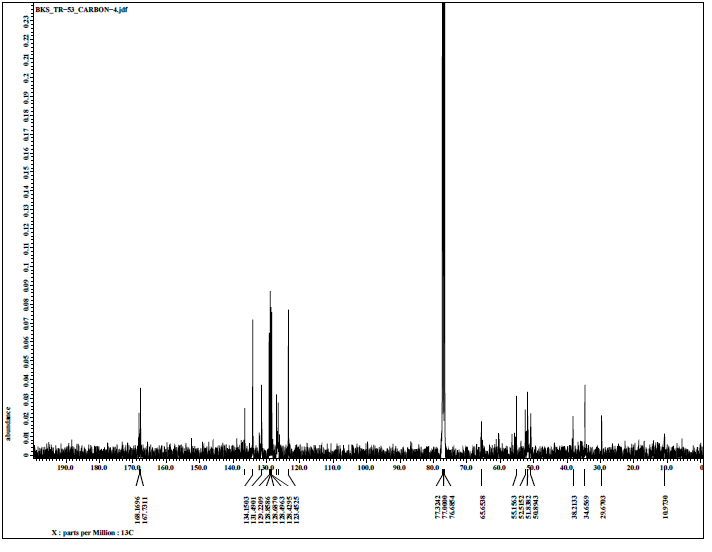

Supplement: S20 Fig — (TIF) [file pone.0139347.s020.tif]

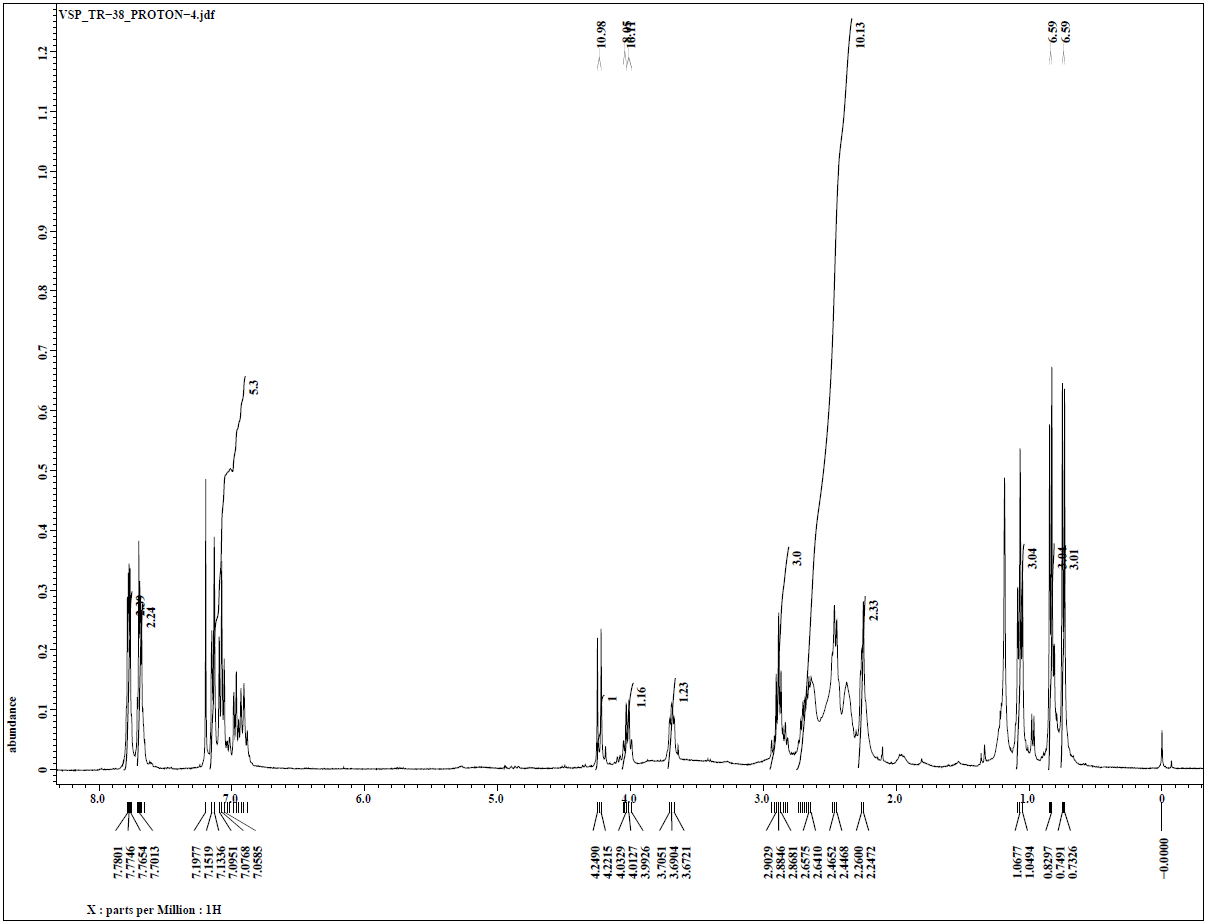

Supplement: S21 Fig — (TIF) [file pone.0139347.s021.tif]

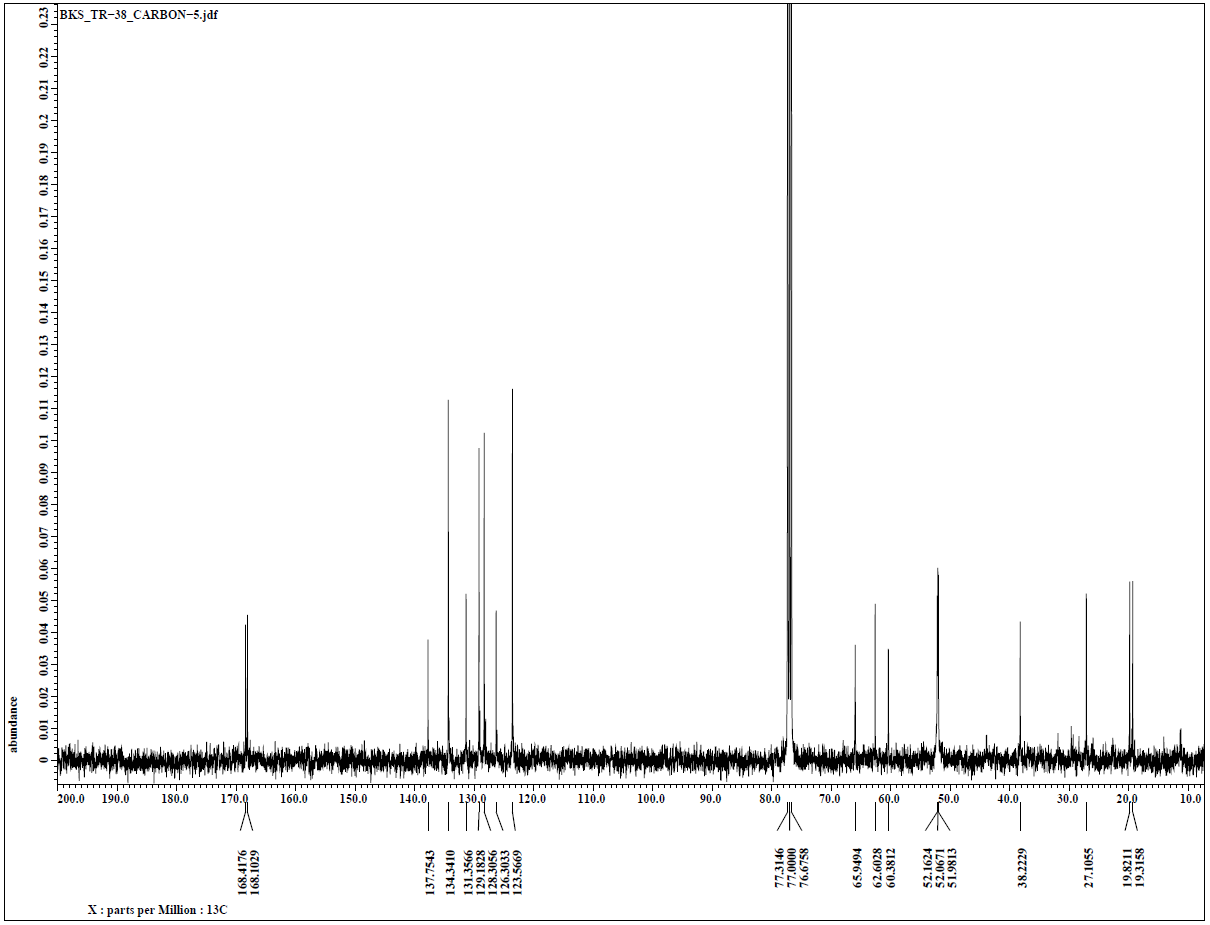

Supplement: S22 Fig — (TIF) [file pone.0139347.s022.tif]

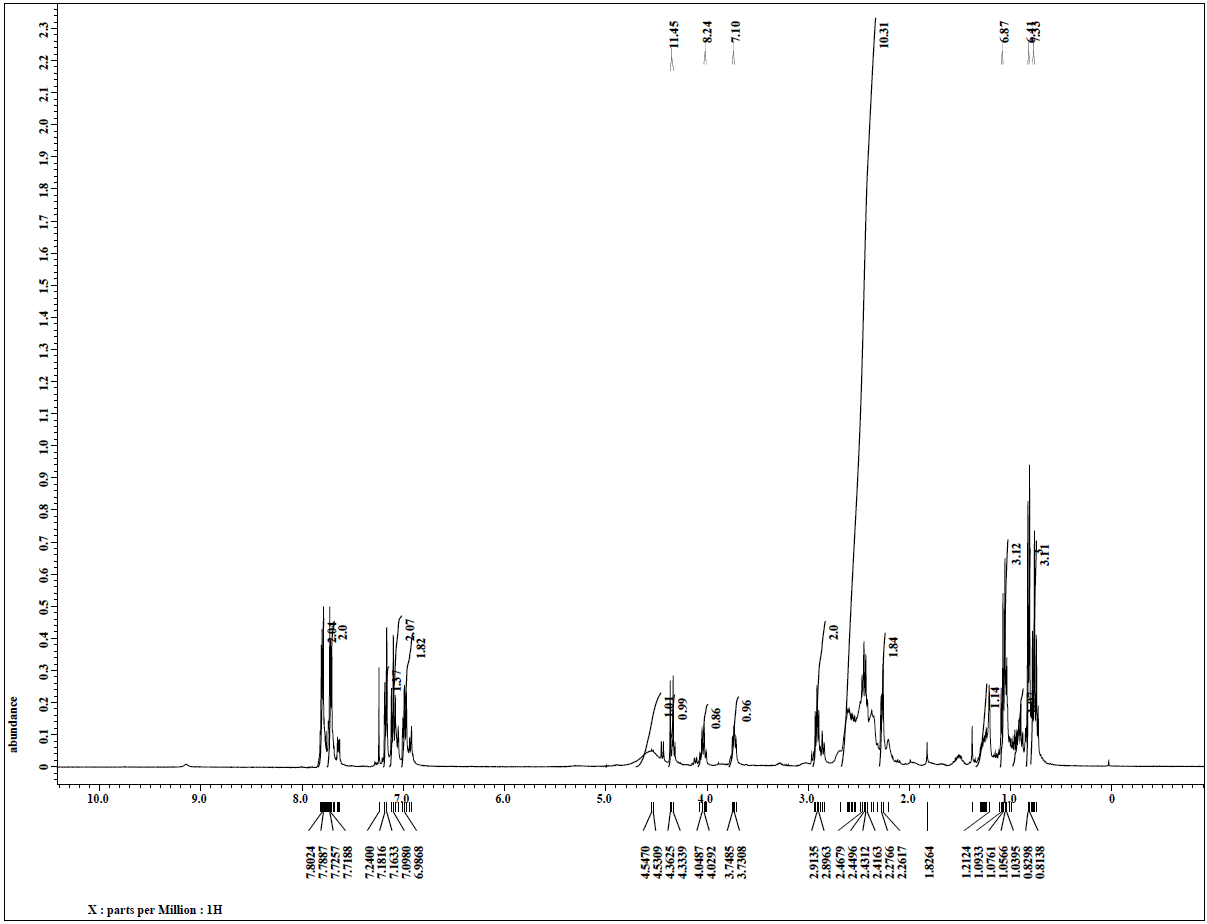

Supplement: S23 Fig — (TIF) [file pone.0139347.s023.tif]

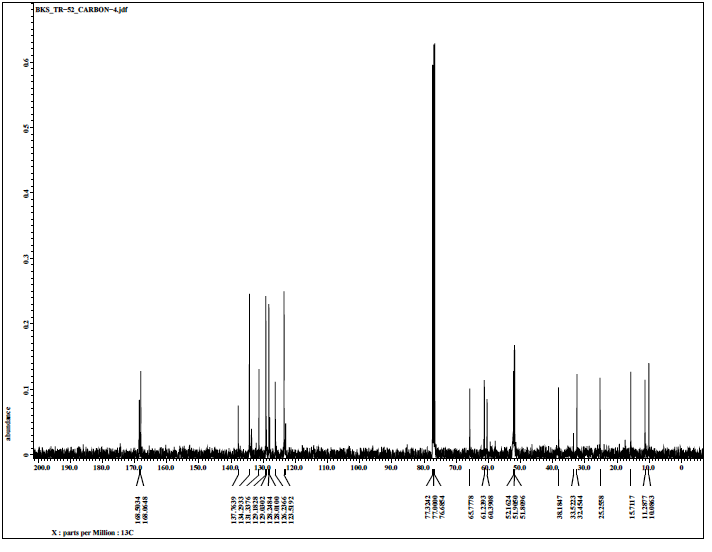

Supplement: S24 Fig — (TIF) [file pone.0139347.s024.tif]

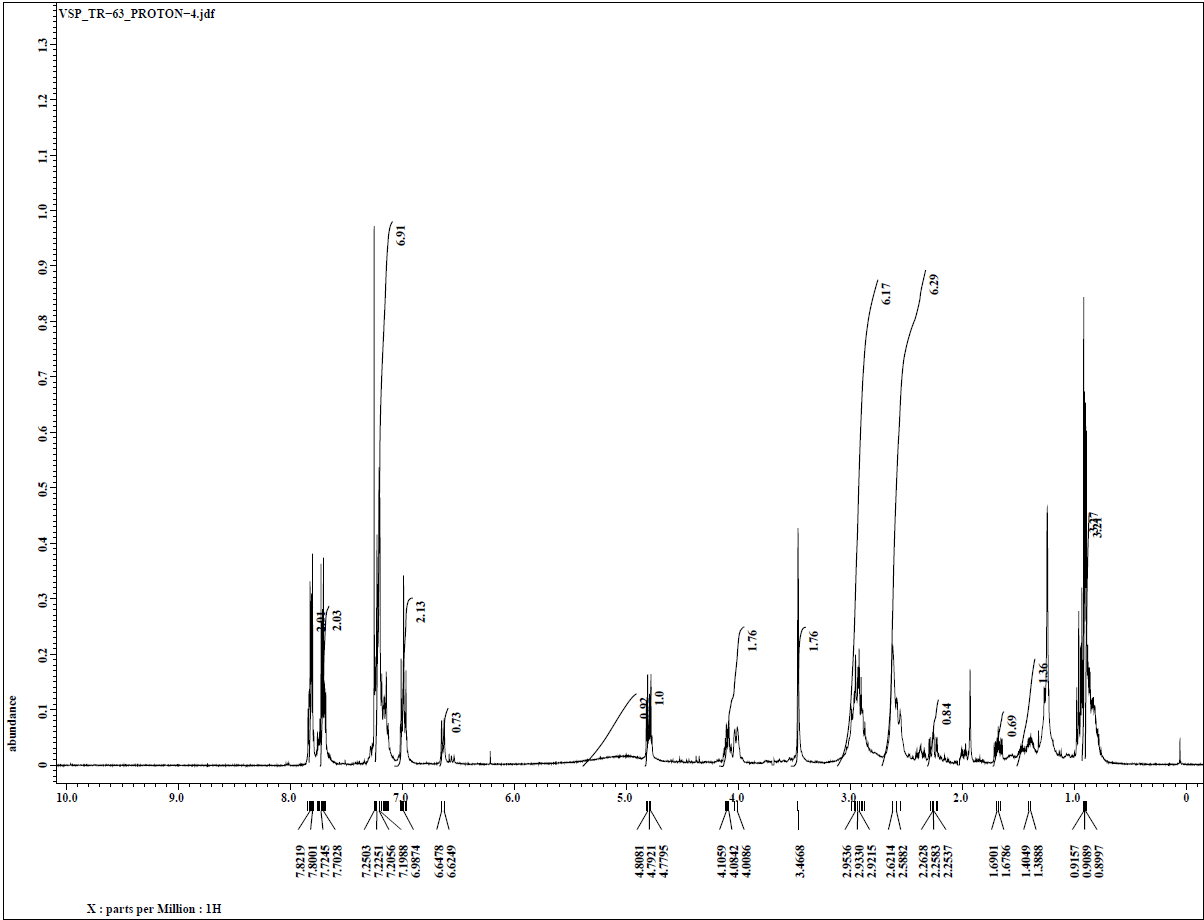

Supplement: S25 Fig — (TIF) [file pone.0139347.s025.tif]

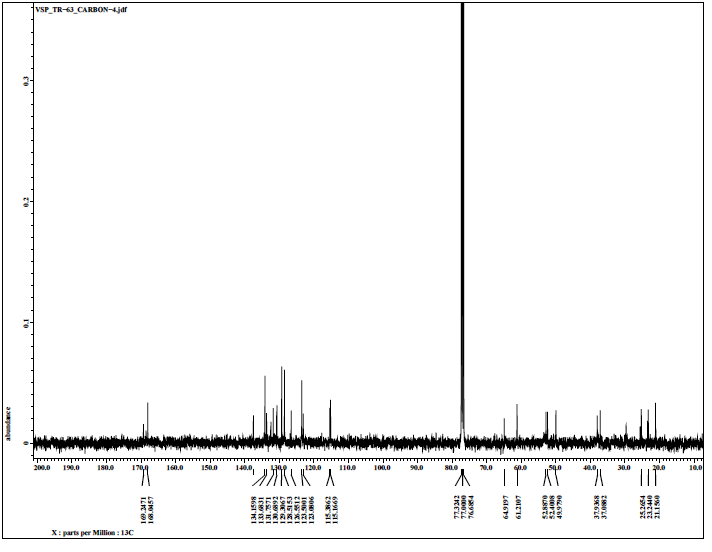

Supplement: S26 Fig — (TIF) [file pone.0139347.s026.tif]

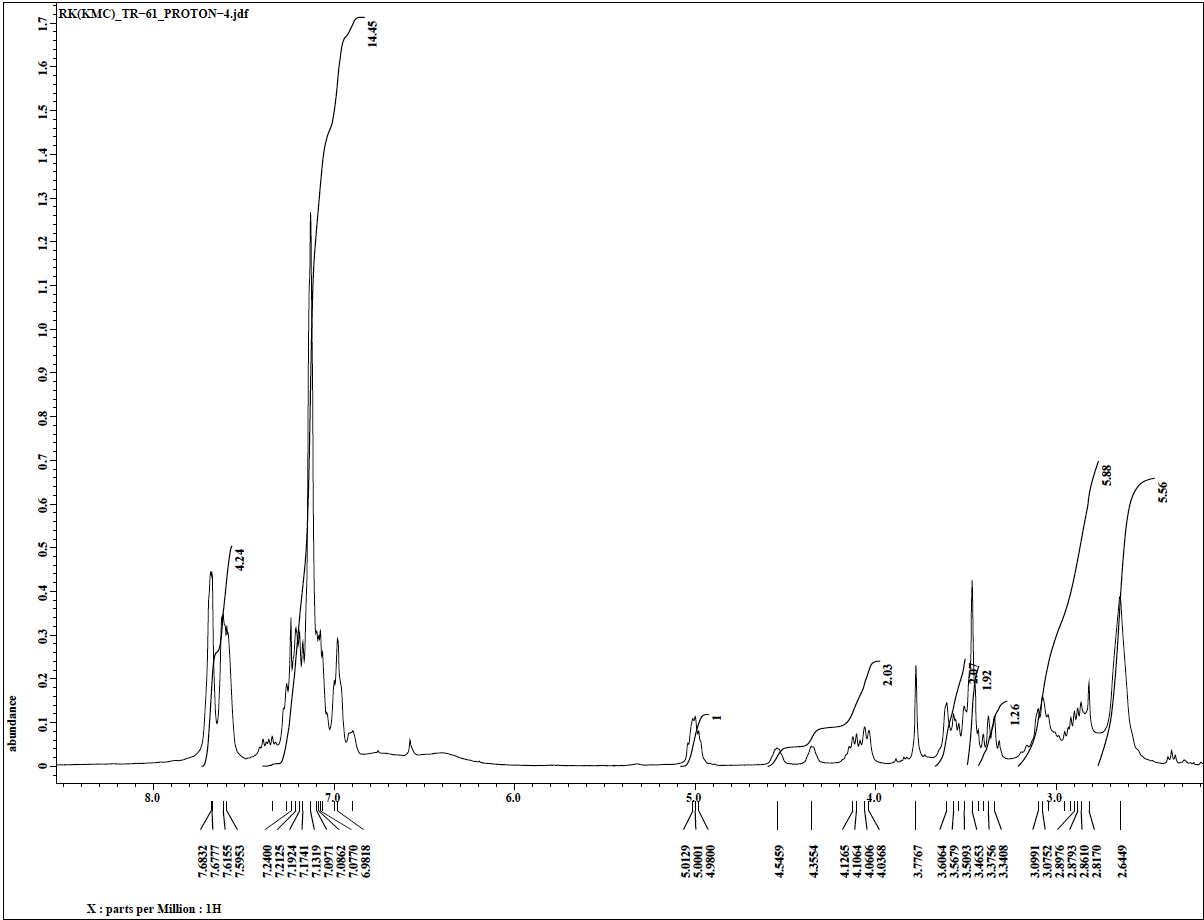

Supplement: S27 Fig — (TIF) [file pone.0139347.s027.tif]

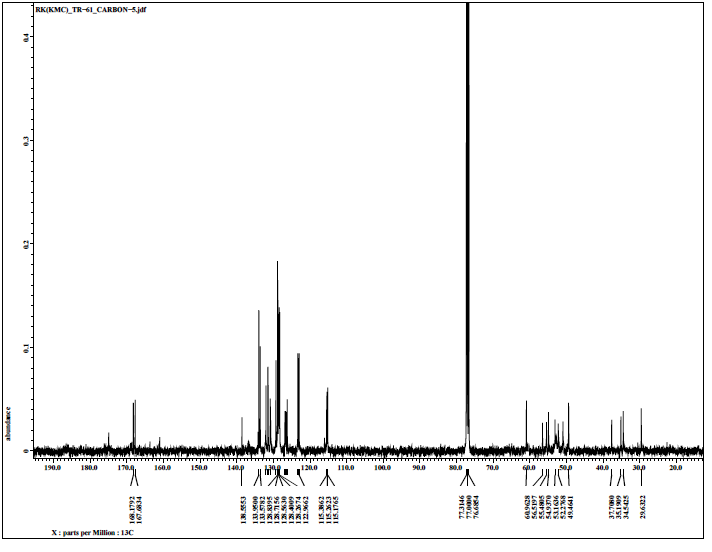

Supplement: S28 Fig — (TIF) [file pone.0139347.s028.tif]

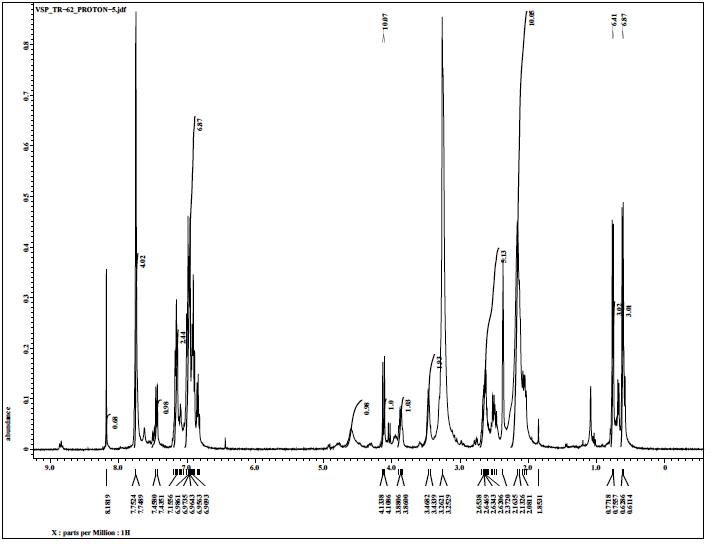

Supplement: S29 Fig — (TIF) [file pone.0139347.s029.tif]

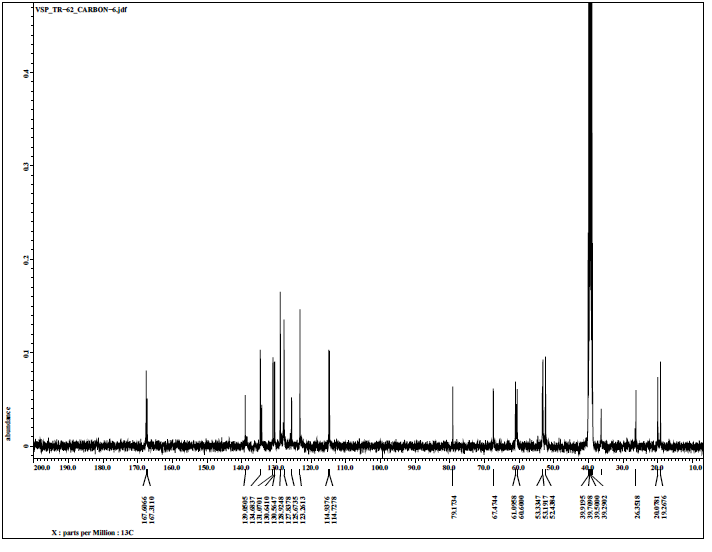

Supplement: S30 Fig — (TIF) [file pone.0139347.s030.tif]

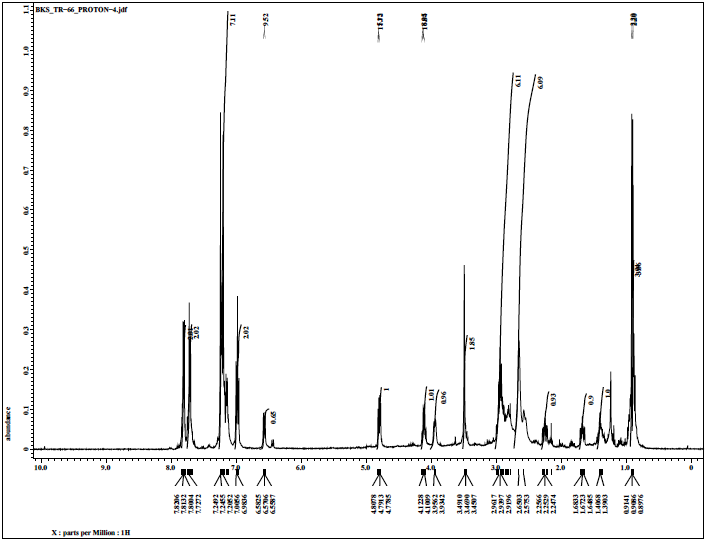

Supplement: S31 Fig — (TIF) [file pone.0139347.s031.tif]

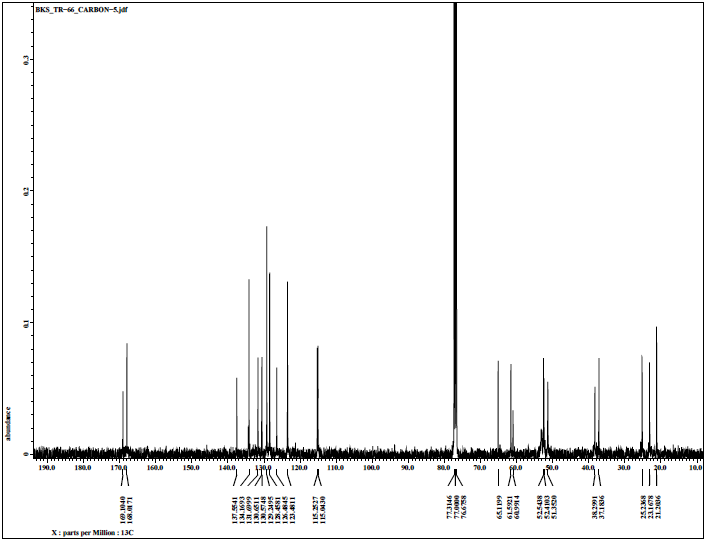

Supplement: S32 Fig — (TIF) [file pone.0139347.s032.tif]

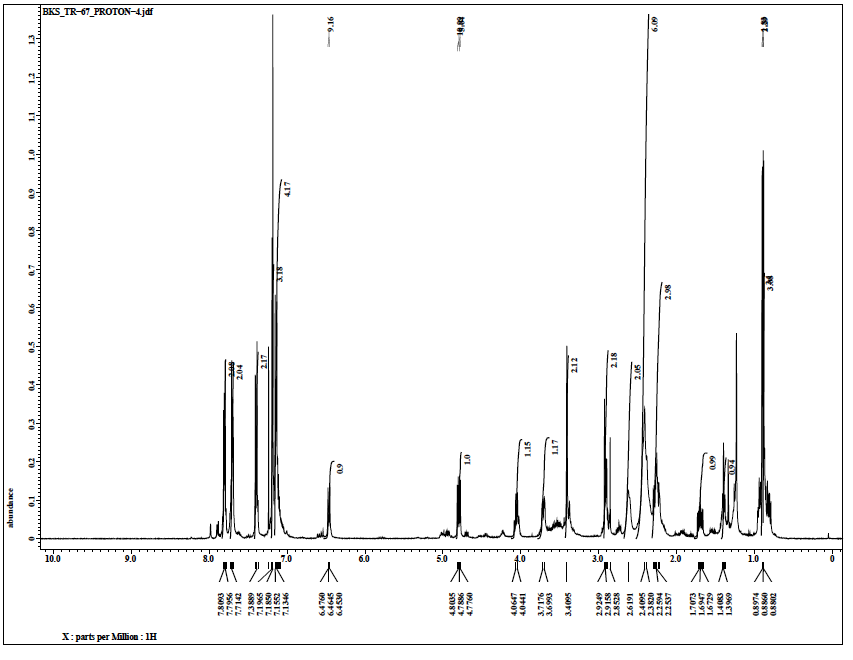

Supplement: S33 Fig — (TIF) [file pone.0139347.s033.tif]

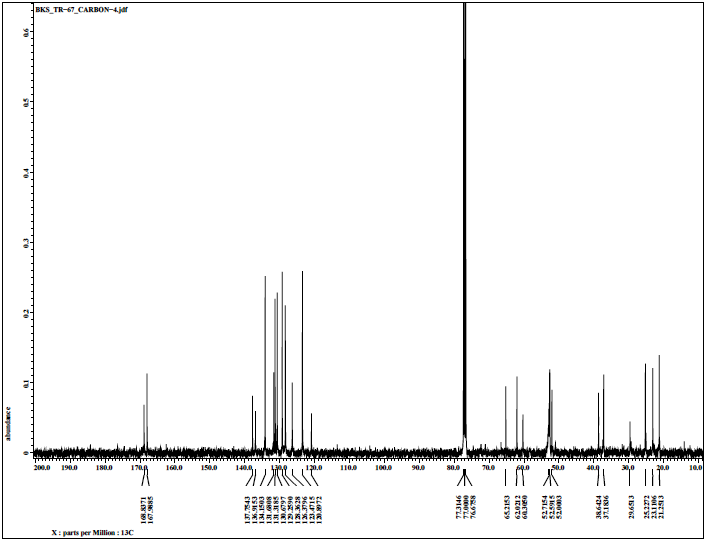

Supplement: S34 Fig — (TIF) [file pone.0139347.s034.tif]

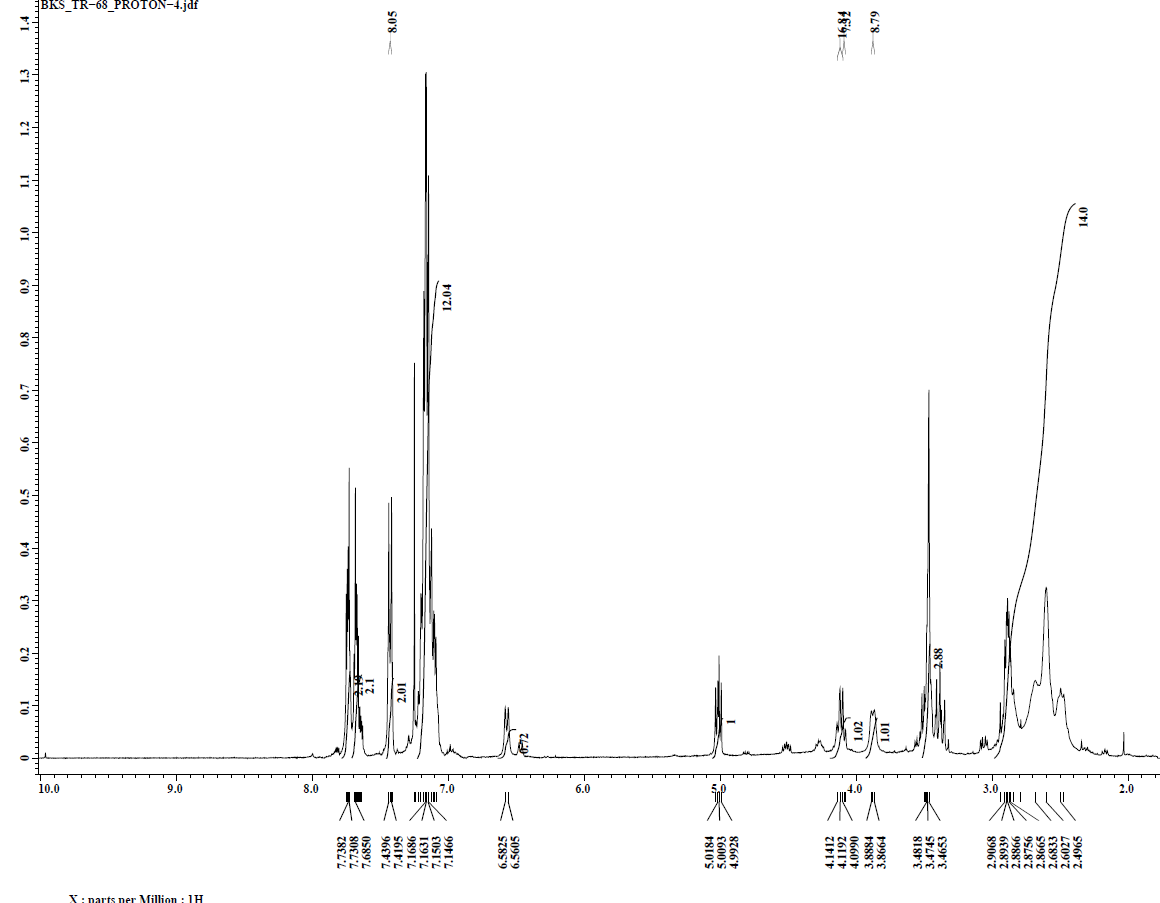

Supplement: S35 Fig — (TIF) [file pone.0139347.s035.tif]

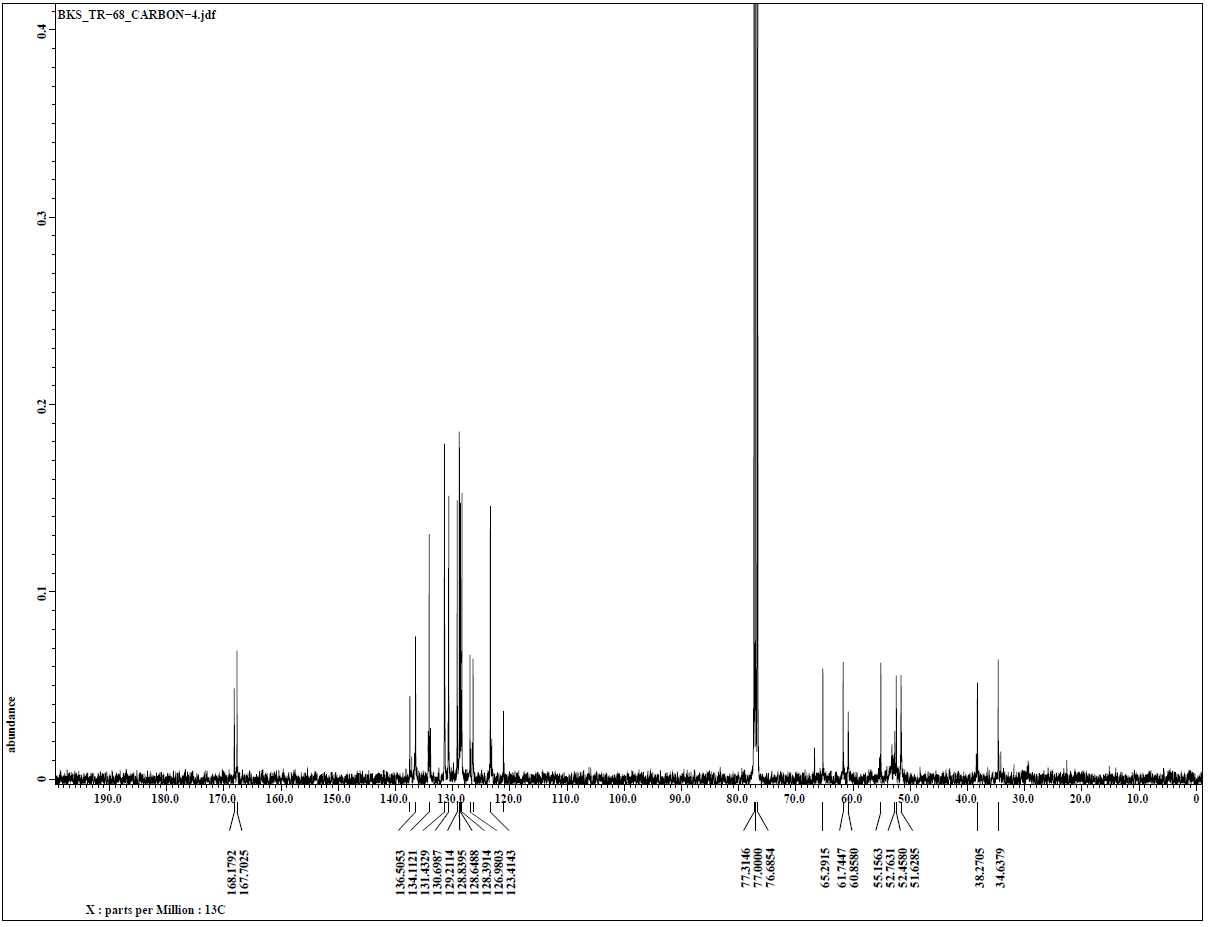

Supplement: S36 Fig — (TIF) [file pone.0139347.s036.tif]

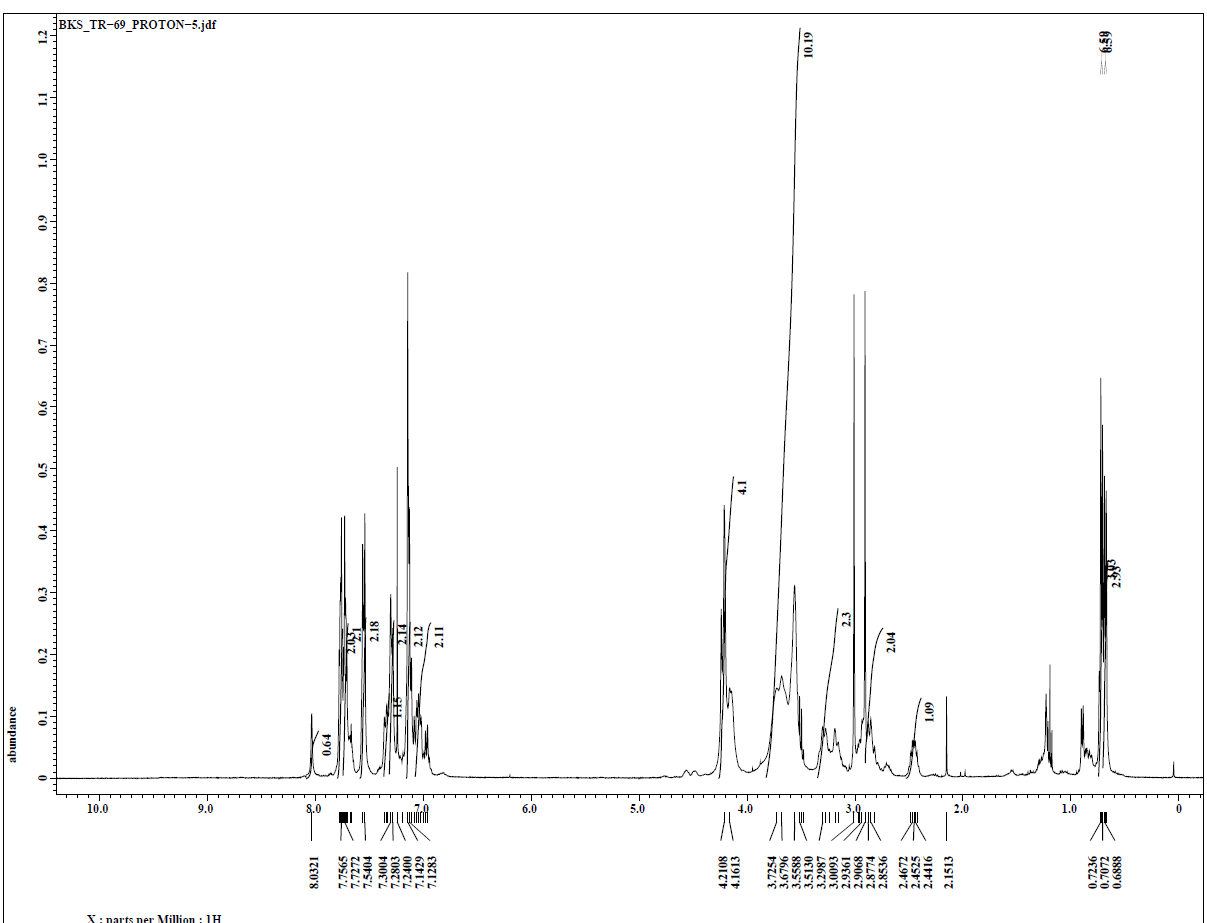

Supplement: S37 Fig — (TIF) [file pone.0139347.s037.tif]

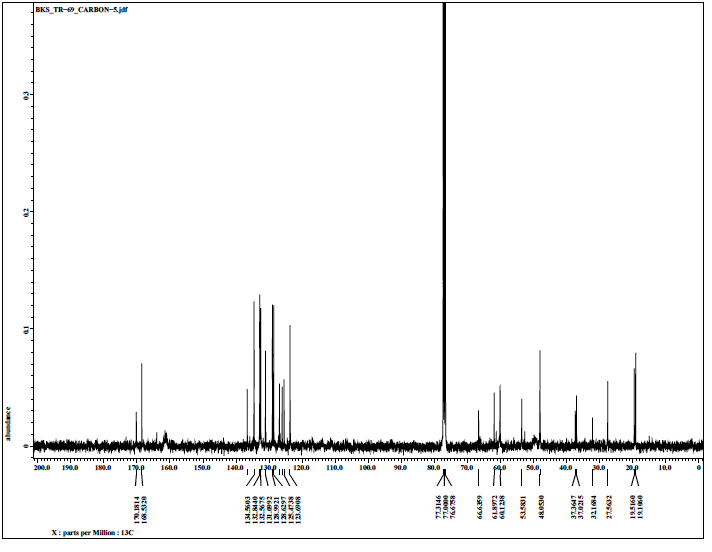

Supplement: S38 Fig — (TIF) [file pone.0139347.s038.tif]

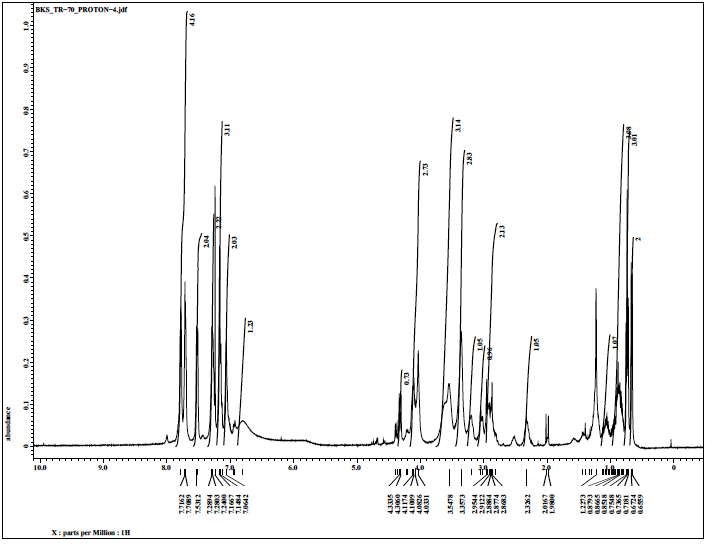

Supplement: S39 Fig — (TIF) [file pone.0139347.s039.tif]

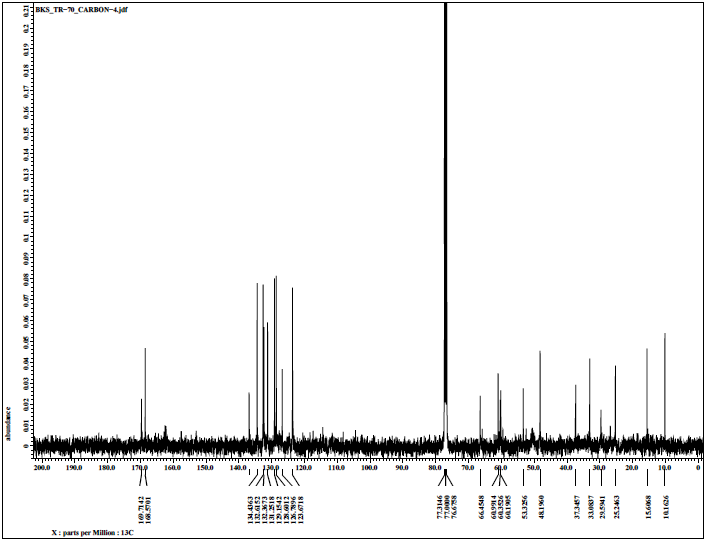

Supplement: S40 Fig — (TIF) [file pone.0139347.s040.tif]

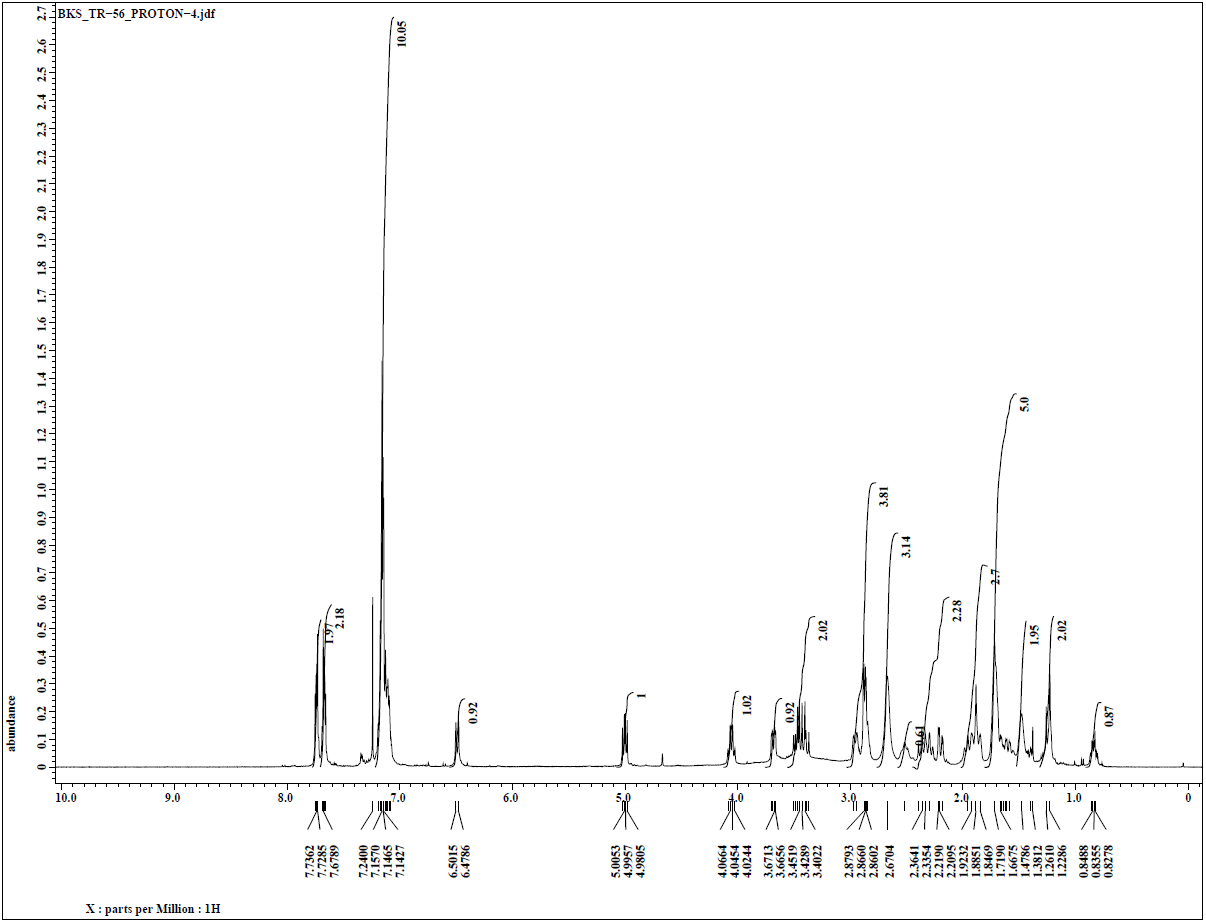

Supplement: S41 Fig — (TIF) [file pone.0139347.s041.tif]

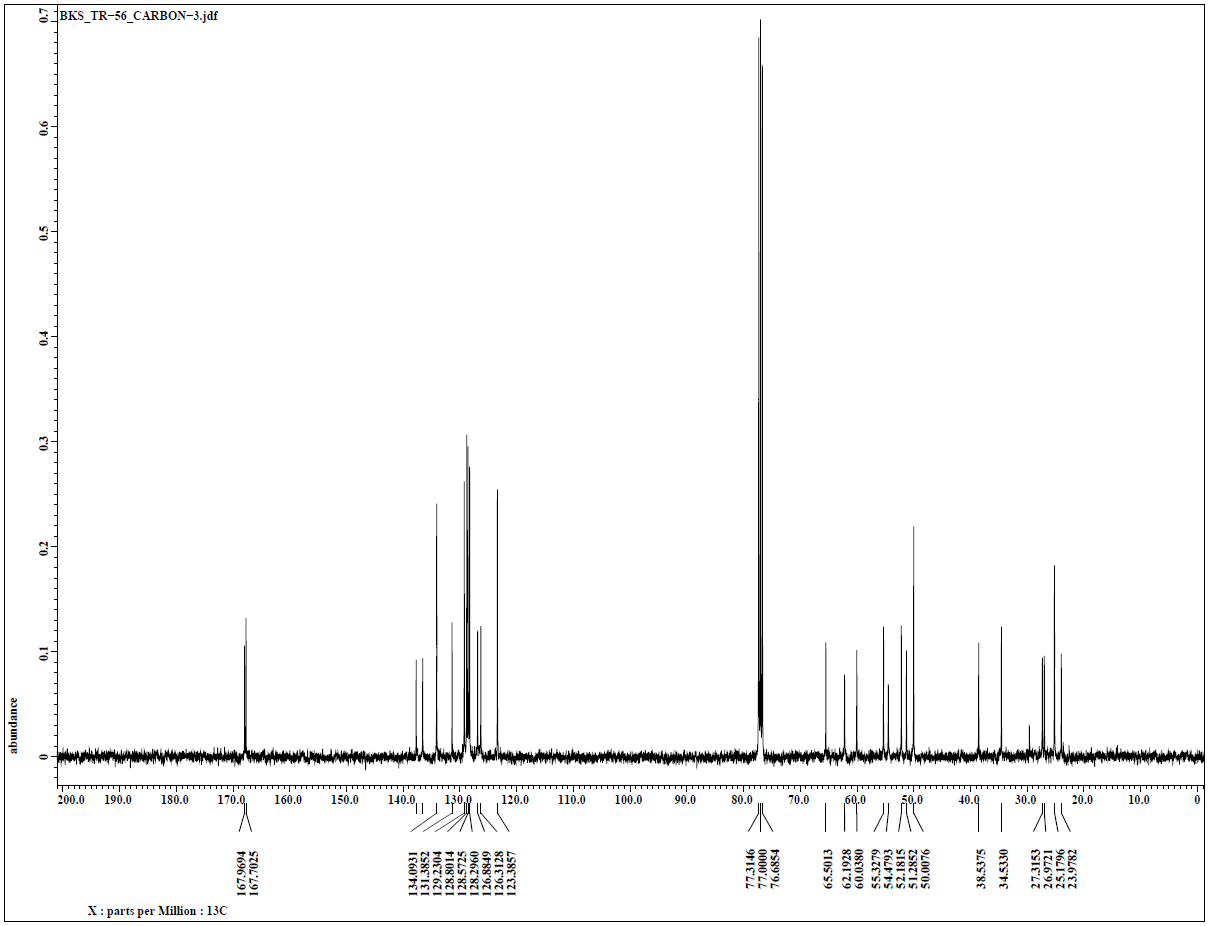

Supplement: S42 Fig — (TIF) [file pone.0139347.s042.tif]

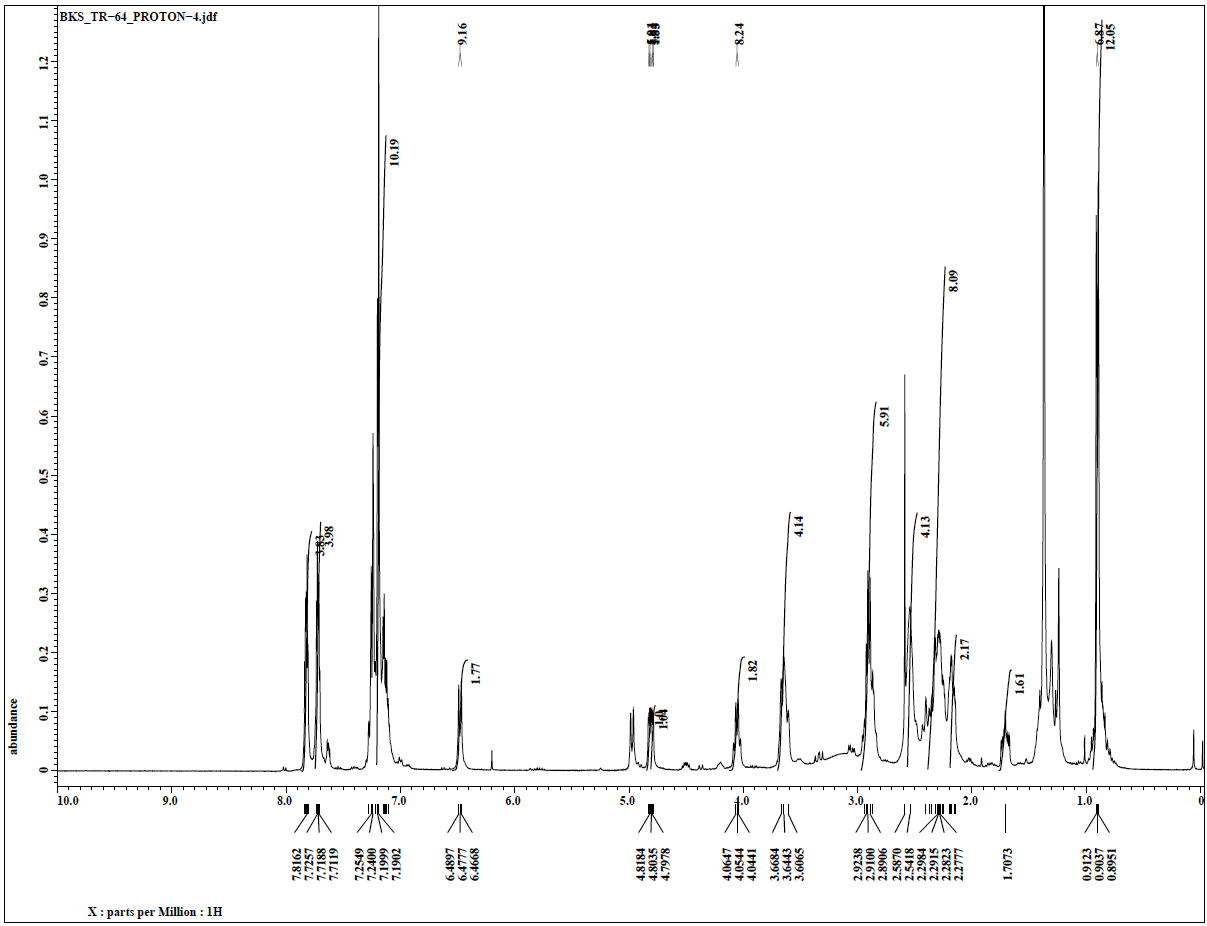

Supplement: S43 Fig — (TIF) [file pone.0139347.s043.tif]

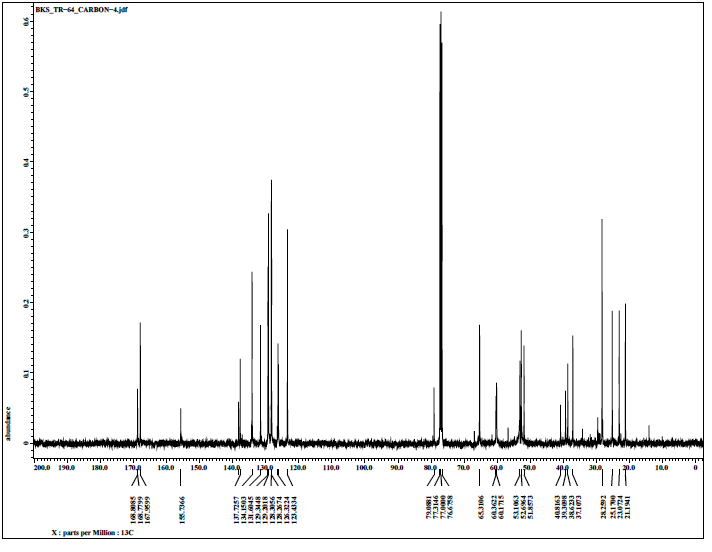

Supplement: S44 Fig — (TIF) [file pone.0139347.s044.tif]

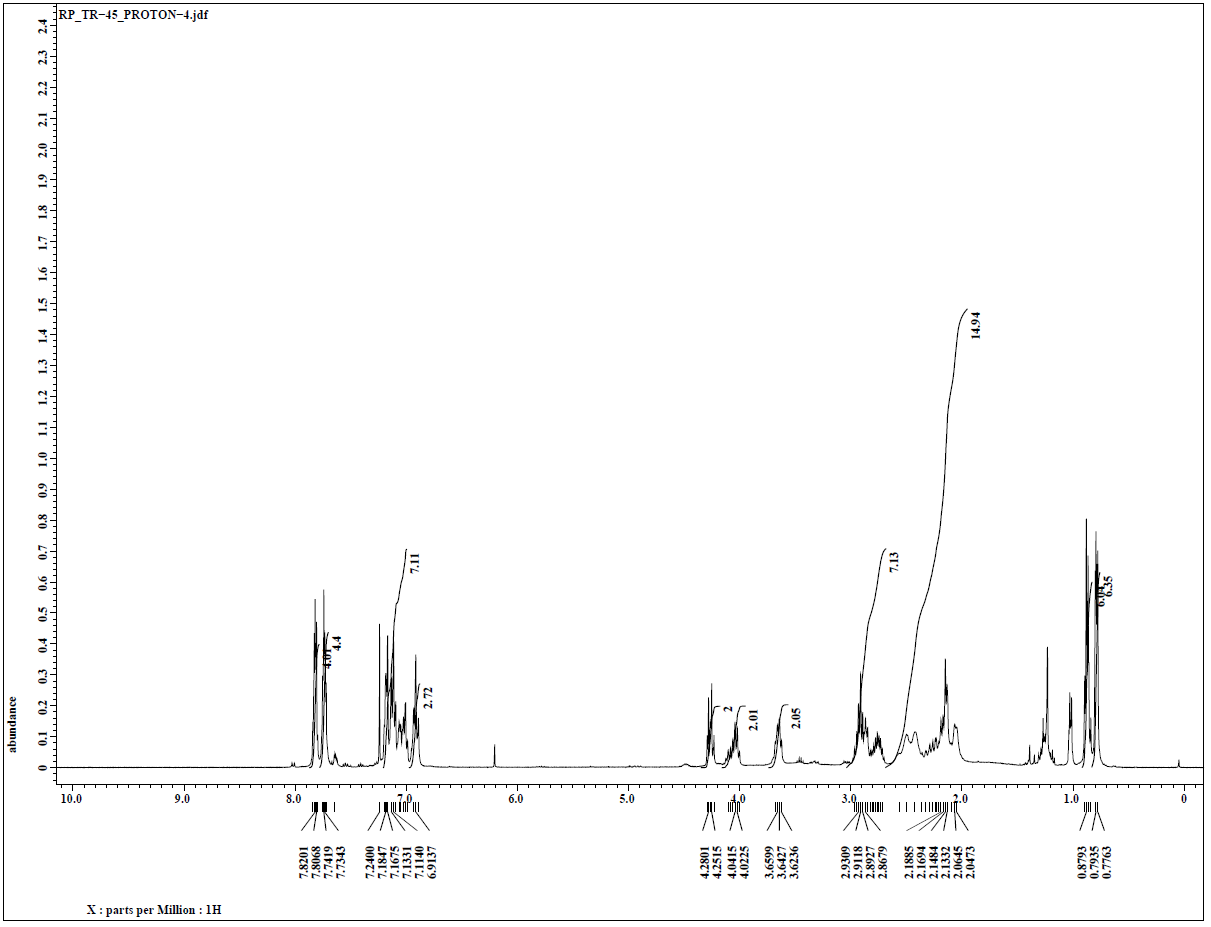

Supplement: S45 Fig — (TIF) [file pone.0139347.s045.tif]

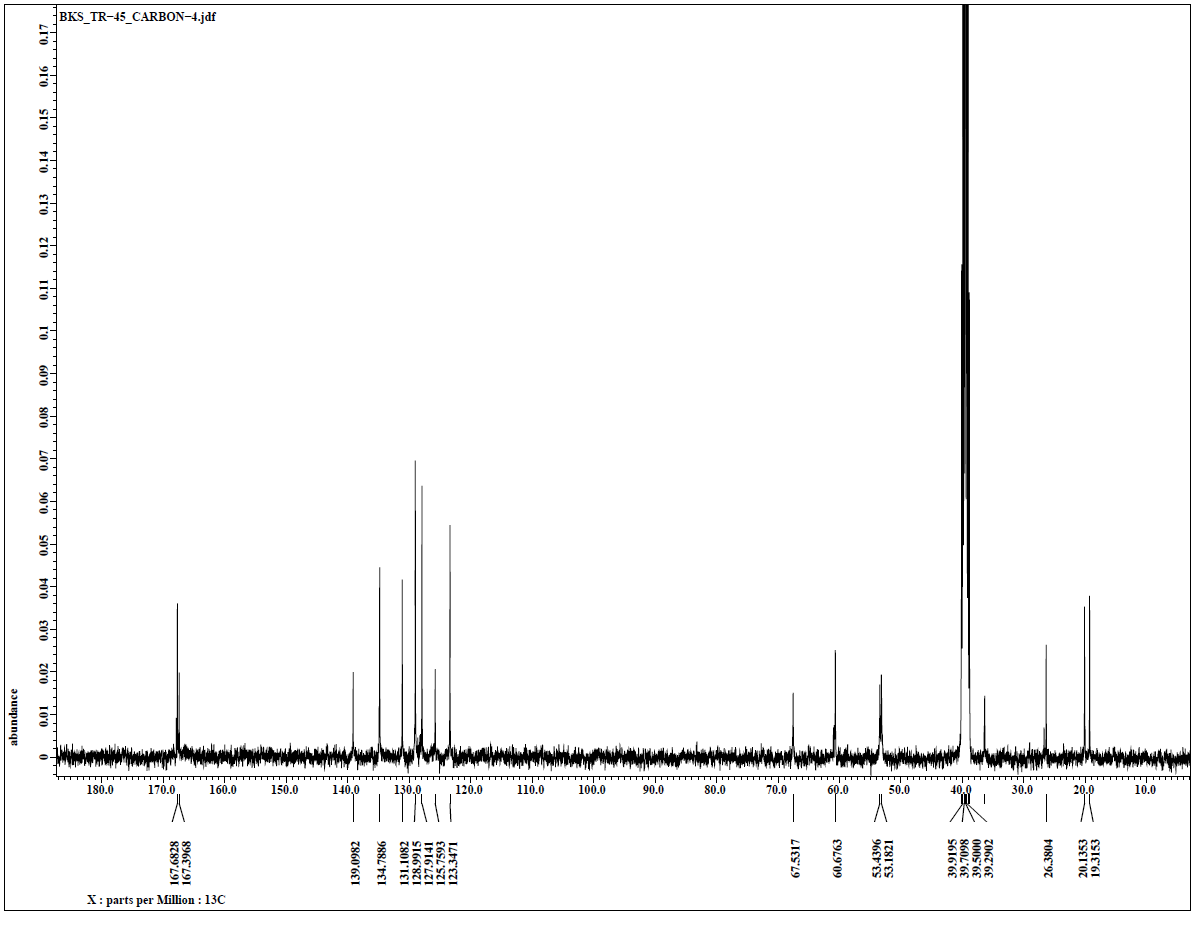

Supplement: S46 Fig — (TIF) [file pone.0139347.s046.tif]

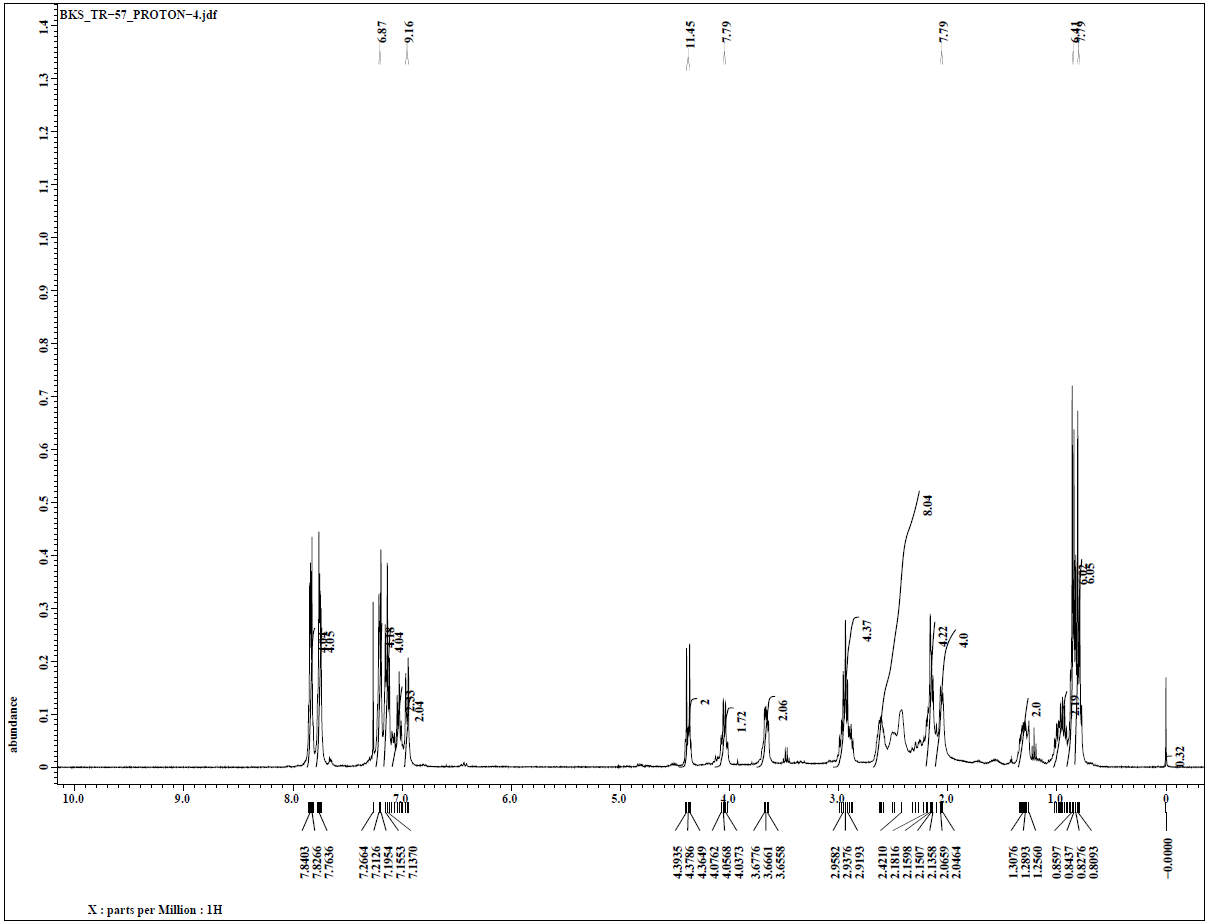

Supplement: S47 Fig — (TIF) [file pone.0139347.s047.tif]

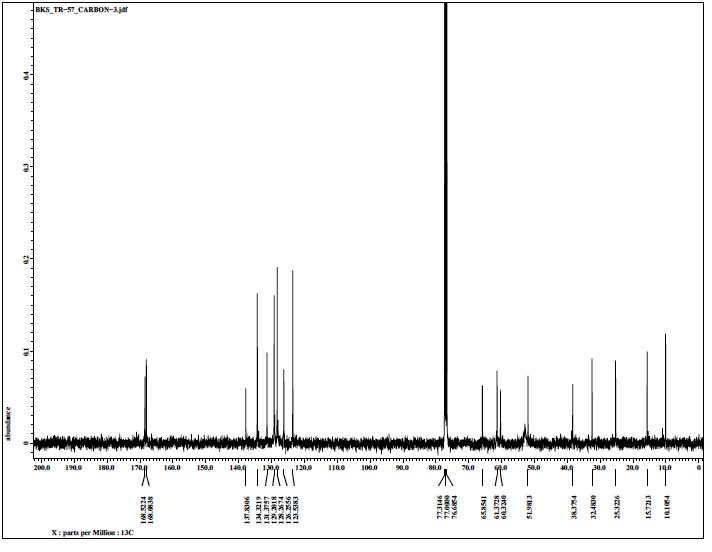

Supplement: S48 Fig — (TIF) [file pone.0139347.s048.tif]

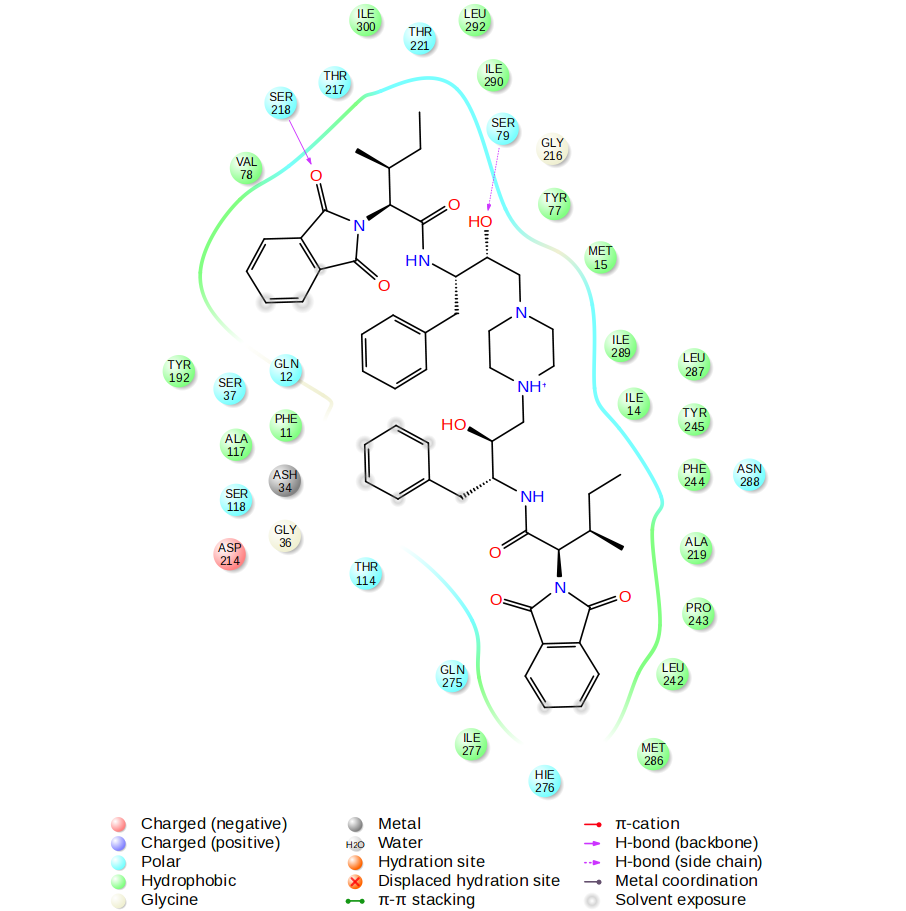

Supplement: S49 Fig — (TIF) [file pone.0139347.s049.tif]

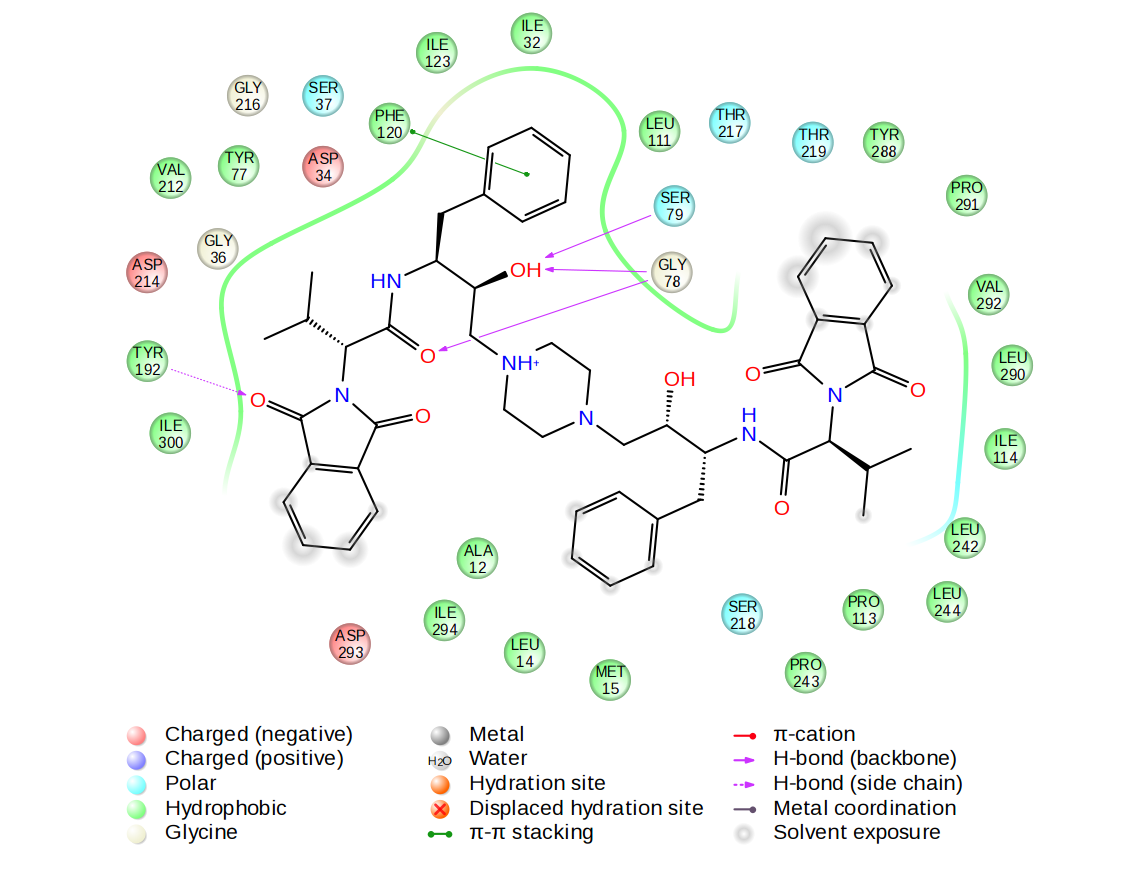

Supplement: S50 Fig — (TIF) [file pone.0139347.s050.tif]
