# Supplementary material for: Hydroxyethylamine Based Phthalimides as New Class of Plasmepsin Hits: Design, Synthesis and Antimalarial Evaluation
Source: PLoS One. 2015 Oct 26;10(10):e0139347. doi: 10.1371/journal.pone.0139347 (PMC4621027; doi:10.1371/journal.pone.0139347)
Supplement: S1 Table — (DOCX) [file pone.0139347.s051.docx]

Table S1. Physicochemical Properties of ptent compounds (**6r**, **6u**, **6s**, **6t**, **6p** and **5e**).

| Ligands | **6r** | **6u** | **6s** | **6t** | **6p** | **5e** |
| --- | --- | --- | --- | --- | --- | --- |
| MW | 899.09 | 899.09 | 967.13 | 871.04 | 484.39 | 431.61 |
| donorHB | 4 | 4 | 4 | 4 | 0 | 2 |
| accptHB | 18.4 | 18.4 | 18.4 | 18.4 | 8 | 8.2 |
| Rule Of Five | 3 | 3 | 3 | 2 | 0 | 0 |
| QPlogS | -7.22 | -7.06 | -6.65 | -6.33 | -3.69 | -3.26 |
| SASA | 1394.77 | 1376.13 | 1375.50 | 1333.01 | 739.15 | 757.61 |
| FOSA | 548.65 | 534.42 | 256.06 | 545.67 | 263.51 | 532.98 |
| FISA | 214.11 | 190.26 | 170.90 | 184.38 | 84.02 | 76.6 |
| HumanOralAbsorption | 1 | 1 | 1 | 1 | 3 | 3 |
| PSA | 212.46 | 208.82 | 207.85 | 208.85 | 80.84 | 66.80 |
| QPlogPoct | 48.48 | 49.20 | 51.43 | 47.42 | 20.65 | 23.01 |
| QPlogPw | 29.74 | 29.31 | 32.11 | 30.00 | 12.46 | 12.31 |

MW: Molecular weight of the molecule, SASA: Total solvent accessible surface area, FOSA: Hydrophobic component of the SASA (saturated carbon and attached hydrogen), FISA: Hydrophilic component of the SASA (SASA on N, O, and H on heteroatoms), Human Oral Absorption, PSA: Van der Waals surface area of polar nitrogen and oxygen atoms. QPlogpoct, QPlogPw and QPlogS were predicted partition coefficient of octanol/gas, water/gas, and aqueous solubility, respectively.
