# Supplementary material for: Hydroxyethylamine Based Phthalimides as New Class of Plasmepsin Hits: Design, Synthesis and Antimalarial Evaluation
Source: PLoS One. 2015 Oct 26;10(10):e0139347. doi: 10.1371/journal.pone.0139347 (PMC4621027; doi:10.1371/journal.pone.0139347)
Supplement: S2 Table — (DOCX) [file pone.0139347.s052.docx]

Table S2. The XP GScore and binding free energy values of potent compounds docked to Plasmepsin-2 (PDB ID: 2LF3).

| Ligands | XP GScores^(kcal/mol)^ | Binding Free Energy ^(kcal/mol)^ |
| --- | --- | --- |
| **6r** | -4.25 | -148.21 |
| **6u** | -5.47 | -138.54 |
| **6s** | -8.33 | -129.43 |
| **6t** | -7.30 | -125.58 |
| **5e** | -5.08 | -105.42 |
| **6p** | -5.39 | -71.45 |
