# Supplementary material for: Hydroxyethylamine Based Phthalimides as New Class of Plasmepsin Hits: Design, Synthesis and Antimalarial Evaluation
Source: PLoS One. 2015 Oct 26;10(10):e0139347. doi: 10.1371/journal.pone.0139347 (PMC4621027; doi:10.1371/journal.pone.0139347)
Supplement: S3 Table — (DOCX) [file pone.0139347.s053.docx]

Table S3. The XP GScore and binding free energy values of potent compounds docked to Plasmepsin 4 (PDB ID: 1LS5).

| Ligands | XP GScores^(kcal/mol)^ | Binding Free Energy^(kcal/mol)^ |
| --- | --- | --- |
| **6r** | -8.53 | -142.07 |
| **6u** | -8.35 | -138.96 |
| **6t** | -6.85 | -134.82 |
| **6s** | -7.27 | -125.76 |
| **5e** | -7.09 | -102.67 |
| **6p** | -2.97 | -69.91 |
