# Supplementary material for: Hydroxyethylamine Based Phthalimides as New Class of Plasmepsin Hits: Design, Synthesis and Antimalarial Evaluation
Source: PLoS One. 2015 Oct 26;10(10):e0139347. doi: 10.1371/journal.pone.0139347 (PMC4621027; doi:10.1371/journal.pone.0139347)
Supplement: S1 Text — (DOCX) [file pone.0139347.s054.docx]

**S1 Text_General consideration**.

If not otherwise specified, reagents and solvents were obtained from commercial suppliers and were used without further purification. Homogeneity/purity of all the products was assayed by thin-layer chromatography (TLC) on alumina-coated plates (Merck). Product samples in Chloroform (CHCl_3_) were loaded on TLC plates and developed in Ethyl acetate/Petroleum ether (1:1, v/v). When slight impurities were detected by iodine vapour/UV light visualization, compounds were further purified by chromatography on silica gel columns (100-200 mesh size, CDH). Melting points were determined on Melting point machine M-560 (Buchi). Infrared (IR) spectra were recorded in KBr medium using a Perkin-Elmer Fourier Transform-IR spectrometer, whereas ^1^H and ^13^C nuclear magnetic resonance (NMR) spectra were recorded in CDCl_3_ medium on a JNM ECX-400P (JEOL, USA) spectrometer with tetramethylsilane (TMS) as internal reference. Absorption frequencies (ν) are expressed in cm^−1^, chemical shifts in ppm (δ-scale) and coupling constants (*J*) in Hz. Splitting patterns are described as singlet (s), doublet (d), triplet (t), quartet (q) and multiplet (m). High-resolution EI-mass spectra were performed on MStation JMS-700 spectrometer (Co. JEOL, made in Japan). *tert*-Butyl((2R,3S)-3-hydroxy-4-(4-methylpiperazin-1-yl)-1-phenylbutan-2-yl)carbamate (**5a**); Di-*tert*-butyl((2R,2'R,3S,3'S)-piperazine-1,4-diylbis(3-hydroxy-1-phenylbutane-4,2-diyl))dicarbamate (**5f**); *N*,*N*'-((2R,2'R,3S,3'S)-piperazine-1,4-diylbis(3-hydroxy-1-phenylbutane-4,2-diyl))bis(2-(1,3-dioxoisoindolin-2-yl)-3 phenylpropanamide) (**6s**) were synthesised and characterized according to the literature procedure [S1].
