# Supplementary material for: Hydroxyethylamine Based Phthalimides as New Class of Plasmepsin Hits: Design, Synthesis and Antimalarial Evaluation
Source: PLoS One. 2015 Oct 26;10(10):e0139347. doi: 10.1371/journal.pone.0139347 (PMC4621027; doi:10.1371/journal.pone.0139347)
Supplement: S2 Text — (DOC) [file pone.0139347.s055.doc]

**S2 Text_Spectroscopic data of synthesised compounds (5b-6r, 6t and 6u).**

***tert*-Butyl((2*R*,3*S*)-4-(4-ethylpiperazin-1-yl)-3-hydroxy-1-phenylbutan-2-yl)carbamate** (**5b**): White solid. Yield: 87 %. mp: 89-91 ^o^C. IR (KBr) ν_max_ (cm^-1^): 3396 (OH), 2977, 2824, 1687 (CO), 1510, 1170, 1012, 701, 579; ^1^H NMR (400 MHz, CDCl_3_): 1.14 (3H, t, *J* = 5.13 Hz), 1.31 (9H, s), 2.53 (8H, m), 2.74 (3H, m), 2.93 (3H, m), 3.70 (2H, d, *J* = 8.79 Hz), 4.97 (1H, d, *J* = 9.52 Hz), 7.28 (5H, m) ppm. ^13^C NMR (100 MHz, CDCl_3_): 11.6, 28.0, 28.3, 39.3, 52.1, 52.5, 53.1, 60.4, 65.3, 79.1, 126.2, 128.3, 129.4, 138.2, 155.7 ppm. HRMS *m*/*z* calculated [M]^+^ for C_21_H_36_N_3_O_3_^+^: 377.2678; found: 377.2674.

***tert*-Butyl((2*R*,3*S*)-4-(4-(4-fluorobenzyl)piperazin-1-yl)-3-hydroxy-1-phenylbutan-2-yl)carbamate** (**5c**): White solid. Yield: 89 %. mp: 108-110 ^o^C. IR (KBr) ν_max_ (cm^-1^): 3431 (OH), 3350, 2938, 2805, 1687 (CO), 1510, 1161, 816, 520. ^1^H NMR (400 MHz, CDCl_3_): 1.35 (9H, s), 2.24 (1H, dd, *J* = 1.48, 11.72 Hz), 2.35-2.50 (7H, m), 2.60 (2H, m), 2.91 (3H, m), 3.42 (2H, s), 3.65 (2H, dd, *J* = 8.79, 15.38 Hz), 4.96 (1H, d, *J* = 10.25 Hz) 6.98 (2H, t, *J* = 8.79 Hz), 7.18-7.25 (7H, m) ppm. ^13^C NMR (100 MHz, CDCl_3_): 28.2, 39.3, 52.8, 53.1, 60.3, 62.0, 65.2, 79.1, 114.8, 115.0, 126.1, 128.3, 128.4, 129.3, 129.4, 130.4, 130.5, 138.2, 155.7, 160.7, 163.1 ppm. HRMS *m*/*z* Calculated [M+H]^+^ for C_26_H_37_FN_3_O_3_^+^: 458.2813; found 458.2809.

***tert*-Butyl((2*R*,3*S*)-4-(4-(4-bromobenzyl)piperazin-1-yl)-3-hydroxy-1-phenylbutan-2-yl)carbamate** (**5d**): White solid. Yield: 89%. mp: 111-114 ^o^C. IR (KBr) ν_max_ (cm^-1^): 3337 (OH), 2937, 2816, 1705 (CO), 1489, 1167, 1010, 755. ^1^H NMR (400 MHz, CDCl_3_): 1.35 (9H, s), 2.80-2.91 (12H, m), 3.12 (1H, m), 3.50 (2H, s), 3.75 (1H, m), 4.08 (1H, br s), 4.96 (1H, d, *J* = 10.40 Hz), 7.18 (3H, m), 7.21 (2H, d, *J* = 7.32 Hz), 7.26 (2H, d, *J* = 7.32 Hz), 7.43 (2H, d, *J* = 8.05 Hz) ppm. ^13^C NMR (100 MHz, CDCl_3_): 28.2, 31.5, 34.6, 39.1, 51.9, 53.5, 61.8, 65.1, 79.3, 121.0, 126.3, 128.3, 129.4, 130.7, 131.4, 138.0, 155.8 ppm. HRMS *m*/*z* Calculated [M+H]^+^ for C_26_H_37_BrN_3_O_3_^+^:518.2013; found 518.2005.

***tert*-Butyl((2*R*,3*S*)-4-([1,4'-bipiperidin]-1'-yl)-3-hydroxy-1-phenylbutan-2-yl)carbamate** (**5e**): White solid. Yield: 85 %. mp: 116-119 ^o^C. IR (KBr) ν_max_ (cm^-1^): 3383 (OH), 2933, 2799, 1700 (CO), 1518, 1367, 1247, 1174, 1111, 701; ^1^H NMR (400 MHz, CDCl_3_): 1.42 (9H, s), 1.59 (6H, m), 1.86 (4H, m), 2.16 (1H, m), 2.29 (2H, m), 2.42 (1H, t, *J* = 11.8 Hz), 2.49 (4H, br s), 2.92, (4H, m), 3.60 (3H, m), 5.00 (1H, d, *J* = 9.62 Hz), 7.27 (5H, m) ppm. ^13^C NMR (100 MHz, CDCl_3_): 24.5, 26.0, 27.8, 28.3, 39.4, 50.2, 51.4, 53.1, 55.1, 60.2, 62.4, 65.3, 79.0, 126.1, 128.2, 129.4, 138.3, 155.8 ppm. HRMS *m/z* Calculated [M+H]^+^ for C_25_H_42_N_3_O_3_^+^: 432.3221; found 432.3215.

**(*S*)-2-(1,3-Dioxoisoindolin-2-yl)-*N*-((2*R*,3*S*)-3-hydroxy-4-(4-methylpiperazin-1-yl)-1-phenylbutan-2-yl)-4-methylpentanamide** (**6a**): White solid. Yield: 46 %. mp: 141-143 ^o^C. IR (KBr) ν_max_ (cm^-1^): 3312 (OH), 2958, 2457, 1714 (CO), 1671 (CO), 1385, 1078, 754; ^1^H NMR (400 MHz, CDCl_3_): 0.89 (6H, d, *J* = 5.86 Hz), 1.39 (1H, m), 1.69 (1H, m), 2.24 (1H, m), 2.29 (1H, m), 2.32 (3H, s), 2.45-2.60 (8H, m), 2.72 (2H, d, *J* = 7.32 Hz), 2.91 (2H, d, *J* = 7.32 Hz), 3.75 (1H, dd, *J* = 2.93, 10.25 Hz), 4.11 (1H, q, *J* = 8.05 Hz), 4.81 (1H, dd, *J* = 4.39, 11.72 Hz), 6.50 (1H, d, *J* = 8.79 Hz), 7.19 (5H, m), 7.71 (2H, m), 7.81 (2H, m) ppm. ^13^C NMR (100 MHz, CDCl_3_): 21.2, 23.1, 25.2, 37.1, 38.4, 45.3, 52.1, 52.6, 54.2, 60.3, 65.4, 123.3, 123.4, 126.4, 128.4, 129.2, 131.6, 134.0, 134.1, 137.6, 168.0, 168.9 ppm. HRMS *m/z* Calculated [M+H]^+^ for C_29_H_39_N_4_O_4_^+^: 507.2966; found: 507.2954.

**(*S*)-2-(1,3-Dioxoisoindolin-2-yl)-*N*-((2*R*,3*S*)-3-hydroxy-4-(4-methylpiperazin-1-yl)-1-phenylbutan-2-yl)-3-phenylpropanamide** (**6b**): White solid. Yield: 52 %. mp: 101-103 ^o^C. IR (KBr) ν_max_ (cm^-1^): 3385 (OH), 2964, 2929, 1718 (CO), 1654 (CO), 1382, 1070, 718; ^1^H NMR (400 MHz, CDCl_3_): 2.27 (3H, s), 2.22-2.43 (8H, m), 2.64 (2H, m), 2.85-2.88 (2H, m), 3.36-3.50 (2H, m), 3.68-3.70 (1H, m), 4.07 (1H, q, *J* = 7.63 Hz), 5.00 (1H, dd, *J* = 6.10, 9.92 Hz), 6.44 (1H, d, *J* = 8.39 Hz), 7.09-7.15 (10H, m), 7.65-7.67 (2H, m), 7.72-7.74(2H, m) ppm ^13^C NMR (100 MHz, CDCl_3_): 34.5, 38.5, 45.7, 52.0, 54.6, 55.3, 60.1, 65.3, 123.4, 126.3, 126.9, 128.3, 128.6, 128.8, 129.2, 131.4, 134.1, 136.5, 137.6, 167.7, 167.9 ppm. HRMS *m/z* Calculated [M+H]^+^ for C_32_H_37_N_4_O_4_^+^: 541.2809; found: 541.2795.

**(*S*)-2-(1,3-Dioxoisoindolin-2-yl)-*N*-((2*R*,3*S*)-3-hydroxy-4-(4-methylpiperazin-1-yl)-1-phenylbutan-2-yl)-3-methylbutanamide** (**6c**): White solid. Yield: 53 %. mp: 91-93 ^o^C. IR (KBr) ν_max_ (cm^-1^): 3341 (OH), 2952, 1714 (CO), 1681 (CO), 1451, 1382, 1070, 751. ^1^H NMR (400 MHz, CDCl_3_): 0.74 (3H, d, *J* = 6.87 Hz), 0.81 (3H, d, *J* = 6.87 Hz), 2.18-2.20 (3H, m), 2.23 (3H, s), 2.40-2.65 (8H, m), 2.81-2.96 (4H, m), 4.01 (1H, q, *J* = 7.79 Hz), 4.22 (1H, d, *J* = 10.99 Hz), 6.87-7.14 (5H, m), 7.65-7.70 (2H, m), 7.76-7.79 (2H, m) ppm. ^13^C NMR (100 MHz, CDCl_3_): 18.9, 19.7, 26.9, 35.3, 38.2, 43.8, 45.3, 52.0, 54.3, 60.3, 62.5, 65.7, 123.3, 123.5, 126.2, 128.0, 128.2, 128.9, 129.1, 131.3, 134.1, 134.2, 137.7, 167.5, 168.0 ppm. HRMS *m/z* Calculated [M+H]^+^ for C_28_H_37_N_4_O_4_^+^: 493.2809; found 493.2801.

**(2*S*,3*R*)-2-(1,3-Dioxoisoindolin-2-yl)-*N*-((2*R*,3*S*)-3-hydroxy-4-(4-methylpiperazin-1-yl)-1-phenylbutan-2-yl)-3-methylpentanamide** (**6d**): White solid. Yield: 61 %. mp: 104-106 ^o^C. IR (KBr) ν_max_ (cm^-1^): 3343 (OH), 2964, 1717 (CO), 1670 (CO), 1458, 1383, 1072, 753. ^1^H NMR (400 MHz, CDCl_3_): 0.73 (3H, t, *J* = 6.87 Hz), 0.79 (3H, d, *J* = 6.10 Hz), 0.85-0.94 (1H, m), 1.20-1.25 (1H, m), 1.90-2.33 (13H, m), 2.46-2.59 (3H, m), 2.88 (1H, t, *J* = 7.63 Hz), 3.55-3.65 (2H, m), 3.98 (1H, q, *J* = 8.39 Hz), 4.31 (1H, d, *J* = 11.44 Hz), 6.94-7.22 (5H, m), 7.66-7.68 (2H, m), 7.75-7.78 (2H, m) ppm. ^13^C NMR (100 MHz, CDCl_3_): 10.0, 15.7, 25.2, 32.4, 38.1, 45.3, 52.1, 54.2, 60.3, 61.2, 65.8, 123.5, 126.2, 128.0, 128.2, 129.0, 129.1, 131.3, 134.3, 137.7, 168.0, 168.5 ppm. HRMS *m/z* Calculated [M+H]^+^ for C_29_H_39_N_4_O_4_^+^: 507.2966; found 507.2960.

**(*S*)-2-(1,3-Dioxoisoindolin-2-yl)-*N*-((2*R*,3*S*)-4-(4-ethylpiperazin-1-yl)-3-hydroxy-1-phenylbutan-2-yl)-4-methylpentanamide** (**6e**): White solid. Yield: 59 %. mp: 117-119 ^o^C. IR (KBr) ν_max_ (cm^-1^): 3341 (OH), 2963, 1716 (CO), 1683 (CO), 1451, 1390, 1071, 752. ^1^H NMR (400 MHz, CDCl_3_): 0.83 (3H, t, *J* = 6.59 Hz), 1.15 (3H, t, *J* = 7.32 Hz), 1.36-140 (1H, m), 2.20-2.27 (1H, m), 2.45-2.48 (1H, m), 2.66-2.91 (13H, m), 3.79-3.81 (1H, m), 4.11 (1H, q, *J* = 8.05, 1H), 4.78 (1H, dd, J = 4.39, 11.72 Hz), 6.66 (1H, d, J = 8.79 Hz), 7.09-7.25 (5H, m), 7.68-7.72 (2H, m), 7.75-7.79 (2H, m) ppm. ^13^C NMR (100 MHz, CDCl_3_): 10.2, 21.1, 23.1, 25.1, 37.0, 38.0, 50.6, 51.6, 52.4, 60.0, 65.8, 110.7, 118.4, 123.4, 124.1, 125.0, 126.4, 127.5, 128.4, 129.1, 131.6, 134.1, 137.5, 143.0, 168.0, 169.2 ppm. HRMS *m/z* Calculated [M+H]^+^ for C_30_H_41_N_4_O_4_^+^: 521.3122; found 521.3116.

**(*S*)-2-(1,3-Dioxoisoindolin-2-yl)-*N*-((2*R*,3*S*)-4-(4-ethylpiperazin-1-yl)-3-hydroxy-1-phenylbutan-2-yl)-3-phenylpropanamide** (**6f**): White solid. Yield: 55 %. mp: 108-111 ^o^C. IR (KBr) ν_max_ (cm^-1^): 3385 (OH), 3028, 1712 (CO), 1676 (CO), 1456, 1386, 1087, 721, 530. ^1^H NMR (400 MHz, CDCl_3_): 1.15 (3H, m), 2.5 (2H, m), 2.62 (4H, m), 2.76-2.94 (9H, m), 3.39 (1H, m), 3.49 (1H, m), 3.83 (1H, d, *J* = 6.87 Hz), 4.17 (1H, m), 5.02 (1H, dd, *J* = 6.41, 9.62 Hz), 6.55 (1H, d, *J* = 9.2 Hz), 7.05-7.19 (10H, m), 7.74 (4H, m) ppm. ^13^C NMR (100 MHz, CDCl_3_): 10.9, 29.6, 34.6, 38.2, 50.8, 51.8, 52.5, 55.1, 65.6, 123.4, 128.4, 128.6, 128.8, 129.2, 131.4, 134.1, 167.7, 168.1 ppm. HRMS *m/z* Calculated [M+H]^+^ for C_33_H_39_N_4_O_4_^+^: 555.2966; found 555.2954.

**(*S*)-2-(1,3-Dioxoisoindolin-2-yl)-*N*-((2*R*,3*S*)-4-(4-ethylpiperazin-1-yl)-3-hydroxy-1-phenylbutan-2-yl)-3-methylbutanamide** (**6g**): Yield: 62 %. mp: 108-111 ^o^C. IR (KBr) ν_max_ (cm^-1^): 3385 (OH), 2964, 2929, 1718 (CO), 1654 (CO), 1544, 1382, 1070, 718; ^1^H NMR (400 MHz, CDCl_3_): δ 0.73 (3H, d, *J* = 6.6 Hz), 0.84 (3H, d, *J* = 6.6 Hz), 1.08 (3H, t, *J* = 7.36 Hz), 2.26-2.30 (5H, m,), 2.64-2.71 (5H, m), 2.9 (3H, m), 3.7 (1H, t, *J* = 5.88 Hz), 4.05 (1H, q, *J* = 8.04 Hz), 4.24 (1H, d, *J* = 11.00 Hz), 6.8-7.15 (5H, m), 7.71 (2H, m), 7.78 (2H, m) ppm. ^13^C NMR (100 MHz, CDCl_3_): δ 11.2, 19.3, 19.8, 27.1, 38.2, 51.9, 52.0, 60.3, 62.6, 66.0, 123.5, 126.3, 128.3, 129.1, 131.3, 134.3, 137.6, 168.1, 168.4 ppm. HRMS m/z Calculated [M+H]^+^ for C_29_H_39_N_4_O_4_^+^: 507.2966; found: 507.2961.

**(*S*)-2-(1,3-Dioxoisoindolin-2-yl)-*N*-((2*R*,3*S*)-4-(4-ethylpiperazin-1-yl)-3-hydroxy-1-phenylbutan-2-yl)-3-methylbutanamide** (**6h**): White solid. Yield: 71 %. mp: 127-129 ^o^C. IR (KBr) ν_max_ (cm^-1^): 3343 (OH), 2966, 1714 (CO), 1675 (CO), 1533, 1384, 1072, 753. ^1^H NMR (400 MHz, CDCl_3_): 0.78 (3H, t, *J* = 7.33 Hz), 0.82 (3H, d, *J* = 6.41 Hz), 1.07 (3H, t, *J* = 6.87 Hz), 1.30 (2H, m), 2.21 (1H, br s), 2.27 (2H, m), 2.35-2.61 (10H, m), 2.94 (2H, m), 3.74 (1H, t, *J* = 6.64 Hz), 4.07 (1H, q, *J* = 8.24 Hz), 4.36 (1H, d, *J* = 11.45 Hz), 4.45-4.70 (1H, br s), 7.18 (5H, m), 7.72 (2H, m), 7.81 (2H, m) ppm. ^13^C NMR (100 MHz, CDCl_3_): 10.0, 11.2, 15.7, 25.2, 32.4, 33.5, 38.1, 51.8, 51.9, 52.1, 60.3, 61.2, 65.7, 123.5, 126.2, 128.0, 128.2, 129.0, 129.1, 131.3, 134.2, 137.7, 168.0, 168.5 ppm. HRMS *m/z* Calculated [M+H]^+^ for C_30_H_41_N_4_O_4_^+^: 521.3122; found 521.3120.

**(*S*)-*N*-((2*R*,3*S*)-4-(4-(4-Fluorobenzyl)piperazin-1-yl)-3-hydroxy-1-phenylbutan-2-yl)-2-(1,3-dioxoisoindolin-2-yl)-4-methylpentanamide** (**6i**): White solid. Yield: 62 %. mp: 134-136 ^o^C. IR (KBr) ν_max_ (cm^-1^): 3276 (OH), 2926, 1714 (CO), 1675 (CO), 1604, 1510, 1385, 721. ^1^H NMR (400 MHz, CDCl_3_): 0.89 (3H, d, *J* = 3.21 Hz), 0.91 (3H, d, *J* = 2.75 Hz), 1.40 (1H, m), 1.69 (1H, m), 2.28 (1H, m), 2.62 (6H, m), 2.98 (6H, m), 3.46 (2H, s), 4.03 (1H, m), 4.11 (1H, m), 4.82 (1H, dd, *J* = 5.04, 11.45 Hz), 4.9-5.3 (1H, br s), 6.64 (1H, d, *J* = 9.16), 7.00 (1H, m), 7.25 (8H, m), 7.72 (2H, m), 7.82 (2H, m) ppm. ^13^C NMR (100 MHz, CDCl_3_): 21.1, 23.2, 25.2, 37.0, 37.9, 49.9, 52.4, 52.8, 61.2, 64.9, 115.1, 115.3, 123.0, 123.5, 126.5, 128.5, 129.3, 130.6, 131.7, 133.6, 134.1, 168.0, 169.2 ppm. HRMS *m/z* Calculated [M]^+^ for C_35_H_41_FN_4_O_4_^+^: 600.3106; found: 600.3102.

**(*S*)-*N*-((2*R*,3*S*)-4-(4-(4-Fluorobenzyl)piperazin-1-yl)-3-hydroxy-1-phenylbutan-2-yl)-2-(1,3-dioxoisoindolin-2-yl)-3-phenylpropanamide** (**6j**): White solid. Yield: 55%. mp: 121-123 ^o^C. IR (KBr) ν_max_ (cm^-1^): 3386 (OH), 2926, 1709 (CO), 1653 (CO), 1388, 721, 530. ^1^H NMR (400 MHz, CDCl_3_): 2.50-3.09 (12H, m), 3.40 (1H, m), 3.46 (2H, s), 3.60 (2H, m), 4.12 (2H, m), 5.01 (1H, dd, *J* = 8.04, 13.16 Hz), 6.4 (1H, br s), 6.81-7.34 (14H, m), 7.59-7.68 (4H, m); ppm. ^13^C NMR (100 MHz, CDCl_3_): 29.6, 34.5, 35.1, 37.7, 49.4, 52.2, 53.1, 54.9, 55.4, 60.9, 122.9, 115.2, 122.9, 126.2, 126.4, 126.6, 126.8, 128.2, 128.4, 128.5, 128.7, 128.8, 131.4, 133.5, 133.9, 138.5, 167.6, 168.1 ppm. HRMS *m/z* Calculated [M+H]^+^ for C_38_H_40_FN_4_O_4_^+^: 635.3028; found: 635.3024.

**(*S*)-*N*-((2*R*,3*S*)-4-(4-(4-Fluorobenzyl)piperazin-1-yl)-3-hydroxy-1-phenylbutan-2-yl)-2-(1,3-dioxoisoindolin-2-yl)-3-methylbutanamide** (**6k**): White solid. Yield: 71 %. mp: 137-139 ^o^C. IR (KBr) ν_max_ (cm^-1^): 3356 (OH), 2928, 1717 (CO), 1669 (CO), 1387, 1071, 720, 530. ^1^H NMR (400 MHz, DMSO): 0.62 (3H, d, *J* = 6.87 Hz), 0.77 (3H, d, *J* = 6.41 Hz), 2.16 (10H, m), 2.46-2.68 (5H, m), 3.44 (2H, m), 3.90 (1H, m), 4.13 (1H, d, *J* = 10.07 Hz), 4.51 (1H, br s), 6.88-7.00 (7H, m), 7.17 (2H, m), 7.75 (4H, m) ppm. ^13^C NMR (100 MHz, DMSO): 19.2, 20.0, 26.3, 36.5, 52.4, 53.1, 53.3, 60.6, 61.0, 67.4, 79.1, 114.7, 114.9, 123.2, 125.6, 127.8, 128.9, 130.5, 130.6, 131.0, 134.6, 139.0, 167.3, 167.6 ppm. HRMS *m/z* Calculated [M+H]^+^ for C_34_H_40_FN_4_O_4_^+^: 587.3028; found: 587.3017.

**(*S*)-2-(1,3-Dioxoisoindolin-2-yl)-*N*-((2*R*,3*S*)-4-(4-(4-fluorobenzyl)piperazin-1-yl)-3-hydroxy-1-phenylbutan-2-yl)-3-methylbutanamide** (**6l**): White solid. Yield: 43 %. mp: 112-115 ^o^C. IR (KBr) ν_max_ (cm^-1^): 3338 (OH), 2958, 1713 (CO), 1676 (CO), 1510, 1385, 1222, 755. ^1^H NMR (400 MHz, CDCl_3_): 0.89 (3H, d, *J* = 2.2 Hz), 0.91 (3H, d, *J* = 2.2 Hz), 1.40 (1H, m), 1.70 (1H, m), 2.28, (1H, m), 2.5-2.7 (6H, m), 2.78 (6H, m), 3.49 (2H, m), 3.95 (1H, d, *J* = 8.8 Hz), 4.14 (1H, q, *J* = 8.05 Hz), 4.82 (1H, dd, *J* = 5.13, 11.72 Hz), 6.58 (1H, d, *J* = 9.52 Hz), 7.00 (2H, t, *J* = 8.79 Hz), 7.24 (7H, m), 7.74 (2H, m), 7.82 (2H, m) ppm. ^13^C NMR (100 MHz, CDCl_3_): 21.2, 23.1, 25.2, 37.1, 38.2, 51.3, 52.4, 52.5, 60.9, 61.5, 65.1, 115.0, 115.2, 123.4, 126.4, 128.4, 129.2, 130.5, 130.6, 131.6, 134.1, 137.5, 168.0, 169.1 ppm. HRMS *m/z* Calculated [M]^+^ for C_35_H_41_FN_4_O_4_^+^: 600.3112; found: 600.3102.

**(*S*)*-N*-((2*R*,3*S*)-4-(4-(4-Bromobenzyl)piperazin-1-yl)-3-hydroxy-1-phenylbutan-2-yl)-2-(1,3-dioxoisoindolin-2-yl)-4-methylpentanamide** (**6m**): White solid. Yield: 75 %. mp: 143-145 ^o^C. IR (KBr) ν_max_ (cm^-1^): 3368 (OH), 2956, 2815, 1714 (CO), 1684 (CO), 1386, 1070, 721. ^1^H NMR (400 MHz, CDCl_3_): 0.88 (3H, d, *J* = 2.29 Hz), 0.90 (3H, d, *J* = 1.83 Hz), 1.4 (1H, m), 1.66-1.71 (1H, m), 2.27 (3H, m), 2.40 (6H, m), 2.61 (2H, m), 2.92 (2H, m), 3.40 (2H, s), 3.71 (1H, m), 4.06 (1H, m), 4.81 (1H, dd, *J* = 5.04, 10.99 Hz), 6.47 (1H, m), 7.15 (4H, m), 7.19 (3H, d, *J* = 4.6 Hz), 7.40 (2H, d, *J* = 8.24 Hz), 7.71 (2H, m), 7.81 (2H, m) ppm. ^13^C NMR (100 MHz, CDCl_3_): 21.2, 23.1, 25.2, 29.6, 37.1, 38.6, 52.0, 52.59, 52.7, 60.3, 62.0, 65.2, 120.8, 123.4, 126.3, 128.3, 129.2, 130.6, 131.3, 131.6, 134.1, 136.9, 137.7, 167.9, 168.8 ppm. HRMS *m/z* Calculated [M+H]^+^ for C_35_H_42_BrN_4_O_4_^+^: 661.2384; found: 661.2378.

**(*S*)-*N*-((2*R*,3*S*)-4-(4-(4-Bromobenzyl)piperazin-1-yl)-3-hydroxy-1-phenylbutan-2-yl)-2-(1,3-dioxoisoindolin-2-yl)-3-phenylpropanamide** (**6n**): White solid. Yield: 69 %. mp: 131-133 ^o^C. IR (KBr) ν_max_ (cm^-1^): 3322 (OH), 2929, 1718 (CO), 1675 (CO), 1387, 1009, 721. ^1^H NMR (400 MHz, CDCl_3_): 2.47-2.49 (2H, m), 2.60-2.78 (8H, m), 3.46-3.50 (3H, m), 3.87 (1H, d, *J* = 8.79 Hz), 4.10 (1H, q, *J* = 7.32 Hz), 5.01 (1H, dd, *J* = 6.60, 10.24 Hz), 6.57 (1H, d, *J* = 8.80 Hz), 7.10-7.20 (12H, m), 7.41-7.44 (2H, m), 7.66-7.68 (2H, m), 7.73-7.75 (2H, m) ppm. ^13^C NMR (100 MHz, CDCl_3_): 34.6, 38.2, 51.6, 52.4, 52.7, 55.1, 60.8, 61.7, 65.2, 123.4, 126.9, 128.3, 128.6, 128.8, 129.2, 130.6, 131.4, 134.1, 136.5, 167.7, 168.1 ppm. HRMS *m/z* Calculated [M+H]^+^ for C_38_H_40_BrN_4_O_4_^+^: 695.2227; found: 695.2210.

**(*S*)-*N*-((2*R*,3*S*)-4-(4-(4-Bromobenzyl)piperazin-1-yl)-3-hydroxy-1-phenylbutan-2-yl)-2-(1,3-dioxoisoindolin-2-yl)-3-methylbutanamide** (**6o**): White solid. Yield: 73 %. mp: 160-162 ^o^C. IR (KBr) ν_max_ (cm^-1^): 3334 (OH), 3028, 1714 (CO), 1681 (CO), 1385, 1200, 721. ^1^H NMR (400 MHz, CDCl_3_): 0.68 (3H, d, *J* = 6.59 Hz), 0.72 ( 3H, d, *J* = 6.59 Hz), 1.09 (1H, m), 2.9 (3H, m), 3.29 (3H, m), 3.47-3.79 (10H, m), 4.21 (4H, m), 7.05 (2H, m), 7.14 (2H, m), 7.32 (2H, d, *J* = 8.04 Hz), 7.35 (1H, m), 7.56 (2H, d, *J* = 8.04 Hz), 7.72 (2H, m), 7.76 (2H, m), 8.03 (1H. s); ppm. ^13^C NMR (100 MHz, CDCl_3_): 19.1, 19.5, 27.5, 32.1, 37.0, 37.3, 48.0, 53.5, 60.1, 61.8, 66.6, 123.6, 125.4, 128.6, 128.9, 131.0, 132.5, 132.8, 134.5, 168.5, 170.1 ppm. HRMS *m/z* Calculated [M]^+^ for C_34_H_39_BrN_4_O_4_^+^: 646.2149; found: 646.2132.

**(*S*)-*N*-((2*R*,3*S*)-4-(4-(4-Bromobenzyl)piperazin-1-yl)-3-hydroxy-1-phenylbutan-2-yl)-2-(1,3-dioxoisoindolin-2-yl)-3-methylbutanamide** (**6p**): White solid. Yield: 70 %. mp: 128-130 ^o^C. IR (KBr) ν_max_ (cm^-1^): 3423 (OH), 2928, 1704 (CO), 1684 (CO), 1389, 1199, 1135, 720. ^1^H NMR (400 MHz, CDCl_3_): 0.67 (3H, d, *J* = 6.59 Hz), 0.75 (3H, t, *J* = 7.32 Hz), 0.92-1.12 (2H, m), 2.32 (1H, m), 2.95 (3H, m), 3.04 (1H, m), 3.19 (1H, m), 3.35 (5H, br s), 3.61 (4H, m), 4.11 (3H, m), 4.33 (1H, d, *J* = 11.00 Hz), 6.8 (1H, br s), 7.06 (2H, m), 7.16 (2H, d, *J* = 7.32 Hz), 7.28 (3H, m), 7.53 (2H, d, *J* = 7.32 Hz), 7.71 (2H, m), 7.78 (2H, m) ppm. ^13^C NMR NMR (100 MHz, CDCl_3_): 10.1, 15.6, 25.2, 29.5, 33.0, 37.3, 48.1, 53.3, 60.1, 60.3, 60.9, 66.4, 123.6, 126.7, 128.6, 129.1, 131.2, 132.3, 132.6, 134.4, 168.5, 169.7 ppm. HRMS *m/z* Calculated [M]^+^ for C_35_H_41_BrN_4_O_4_^+^: 660.2306; found: 660.2293.

**(*S*)*-N*-((2*R*,3*S*)-4-([1,4'-Bipiperidin]-1'-yl)-3-hydroxy-1-phenylbutan-2-yl)-2-(1,3-dioxoisoindolin-2-yl)-3-phenylpropanamide** (**6q**): White solid. Yield: 61 %. mp: 134-136 ^o^C. IR (KBr) ν_max_ (cm^-1^): 3390 (OH), 2985, 2941, 2823, 1714 (CO), 1687 (CO), 1517, 1361, 1247, 1051, 710. ^1^H NMR (400 MHz, CDCl_3_): 1.59 (6H, d, *J* = 1.04 Hz), 1.87 (4H, m), 2.18 (1H, m), 2.26 (3H, m), 2.43 (1H, m), 2.50 (4H, m), 2.92 (5H, m), 3.84 (4H, m), 5.00 (1H, dd, *J* = 3.64, 10.24 Hz), 6.58 (1H, d, *J* = 8.8 Hz), 7.16 (10H, m), 7.68 (2H, m), 7.73 (2H, m) ppm. ^13^C NMR (100 MHz, CDCl_3_): 22.8, 23.3, 24.1, 34.6, 35.3, 49.3, 52.7, 54.9, 55.5, 60.5, 65.4, 122.9, 123.4, 128.2, 128.4, 128.7, 128.8, 129.2, 133.5, 134.1, 167.7, 168.2 ppm. HRMS *m/z* Calculated [M]^+^ for C_37_H_44_N_4_O_4_^+^: 608.3363; found: 608.3356.

**(2*S*,2'*S*)-*N*,*N*'-((2*R*,2'*R*,3*S*,3'*S*)-Piperazine-1,4-diylbis(3-hydroxy-1-phenylbutane-4,2-diyl))bis(2-(1,3-dioxoisoindolin-2-yl)-4-methylpentanamide)** (**6r**): White solid. Yield: 63 %. mp: 141-143 ^o^C. IR (KBr) ν_max_ (cm^-1^): 3386 (OH), 2981, 2936, 2806, 1715 (CO), 1686 (CO), 1517, 1366, 1247, 1171, 1046, 694. ^1^H NMR (400 MHz, CDCl_3_): 0.90 (12H, d, *J* = 6.87 Hz), 1.70 (2H, m), 2.24 (2H, m), 2.29 (8H, m), 2.54 (4H, s), 2.91 (6H, m), 3.66 (4H, m), 4.05 (2H, q, *J* = 8.24 Hz), 4.79 (1H, dd, *J* = 1.83, 5.04 Hz), 4.82 (1H, dd, *J* = 1.83, 5.04 Hz), 6.47 (2H, d, J = 9.16 Hz), 7.25 (10H, m), 7.71 (4H, m), 7.81 (4H, m) ppm. ^13^C NMR (100 MHz, CDCl_3_): 21.1, 23.0, 25.1, 28.2, 37.1, 38.6, 39.3, 40.8, 51.8, 52.6, 53.1, 60.1, 60.3, 65.3, 79.0, 123.4, 126.3, 128.2, 128.3, 129.2, 129.3, 131.6, 134.1, 137.7, 155.7, 167.9, 168.8 ppm. HRMS *m/z* Calculated [M+H]^+^ for C_52_H_63_N_6_O_8_^+^: 899.4702; found: 899.4684.

**(2*S*,2'*S*)-*N*,*N*'-((2*R*,2'*R*,3*S*,3'*S*)-Piperazine-1,4-diylbis(3-hydroxy-1-phenylbutane-4,2-diyl))bis(2-(1,3-dioxoisoindolin-2-yl)-3-methylbutanamide)** (**6t**): White Solid. Yield: 44 %. mp: 123-125 ^o^C. IR (KBr) ν_max_ (cm^-1^): 3359 (OH), 3060, 2929, 1718 (CO), 1675 (CO), 1529, 1384, 1157, 1070, 717, 531. ^1^H NMR (400 MHz, DMSO): 0.72 (6H, d, *J* = 5.86 Hz), 0.88 (6H, d, *J* = 5.13 Hz), 2.17-2.41 (10H, m), 2.55-2.74 (8H, m), 3.5 (2H, s), 3.97 (2H, m), 4.24 (2H, d, *J* = 10.25 Hz), 4.73 (2H, br s), 7.08 (10H, m), 7.57 (2H, d, *J* = 8.76 Hz), 7.86 (8H, s) ppm. ^13^C NMR (100 MHz, DMSO) 19.3, 20.1, 26.3, 36.5, 53.1, 53.4, 60.6, 67.5, 123.3, 125.7, 127.9, 128.9, 131.1, 134.7, 139.0, 167.3, 167.6 ppm. HRMS *m/z* Calculated [M+H]^+^ for C_50_H_59_N_6_O_8_^+^: 871.4389; found: 871.4365.

**(2*S*,2'*S*,3*R*,3'*R*)-*N*,*N*'-((2*R*,2'***R***,3*S*,3'*S*)-Piperazine-1,4-diylbis(3-hydroxy-1-phenylbutane-4,2-diyl))bis(2-(1,3-dioxoisoindolin-2-yl)-3-methylpentanamide)** (**6u**): White Solid. Yield: 41 %. mp: 131-134 ^o^C. IR (KBr) ν_max_ (cm^-1^): 3361 (OH), 3058, 2928, 1715 (CO), 1678 (CO), 1499, 1383, 1158, 1073, 717. ^1^H NMR (400 MHz, CDCl_3_): 0.08 (6H, t, *J* = 7.79 Hz), 0.85 (6H, d, *J* = 6.41 Hz), 0.91-1.02 (2H, m), 1.28-1.34 (2H, m), 2.06 (4H, d, *J* = 7.79 Hz), 2.15 (4H, m), 2.29-2.62 (8H, m), 2.95 (4H, m), 3.66 (2H, m), 4.07 (2H, q, *J* = 7.79 Hz), 4.37 (2H, d, *J* = 11.45 Hz), 6.95 (2H, d, *J* = 9.16 Hz), 7.07 (2H, m), 7.15 (4H, m), 7.20 (4H, d, *J* = 6.87 Hz), 7.76 (4H, m), 7.84 (4H, m) ppm. ^13^C NMR (100 MHz, DMSO) 10.1, 15.7, 25.3, 32.4, 38.3, 51.9, 60.3, 61.3, 65.8, 123.5, 126.2, 128.2, 129.2, 131.3, 134.3, 137.8, 168.0, 168.5 ppm. HRMS *m/z* Calculated [M+H]^+^ for C_52_H_63_N_6_O_8_^+^: 899.4702; found: 899.4695.
