# Supplementary material for: Hydroxyethylamine Based Phthalimides as New Class of Plasmepsin Hits: Design, Synthesis and Antimalarial Evaluation
Source: PLoS One. 2015 Oct 26;10(10):e0139347. doi: 10.1371/journal.pone.0139347 (PMC4621027; doi:10.1371/journal.pone.0139347)
Supplement: S3 Text — (DOCX) [file pone.0139347.s056.docx]

**S1 Reference**

S1. Rathi B, Singh AK, Kishan R, Singh N, Latha N, Srinivasan, S *et al.* Functionalized hydroxyethylamine based peptide nanostructures as potential inhibitors of falcipain-3, an essential proteases of *Plasmodium falciparum*. Bioorg Med Chem 2013; 21: 5503-9.
